# Multifactorial Analysis of Conditional Reprogramming of Human Keratinocytes

**DOI:** 10.1371/journal.pone.0116755

**Published:** 2015-02-25

**Authors:** Segni B. Ligaba, Anikita Khurana, Garrett Graham, Ewa Krawczyk, Sandra Jablonski, Emanuel F. Petricoin, Robert I. Glazer, Geeta Upadhyay

**Affiliations:** 1 Department of Pathology, Georgetown University, Washington, DC, 20007, United States of America; 2 Department of Oncology, Georgetown University, Washington, DC, 20007, United States of America; 3 Lombardi Comprehensive Cancer Center, Georgetown University, Washington, DC, 20007, United States of America; 4 Department of Molecular and Microbiology, George Mason University, Manassas, Virginia, 20110, United States of America; University of Tennessee, UNITED STATES

## Abstract

Co-culture of human primary epithelial cells with irradiated 3T3 fibroblast feeder cells (J2 cells) and the Rho kinase inhibitor Y-27632 (Y) allows for the unrestricted growth of cells of epithelial origin by the process termed conditional reprogramming. To better understand the nature of the signaling processes associated with conditionally reprogrammed cells, the effect of the two critical components of the co-culture conditions, J2 cells and Y, on the growth of human foreskin keratinocytes (HFKs) was evaluated by gene expression profiling, reverse-phase protein arrays and siRNA screening. J2 cells and Y acted cooperatively to down-regulate differentiation, and upregulate proliferation and cell adhesion, including increased pT308Akt and pERK, and reduced TGF-β pathway signaling. These findings establish a mechanistic basis for the unlimited growth potential of human epithelial cells that will be invaluable to assess the effect of genetic changes in pathologic tissues and their response to therapeutic agents.

## Introduction

The capacity of human epidermal keratinocytes for sustained growth depends on the relative cell composition emanating from the basal layer [1–3]. When keratinocytes are co-cultured with irradiated 3T3 fibroblast feeder cells (J2 cells) and growth factors [1, 4, 5], they undergo continuous replication over many generations resulting in their “immortalization”. Recently, this co-culture technique was modified to include epithelial cells from prostate, breast, trachea, liver and lung, wherein the use of J2 cells and the Rho kinase inhibitor Y-27632 (Y) resulted in the rapid reprogramming of cells into immortalized karyotype-stable cultures [6, 7]. These conditionally reprogrammed epithelial cells expressed markers of adult stem cells, but not of embryonic and induced pluripotent stem cells, and appeared to consist of cell populations resembling their primary tissue of origin, making them ideal to study tissue regeneration, as well as normal physiologic and pathologic processes [8]. This technique was employed recently to examine the differential drug response of tissue acquired from a patient with recurrent respiratory papillomatosis to identify a drug that was effective in controlling this disease [9]. With this goal in mind, we sought to define the signaling pathways associated with J2 cells and Y that are paramount to the immortalization process. Using gene expression profiling, functional siRNA library screening and reverse-phase protein arrays (RPPA), we found that J2 cells and Y produced a cooperative effect on human foreskin keratinocytes (HFKs), which resulted in suppression of TGF-β signaling juxtaposed with up-regulation of changes in cell cycle progression, cell adhesion and motility. This information provides an important basis for understanding the processes that contribute to immortalization using this technique.

## Materials and Methods

### Cells

Human keratinocytes were cultured from discarded and de-identified neonatal foreskin tissues. This research study was approval by the Institutional Review Board, Georgetown University. The details are previously described [6–8]. Briefly, the epithelial layer was removed, minced with sterile scissors and digested with 0.25% trypsin at 37°C for 10 min on a rotating shaker. Digestion was stopped by the addition of medium containing 5% FBS and cells were collected by centrifugation at 3,000 rpm at 4C for 5 min. HFKs were maintained at 37°C under 5% CO_2_ in F medium (F-12:DMEM, 3:1) containing: 5% FBS, 0.4 μg/ml hydrocortisone, 5 μg/ml insulin, 8.4 ng/ml cholera toxin, 10 ng/ml EGF, 24 μg/ml adenine, 100 U/ml penicillin, 100 μg/ml streptomycin, 5 μM Y (Enzo Life Sciences). HFKs were co-cultured with irradiated (3,000 Rad) J2 cells (kindly provided by Dr. Richard Schlegel, Georgetown University) derived from mouse NIH 3T3 fibroblasts at a 1:4 HFK:J2 cell ratio, and sub-cultured when they reached 85% confluence. J2 cells were removed by differential trypsinization for 30 sec with gentle shaking, and HFKs were washed with PBS and incubated with 0.01% trypsin for 2 min. Population doubling time was determined by the interval between cell passages at 85% confluence.

### siRNA screening

The siRNA library to assess factors secreted by J2 cells was comprised of 332 fully annotated genes based on the 3T3 cell transcriptome in GEO database GSM1348503. The library contained two siRNA sequences per gene/well in 96-well plates at a concentration of 100 pmoles siRNA/well (1 μM) (Table B in S1 File). J2 cells were grown in F medium supplemented with 5 μM Y and reverse-transfected with the siRNA library using Lipofectamine RNAiMax reagent (Invitrogen) at a final concentration of 20 nM. Alexafluor-488-tagged siRNAs were used to determine transfection efficiency. Screening was conducted with robotic liquid handlers, including a Thermo-Matrix Well-Mate bulk reagent dispenser, a CyBio automated pipettor with exchangeable multiwall head, a Thermo-Matrix Combi nl bulk reagent dispenser and a PerkinElmer EnVision plate reader. Screening was done in duplicate and repeated twice.

Two days following transfection, the response of HFKs to the resulting conditioned medium (CM) following transfection was assessed by colony assay. CM was centrifuged at 3,000 rpm at 4°C, passed through a 0.2 μM sterile filter and frozen in aliquots at-80°C until use. The presence of J2 cells and Y were optimal for colony formation (Fig. A. I in S1 File), and the effect of CM on colony formation was proportional to the amount of CM added to the dish (Fig. A.II in S1 File).

### Colony assay

HFKs (5,000/well) were seeded into a 6-well plate in 2 ml of CM. Colonies were grown for 5 days prior to fixation and quantified using the sulforhodamine B (SRB) assay [10]. Briefly, cells were fixed with 10% TCA at 4°C for 1 hr, washed with deionized water, stained with 0.4% SRB in 1% acetic acid for 1 hr on a rotary shaker, washed with1% acetic acid and air dried. Colonies were resuspended in 10 mM Tris, pH 10.0 and absorbance was measured at 490 nm with a Beckman spectrophotometer.

### Gene expression microarray analysis

RNA was isolated with an RNeasy Mini Kit (Qiagen) according to the manufacturer’s protocol. RNA quality, cRNA synthesis and hybridization was carried out by the Genomics and Epigenomics Shared Resource (GESR), Lombardi Comprehensive Cancer Center (LCCC), Georgetown University, using an Affymetrix Human U133 Plus 2.0 GeneChip according to the manufacturer’s protocol. Hybridization signals were detected with an Agilent Gene Array scanner, and grid alignment and raw data generation used Affymetrix GeneChip Operating software 1.1. Changes in gene expression ≥3-fold were clustered hierarchically with CIMiner software (National Cancer Institute, NIH). Gene interaction and ontology analysis utilized Ariadne Pathway Studio version 9.1. Data sets have been deposited in the GEO public database under accession no. GSE61226.

### Quantitative real-time polymerase chain reaction (qRT-PCR)

Total RNA was extracted using the RNAeasy Mini Kit (Qiagen) according to the manufacturer’s protocol. One µg of RNA was reverse-transcribed in a total volume of 20 µl using the Cloned AMV First-Strand cDNA Synthesis kit (Invitrogen). PCR was performed in triplicate in an ABI 7900 instrument (Applied Biosystems) using SYBRGreen I detection (Qiagen) according to the manufacturer’s protocol.

### Reverse-phase protein array (RPPA) analysis

J2 cells were removed from a 10-cm dish by incubating with 0.02% EDTA in PBS for 5 min at 37°C. HFKs were directly lysed in T-PER Tissue Protein Extraction Reagent (Pierce). Protein lysates were immobilized on a solid phase membrane, probed with 106 validated antibodies, tagged with secondary antibodies and the signal amplified as previously described [11, 12].

### Western blotting

J2 cells were removed from a 10-cm dish with 0.02% EDTA. HFKs were scraped into buffer containing: 50 mM Tris-HCl (pH 7.5), 0.5% NP-40, 0.1% SDS, 0.25% sodium deoxycholate, 125 mM NaCl, 1 mM EDTA, 1 mM EGTA, 10% glycerol, 50 mM NaF, 1 mM sodium orthovanadate, 2.5 mM sodium pyrophosphate, 1 mM sodium β-glycerophosphate, 1 mM PMSF, and a protease inhibitor cocktail (Roche Molecular Biochemicals) at 4°C. Protein lysis, quantification, sample preparation, SDS-PAGE electrophoresis and Western blotting with a SNAP I.D. protein detection system (Millipore) was performed as previously described [13, 14]. A list of primary antibodies is provided in Table A in S1 File.

### Statistics

Statistical analyses of paired data were analyzed by the two-tailed Student’s *t*-test, and between groups of two or more variables by the two-tailed Fisher’s Exact test. Differences were considered significant at *P*<0.05.

## Results

### J2 feeder cells and Y-27632 facilitate cell cycle transition

**Figure 1:**
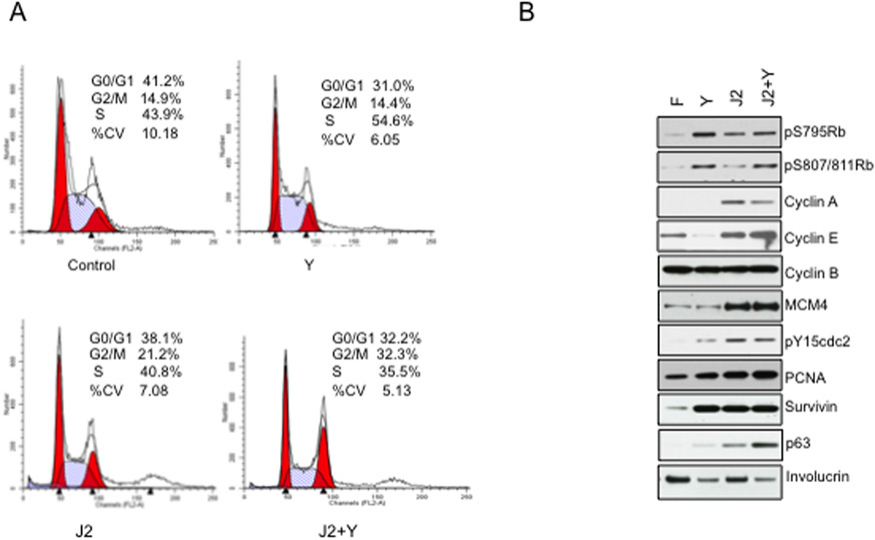
Cell cycle transition of conditionally reprogrammed HFKs. *A*, HFKs at passage 10 were grown in the presence of either Y-27632 (Y), J2 cells (J2) or J2 cells and Y (J2+Y). Conditional reprograming of HFKs increased G2-M transition. FACS indicated that the combination of J2 cells and Y (J2+Y) increased the percentage of cells in G2-M to 32.3%. *B*, Co-culture of HFKs with J2 cells maintained the level of cell cycle regulators. J2 cells up-regulated cyclins A and E, MCM4 and pCDK1, as well as the stem cell marker p63 vs. Y alone, and reduced expression of the squamous epithelial marker Involucrin.

The Rho kinase inhibitor Y-27632 has been reported to reduce stress fibers and the actin microarchitecture [15, 16], however cells in coculture do not depict its negative effect on cell growth. Cell cycle analysis showed that HFKs grown in the presence of Y exhibited an increase in S phase transition, whereas co-culture with J2 cells increased G2-M, an effect that was further enhanced in the presence of Y (Fig. 1A). The latter conditions maintained the levels of pRb, cyclin A, cyclin E, MCM4 and pCDK1 (Fig. 1B, Fig. B in S1 File). Additionally, J2 cells and Y increased expression of the stem cell marker, p63, and reduced expression of the stratified squamous epithelial cell marker, Involucrin (Fig. 1B, Fig. B in S1 File). Conditional reprogramming did not result in major changes in apoptosis as detected by Annexin V expression and the absence of caspase9 and caspase3 cleavage (Fig. C. I in S1 File). Autophagy was reduced by J2 cells as shown by the absence of cleavage of LC3B, an ubiquitin-like modifier involved in formation of autophagosome vacuoles (Fig. C. II in S1 File).

### J2 cells and Y reduce the expression keratinocyte differentiation genes and upregulate expression of cell adhesion and proliferative genes

**Figure 2:**
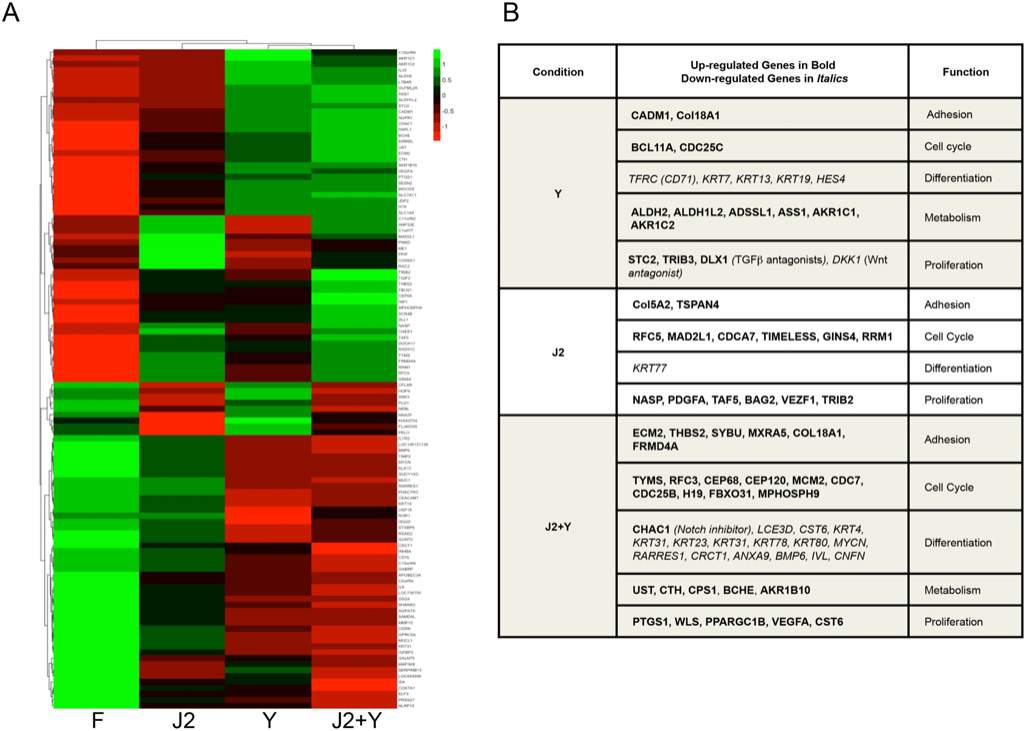
Gene expression signature of conditionally reprogrammed HFKs. *A*, Heatmap of the top 30 up- and down-regulated genes based on the changes in the ratio of Y/F, J2/F and J2Y/F. *B*, Gene expression signatures related to the effects of Y, J2 or J2+Y. A list of changes in gene expression is in Table C in S1 File.

To obtain a global picture of gene transcription resulting from conditional reprogramming, gene expression analysis of freshly isolated HFKs grown for two days with or without J2 cells and Y was evaluated. Gene expression analysis revealed that treatment with Y differentially regulated 293 genes, whereas, J2 cells modulated 215 genes, and the combination of J2 cells and Y differentially affected 495 genes (Table C in S1 File). A heatmap of the expression of 30 genes from each of the three comparisons that demonstrated the greatest degree of positive and negative regulation indicated unique as well as cooperative effects between J2 cells and Y on HFKs (Fig. 2A). The distinct effects of Y and J2 cells were achieved by different sets of genes associated with increased proliferation and reduced differentiation and other processes (Fig. 2B). A list of the changes in gene expression is shown in Table C in S1 File.

### Secretory factors associated with J2 cells

**Figure 3:**
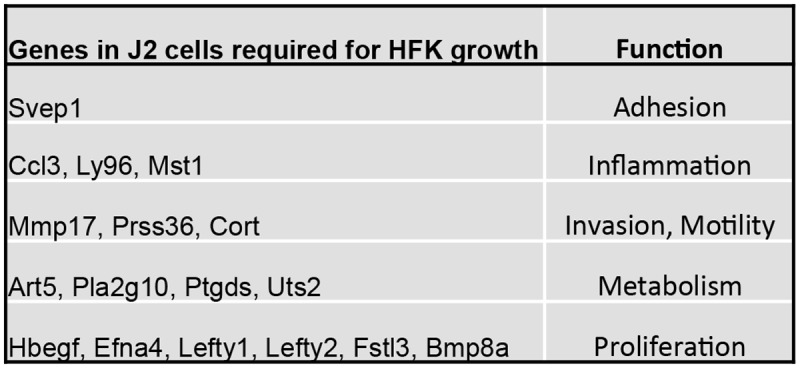
Functional siRNA library screening of J2 cells for secreted factors. J2 cells were grown in the presence of Y, and transfected with a library of 332 siRNAs. The growth response of HFKs to the each conditioned medium (CM) resulting from each RNA ‘knockdown’ was evaluated two days after transfection by colony assay. Listed are genes whose ‘knockdown’ resulted in ≥50% reduction in growth.

J2 cells secreted factors into the ‘conditioned’ medium (CM) that were able to substitute for the use of feeder cells in conditional reprogramming [17]. To determine the nature of these factors, J2 cells were screened for their growth effects on HFKs using a siRNA library targeting 332 genes producing secreted factors that are expressed by 3T3 fibroblasts, the cell line from which J2 cells were originally derived (Table B in S1 File). The effect of gene ‘knockdown’ in J2 cells was determined by the effect of the CM corresponding to each siRNA on HFK colony formation. Knockdown of 14 genes was associated with growth suppression by their corresponding CM, indicating that in the absence of knockdown, these genes were responsible for growth stimulation (Fig. 3). These genes were associated with up-regulating cell adhesion, inflammation, invasion, motility, metabolism and proliferation (Fig. 3). Also noted as positive regulators of cell growth in the CM were negative regulators of TGF-β signaling (Fstl3, Lefty1, Lefty2) (Fig. 3). The net effect of these secreted factors affected signaling pathways associated with F-actin organization, cytoskeleton modification, Smads, GPCRs and Ephrin signaling (Fig. D in S1 File).

### Posttranslational networks associated with conditional reprogramming

**Figure 4:**
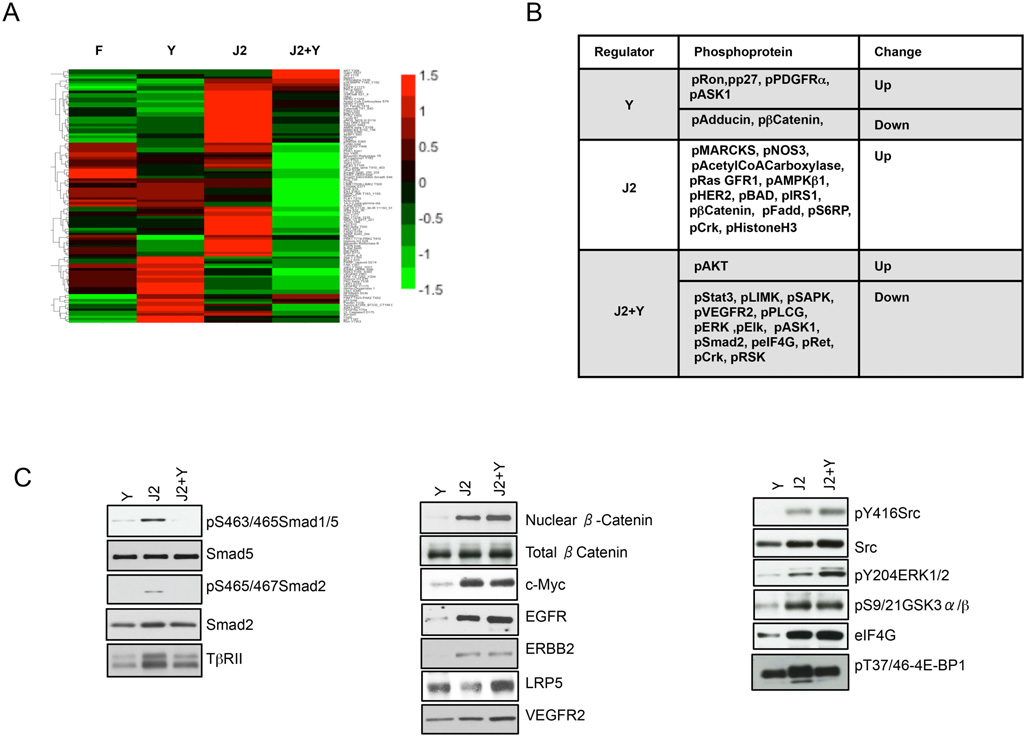
Post-translational changes associated with conditional reprogramming. *A*, Heatmap of the post-translational changes occurring in HFKs after 2 days in culture with J2 cells (J2), Y or J2 cells and Y (J2+Y). Shown are proteins that were modified ≥50% by each condition as determined by RPPA analysis. *B*, Summary of RPPA analysis. Co-culture of HFKs with J2 cells and Y preferentially increased pAkt, and reduced phosphorylation of 13 proteins, including Smad2. *C*, Western analysis of conditionally reprogrammed HFKs. J2 cells prevented the reduction of pSmad1/5, pSmad2, TGF-β receptor II (TβRII), nuclear β-catenin, c-Myc, EGFR, ERBB2, VEGFR2, pSrc, pERK, pGSK3β, eIF4G and p4EFBP by Y.

To obtain a better picture of signaling networks associated with conditional reprogramming, posttranslational changes were assessed by RPPA (Fig. 4A, B; Table D in S1 File). J2 cells in combination with Y increased the level of pT308Akt and reduced the levels of 14 phosphoproteins. Y alone increased expression of pY1353Ron, pT187p27, pY754PDGFRα and pS83ASK1; J2 cells increased expression of 15 phosphoproteins, particularly pMARCKS and pAdducin (Table D in S1 File). The most notable change in common among the three conditions was reduced Smad1/2/5/8 phosphorylation (Fig. 4C, Fig. E in S1 File), and was consistent with the functional siRNA screening data from J2 cells (Fig. 3) as well as the gene expression profiling of conditionally reprogrammed HFKs (Fig. 2). Western blotting confirmed that Smad phosphorylation was reduced by Y, and that the levels of c-Myc, EGFR, ERBB2, pY416Src, pS9GSK3β, eIF4G and pT37/414EFBP were increased by J2 cells (Fig. 4C, Fig. E in S1 File).

## Discussion

A novel method to conditionally reprogram cells of epithelial origin combines the use of J2 feeder cells with the ROCK inhibitor Y for expanding cell cultures of primary tissue and maintaining a stable karyotype and cellular heterogeneity [6–8, 17]. The utility of this method lies in its ability to use primary human tissues to study processes such as transformation and chemoprevention, and to assess chemosensitivity and resistance in malignant tissues of individual patients [18]. The utility of this technique was shown recently by its use to identify a drug to treat a patient with recurrent respiratory papillomatosis [9]. Recently, an alternate tissue culture technique for circulating tumor cells was described for the similar objective of assessing drug sensitivity and individualizing therapy [19]. Although, there are potentially beneficial applications of conditional reprogramming, the nature of the changes produced by the secreted factors from feeder cells in conjunction with Y, and their associated signaling networks remain to be elucidated. To address these questions, the effect of J2 cells and Y were assessed individually and in combination using multiple approaches, including gene expression profiling, siRNA functional screening of J2 cells and RPPA analysis of the post-translational changes in phosphorylation that accompany conditional reprogramming.

Previous studies have shown that ROCK inhibition by Y disrupts the stress fiber and actin cytoskeletal microarchitecture [15, 16]. However, cells in coculture do not show any growth retardation and of the factors secreted by J2 cells have a positive affect on actin filament organization (Fig. D in S1 File), as shown by attenuation of Adducin and MARCKS phosphorylation (Fig. 4B; Table D in S1 File), processes associated with destabilization of F-actin capping [20].

The individual effects of J2 cells and Y on cell cycle progression were evidenced by an increase in S phase by Y, and an increase in G2/M by J2 cells (Fig. 1). Whereas, J2 cells increased cyclin A and cyclin E expression, J2 cells and Y increased Rb and CDK1 phosphorylation and elicited a proliferative gene expression profile (Fig. 2). This was contributed in part by secretion of HBEGF and other growth factors by J2 cells, as well as by increased EGFR, VEGFR2 and HER2 expression, and their downstream signaling through pERK and pAkt.

In addition to the proliferative changes during conditional reprogramming, a marked reduction in differentiation occurred. This effect was initially noted almost 40 years ago when feeder cells were first used to propagate human keratinocytes for a limited duration [21, 22]. However, the addition of Y to the culture medium completely blocked differentiation, and as shown in the present study, altered the cellular milieu of secreted factors from J2 cells that favored immortalization. Interestingly, we found that reduced differentiation correlated with enhanced expression of the Notch inhibitor CHAC1, and reduced expression of the Notch target gene Hes4. Y was shown previously to reduce keratinocyte differentiation gene expression [23], and reduce non-canonical Notch-mediated cell differentiation, which led to loss of Involucrin and increased p63 expression [24]. Considering the importance of Notch signaling in keratinocyte differentiation [22], the negative regulatory effects of J2 cells and Y on this process likely occur in part through inhibition of this pathway.

One of the seminal findings in the present study was the identification of TGF-β pathway suppression as a major outcome of conditional reprogramming. TGF-β and related ligands signal through Smads to promote tissue homeostasis through modulation of proliferation, differentiation and apoptosis [25, 26]. This was evident by siRNA screening of J2 cells which identified increased secretion of the Activin/Smad2 antagonists Fstl3, Lefty1 and Lefty2 [27] (Fig. 3), and gene expression profiling demonstrating increased expression of the Smad co-repressors Dlx-1 and Dlx-2 and reduced expression of TβRII, as well as by RPPA analysis and western blotting, which confirmed reduced phosphorylation of Smad1/2/5/8. Interestingly, a similar association between TGF-β pathway inhibition and cell proliferation was noted previously for the stem/progenitor cell factor Sca-1 [14], as well as for the ability of embryonic stem cells to maintain pluripotency [28–30]. Thus, it appears that silencing of TGF-β signaling is also a major component of the conditional reprogramming process.

In summary, characterization of conditional reprogramming in HFKs identified enhanced growth factor signaling in concert with suppression of TGF-β and Notch signaling as factors in this process. These findings have the potential to expand the utility of this technique using the appropriate combinations of growth factors and inhibitors for the propagation and maintenance of primary tissues and their associated cell subpopulations as an adjunct to patient care.

## Supporting Information

S1 File Supporting Information. Fig. A, Optimization of cell culture with conditioned medium for siRNA screening of J2 cells. *I*, Colony assays of HFKs after 5 days using conditioned medium (CM) from J2 cells in the absence or presence of Y-27632 (Y). *II*, Colony assays of HFKs grown in medium supplemented with 100%, 50%, 25% or no (FY) CM from J2 cells in the presence of Y. Cell growth was measured by sulforhodamine staining at 560 nm. Growth was proportional to the dilution of CM at 200 cells/well. Fig. B, Uncropped and unadjusted western blots shown in Fig. 1D. Fig. C, Analysis of growth and apoptosis in conditionally reprogrammed HFKs. HFKs were maintained in culture for 2 days with Y-27632 (Y), J2 cells (J2) or J2 cells and Y (J2+Y). *I*, FACS with propidium iodide (*x-axis*) and a FITC conjugated annexin V antibody (*Y-axis*). There was no significant change in the percentage of double-positive apoptotic cells (*upper right quadrant*). *II*, Western analysis shows the absence of cleavage of Caspase 9 and Caspase 3, which indicate a lack of active apoptosis. The absence of cleavage of LC3B (I) to LC3B (II) indicate a lack of autophagy. Fig. D, Network analysis of genes (highlighted in blue) whose knockdown decreased HFK growth. Fig. E, Uncropped and unadjusted western blots shown in Fig. 4C. Table A, Antibodies used for immunofluorescence and western blotting. Table B, The siRNA library targeting factors secreted by J2 cells. Table C, Gene profile associated with the effect of Y-27632 and J2 cells on HFKs. Table D, Reverse-Phase Protein Array (RPPA) analysis.
(PDF)
