# Supplementary material for: Multifactorial Analysis of Conditional Reprogramming of Human Keratinocytes
Source: PLoS One. 2015 Feb 25;10(2):e0116755. doi: 10.1371/journal.pone.0116755 (PMC4340869; doi:10.1371/journal.pone.0116755)
Supplement: S1 File — Fig. A, Optimization of cell culture with conditioned medium for siRNA screening of J2 cells. I, Colony assays of HFKs after 5 days using conditioned medium (CM) from J2 cells in the absence or presence of Y-27632 (Y). II, Colony assays of HFKs grown in medium supplemented with 100%, 50%, 25% or no (FY) CM from J2 cells in the presence of Y. Cell growth was measured by sulforhodamine staining at 560 nm. Growth was proportional to the dilution of CM at 200 cells/well. Fig. B, Uncropped and unadjusted western blots shown in Fig. 1D. Fig. C, Analysis of growth and apoptosis in conditionally reprogrammed HFKs. HFKs were maintained in culture for 2 days with Y-27632 (Y), J2 cells (J2) or J2 cells and Y (J2+Y). I, FACS with propidium iodide (x-axis) and a FITC conjugated annexin V antibody (Y-axis). There was no significant change in the percentage of double-positive apoptotic cells (upper right quadrant). II, Western analysis shows the absence of cleavage of Caspase 9 and Caspase 3, which indicate a lack of active apoptosis. The absence of cleavage of LC3B (I) to LC3B (II) indicate a lack of autophagy. Fig. D, Network analysis of genes (highlighted in blue) whose knockdown decreased HFK growth. Fig. E, Uncropped and unadjusted western blots shown in Fig. 4C. Table A, Antibodies used for immunofluorescence and western blotting. Table B, The siRNA library targeting factors secreted by J2 cells. Table C, Gene profile associated with the effect of Y-27632 and J2 cells on HFKs. Table D, Reverse-Phase Protein Array (RPPA) analysis. (PDF) [file pone.0116755.s001.pdf]

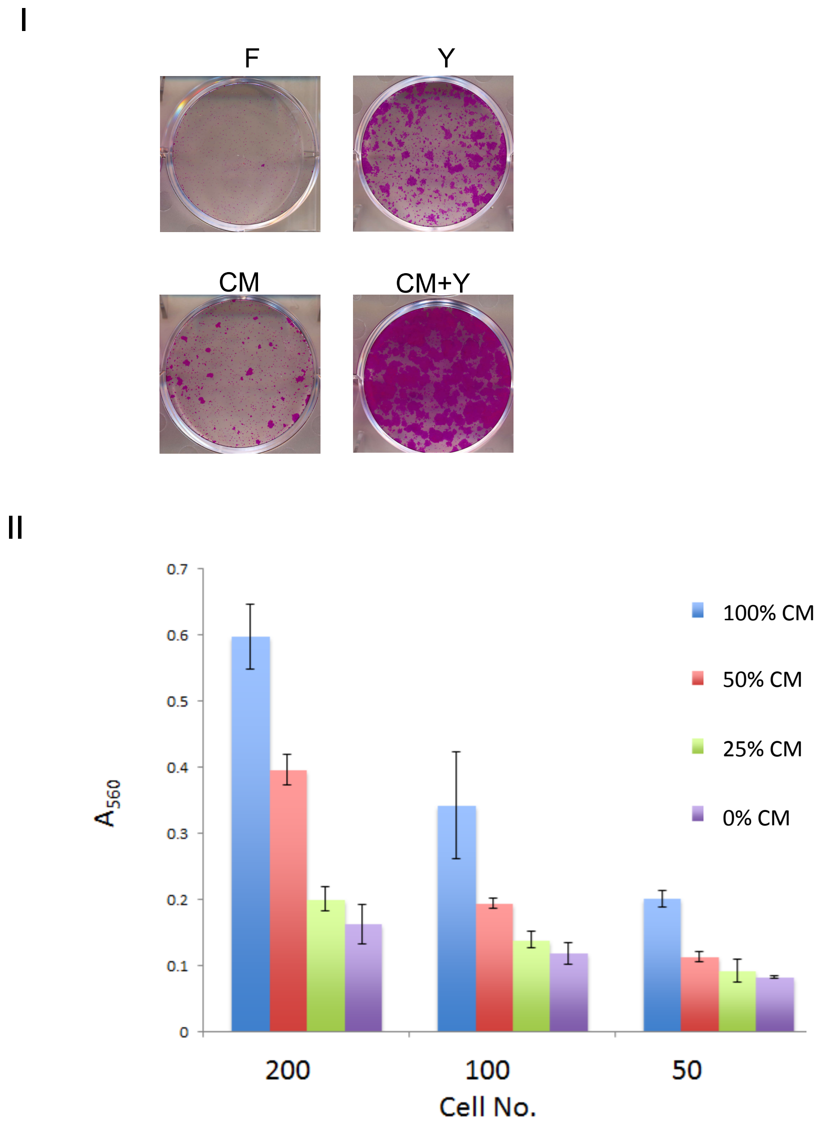

Fig. A. Optimization of cell culture with conditioned medium for siRNA screening of J2 cells. **I**, Colony assays of HFKs after 5 days using conditioned medium (CM) from J2 cells in the absence or presence of Y-27632 (Y). **II**, Colony assays of HFKs grown in medium supplemented with 100%, 50%, 25% or no (FY) CM from J2 cells in the presence of Y. Cell growth was measured by sulforhodamine staining at 560 nm. Growth was proportional to the dilution of CM at 200 cells/well.

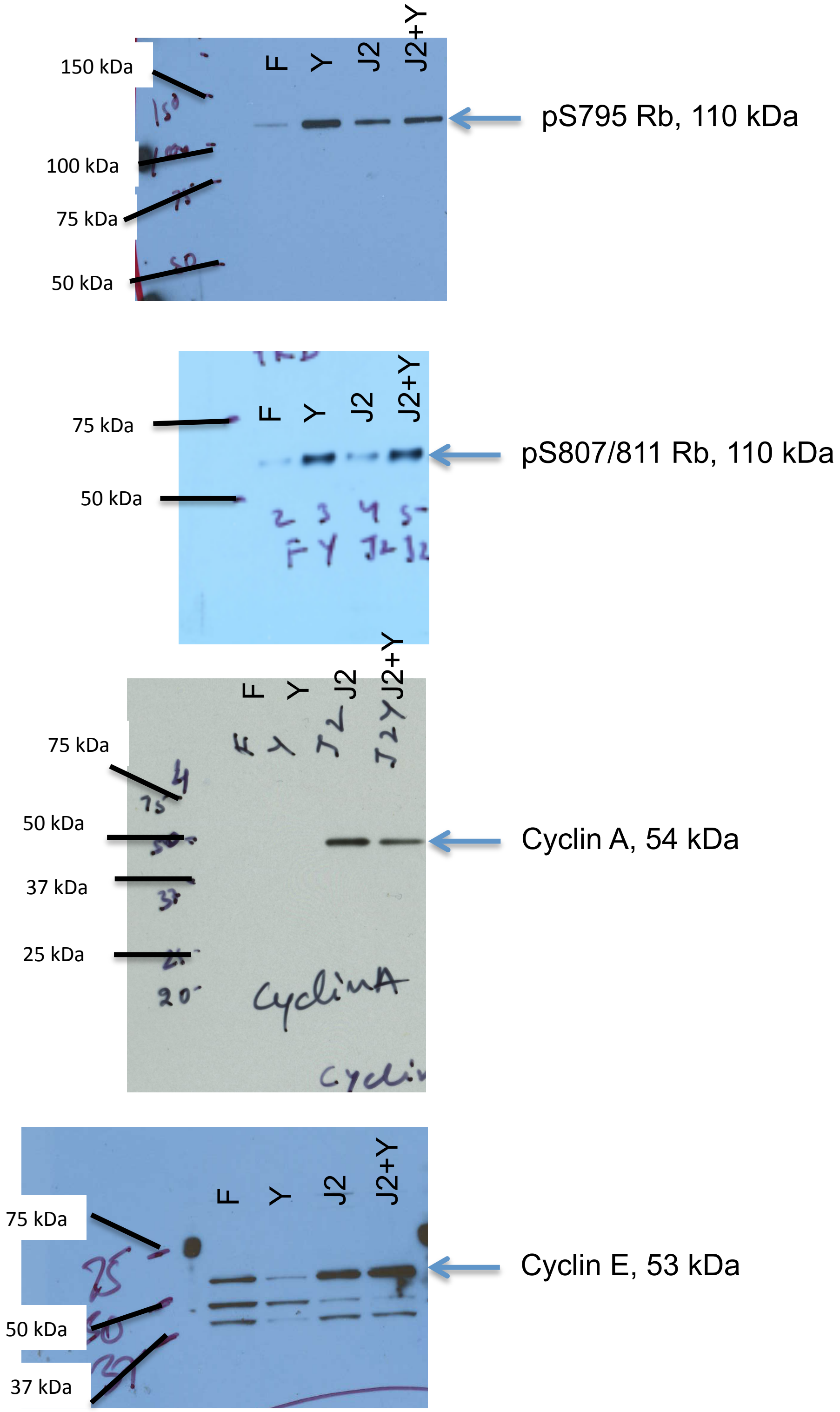

Fig. B: Uncropped and unadjusted blots presented in Figure 1D. Continued on next page..

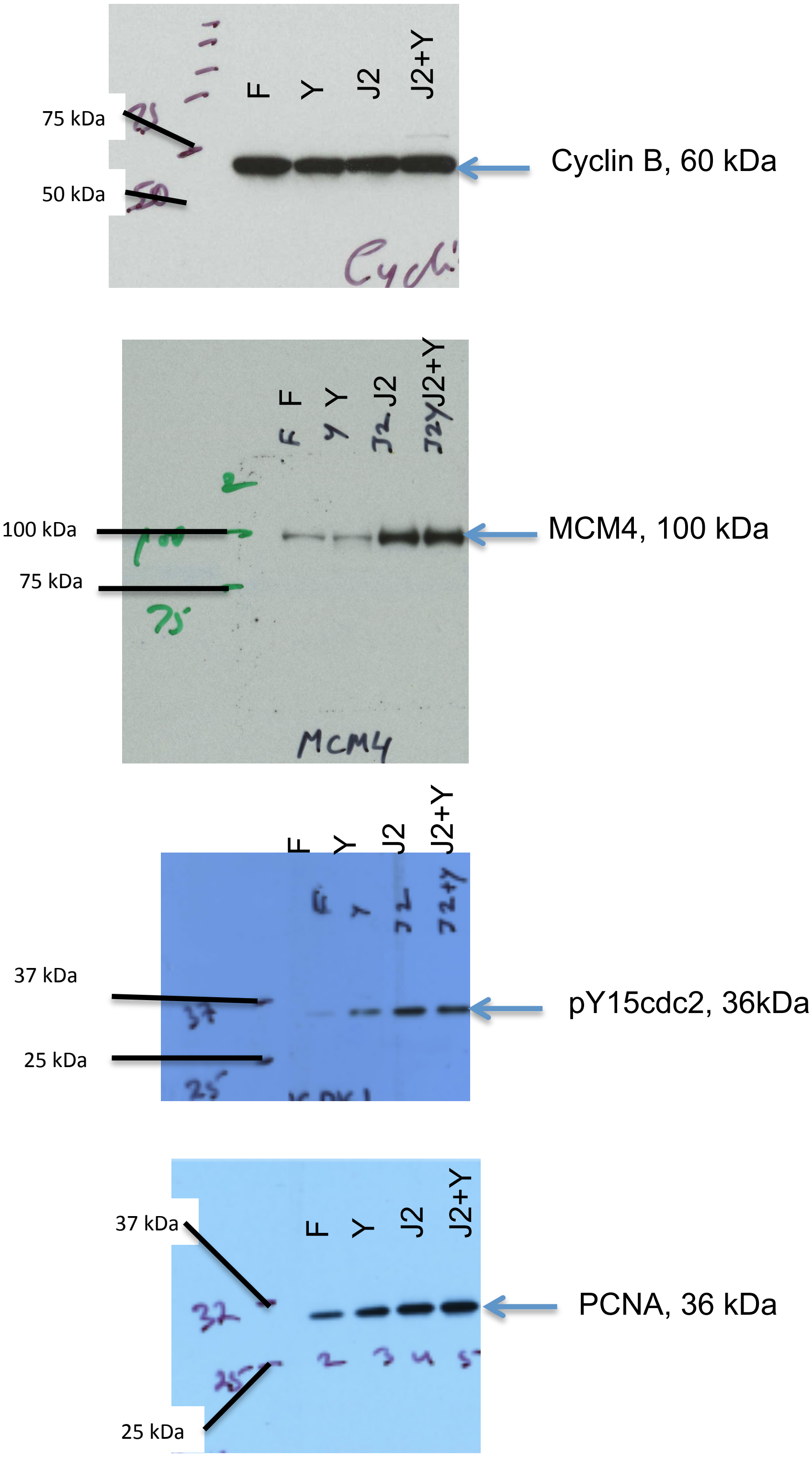

Fig. B: Uncropped and unadjusted blots presented in Figure 1D. Continued on next page..

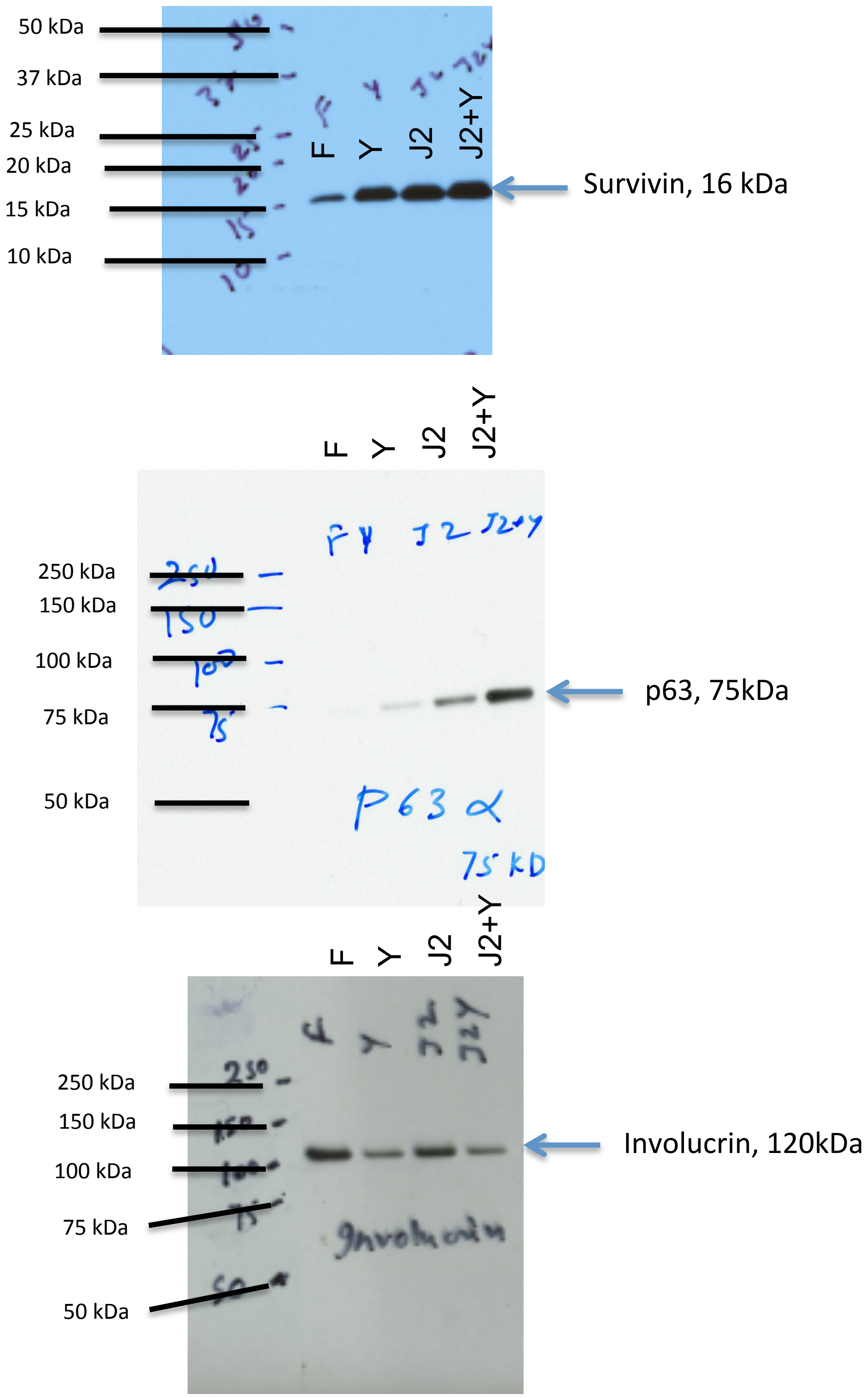

Fig. B: Uncropped and unadjusted blots presented in Figure 1D.

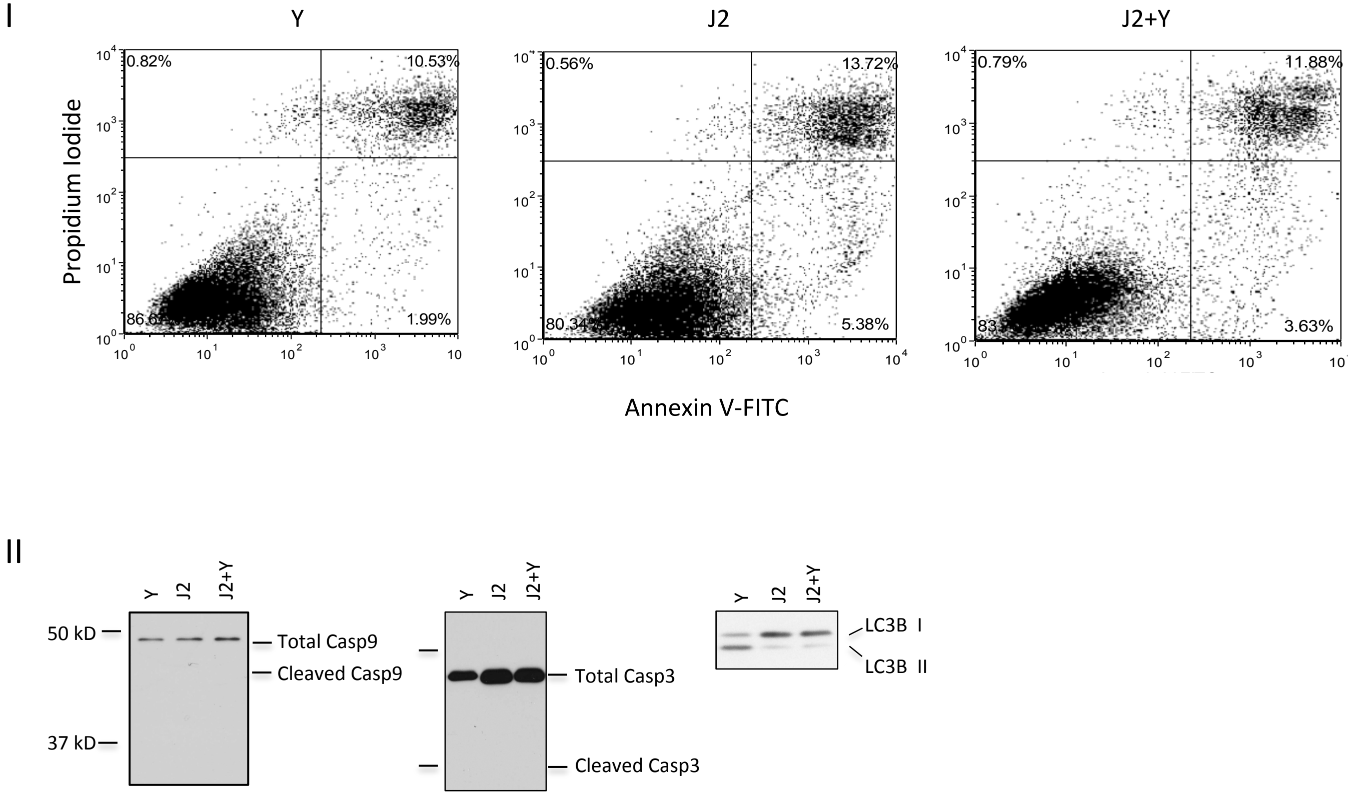

Fig. C. Analysis of growth and apoptosis in conditionally reprogrammed HFKs. HFKs were maintained in culture for 2 days with Y-27632 (Y), J2 cells (J2) or J2 cells and Y (J2+Y). **I**, FACS with propidium iodide (*x-axis*) and a FITC conjugated annexin V antibody (*Y-axis*). There was no significant change in the percentage of double-positive apoptotic cells (*upper right quadrant*). **II**, Western analysis shows the absence of cleavage of Caspase 9 and Caspase 3, which indicate a lack of active apoptosis. The absence of cleavage of LC3B (I) to LC3B (II) indicate a lack of autophagy.

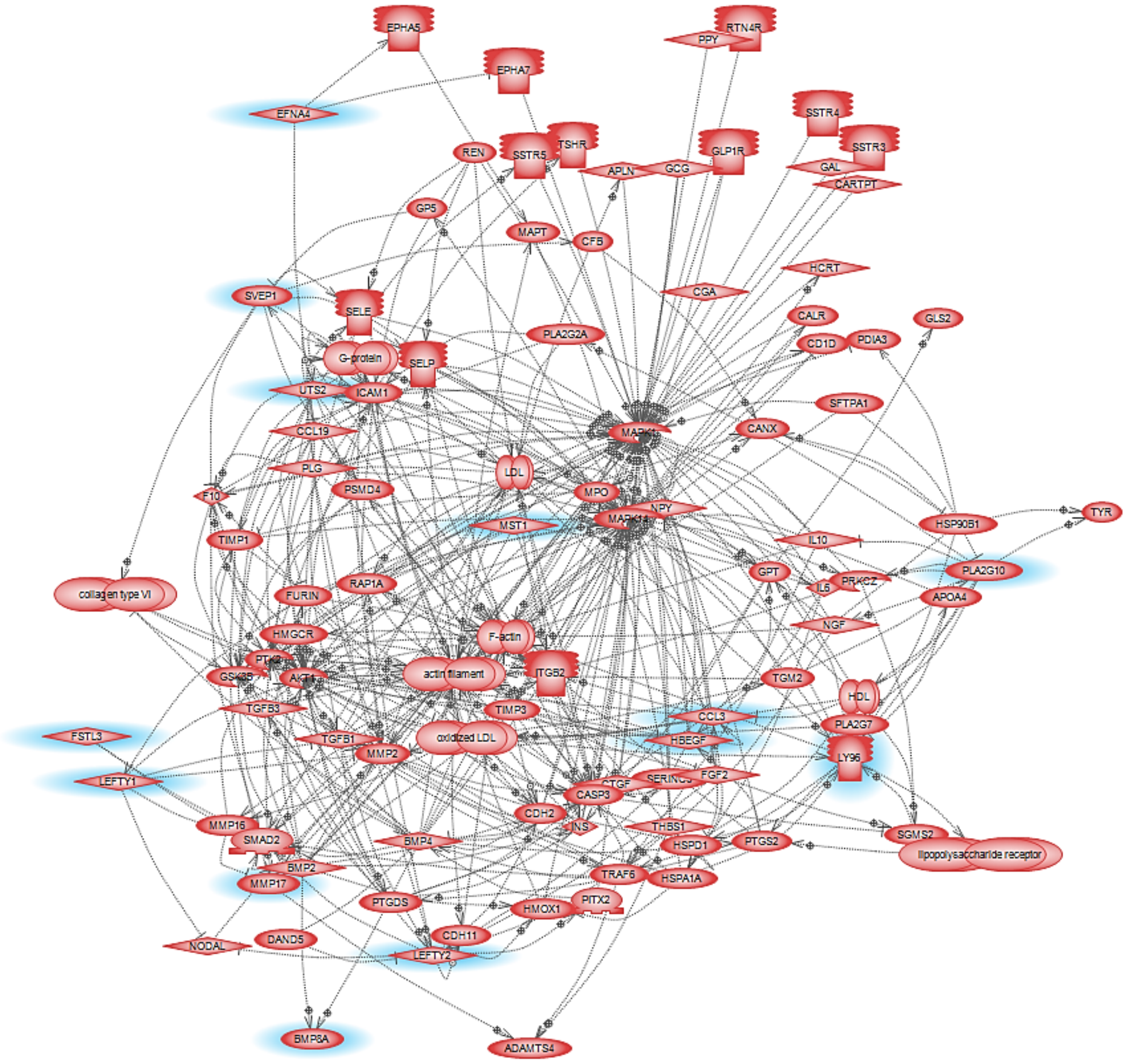

Fig. D. Network analysis of genes (highlighted in blue) whose knockdown decreased HFK growth.

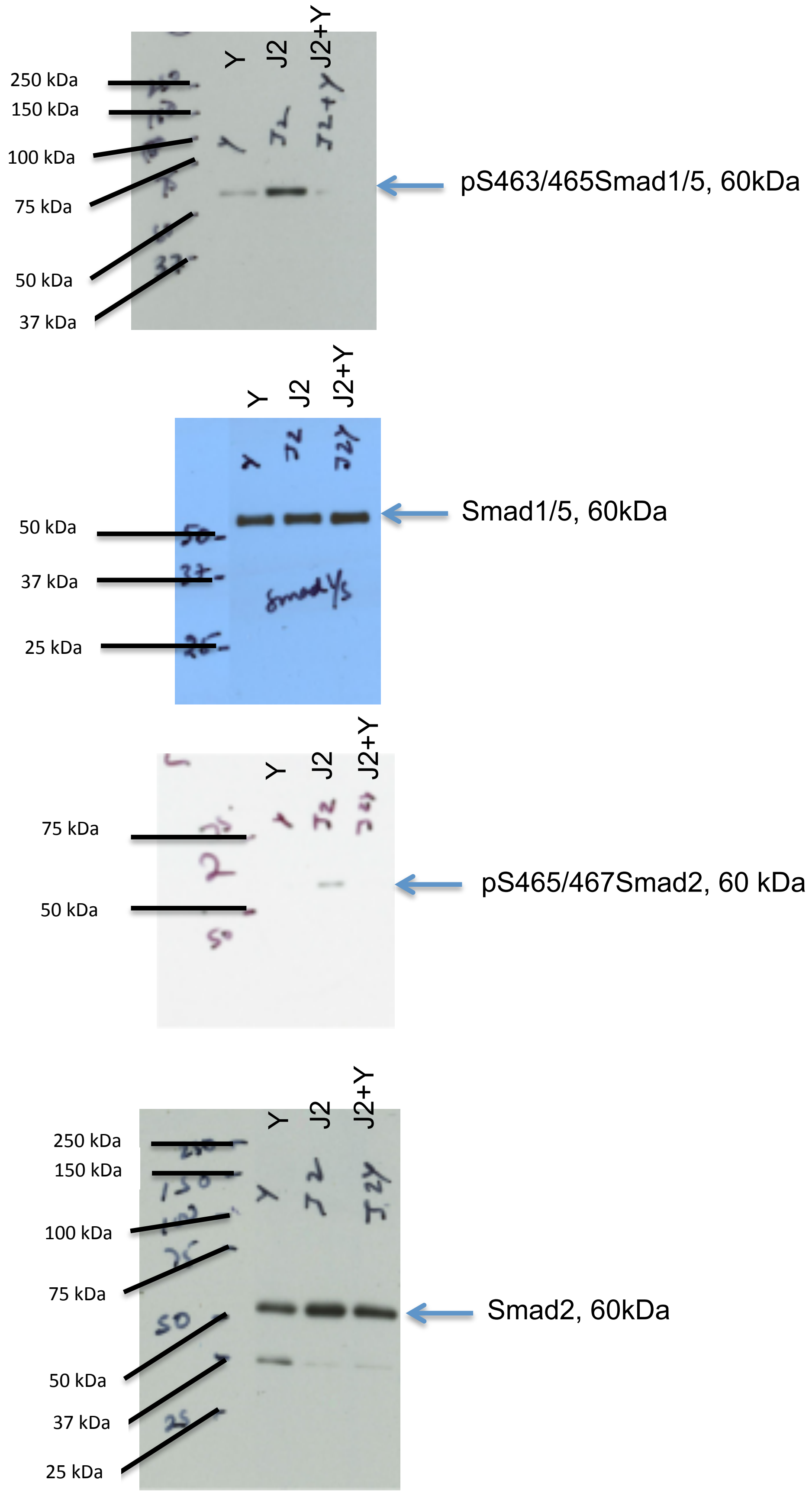

Fig. E: Uncropped and unadjusted blots presented in Figure 4C. Continued on next page..

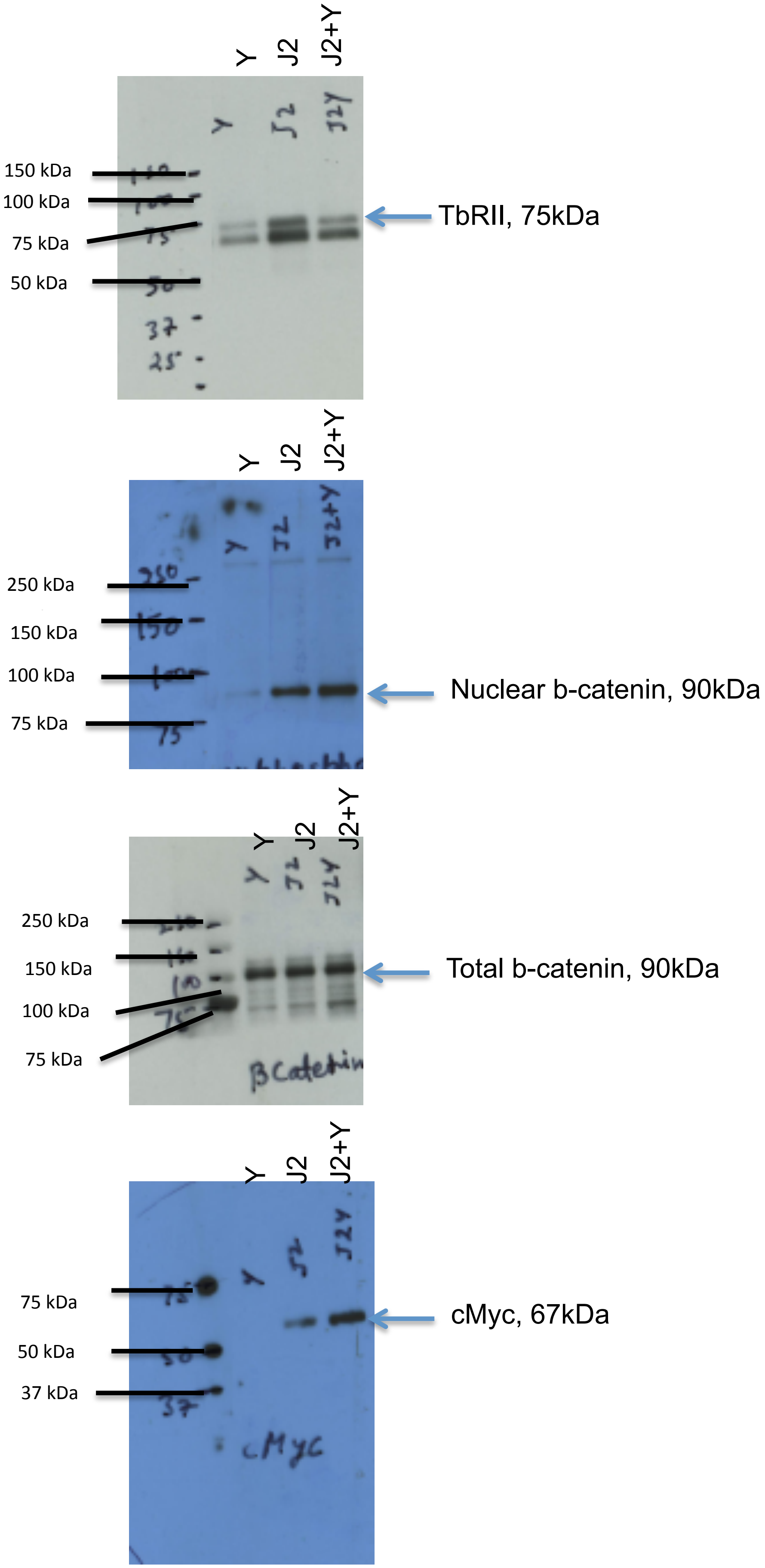

Fig. E: Uncropped and unadjusted blots presented in Figure 4C. Continued on next page..

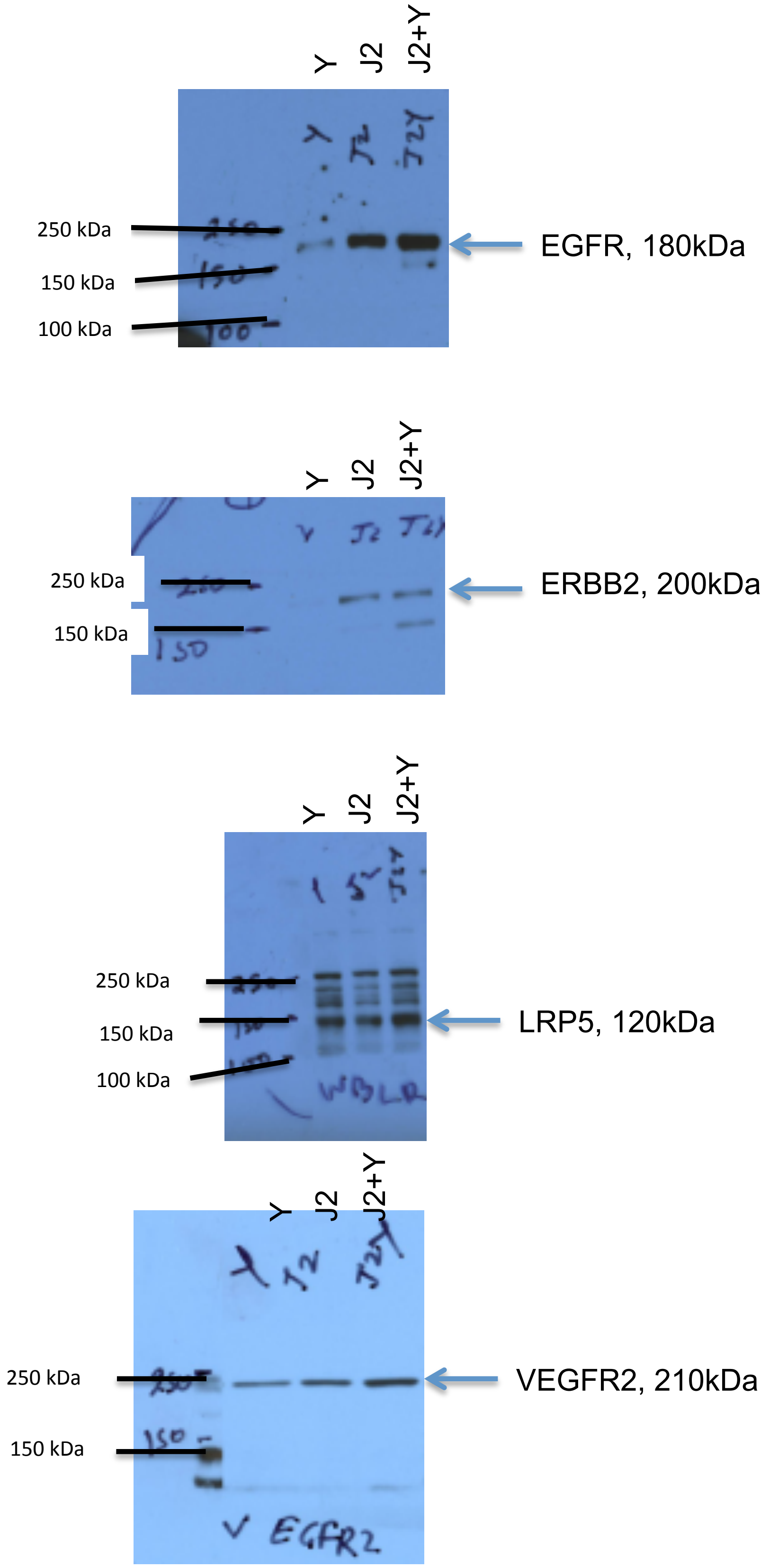

Fig. E: Uncropped and unadjusted blots presented in Figure 4C. Continued on next page..

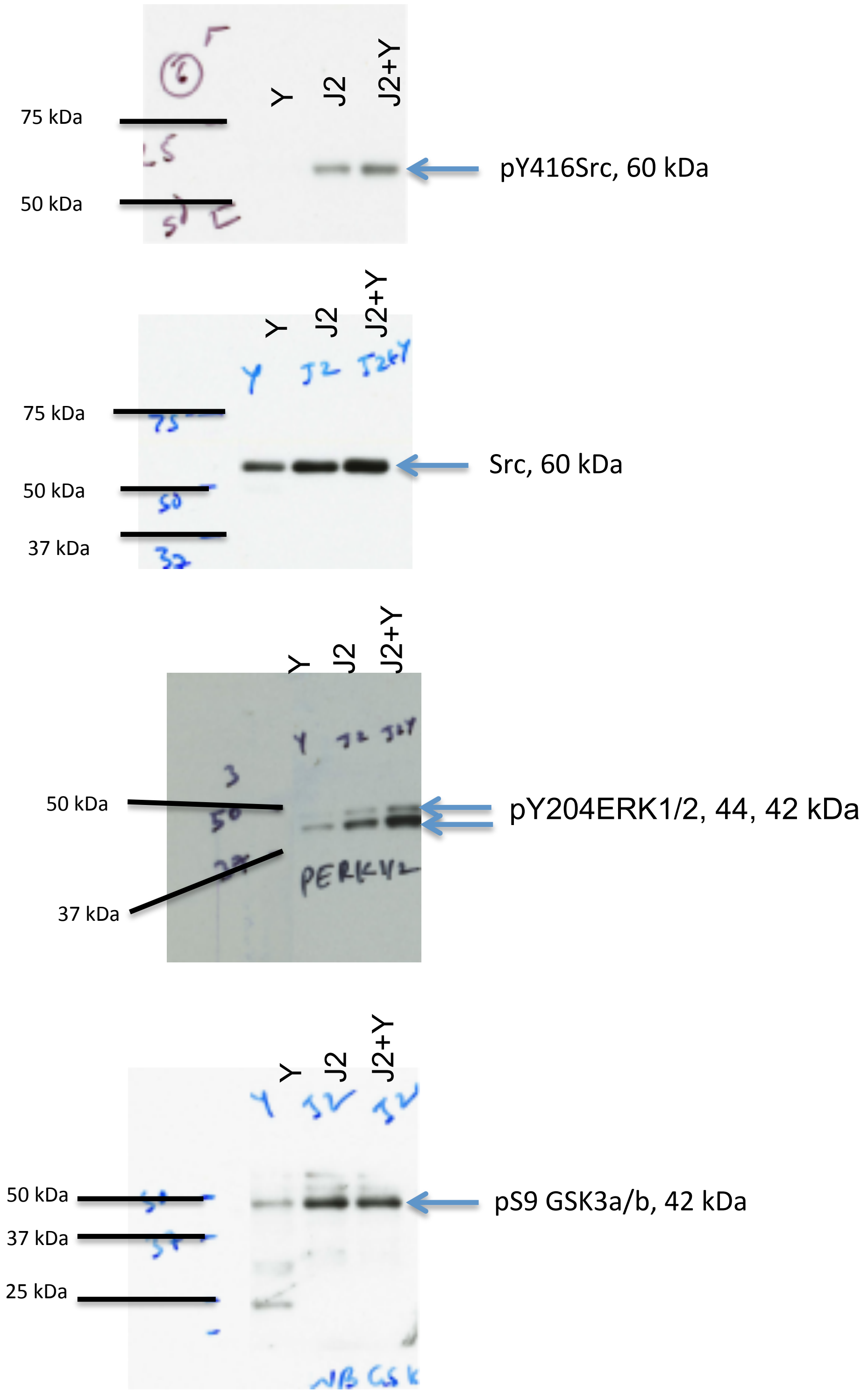

Fig. E: Uncropped and unadjusted blots presented in Figure 4C. Continued on next page..

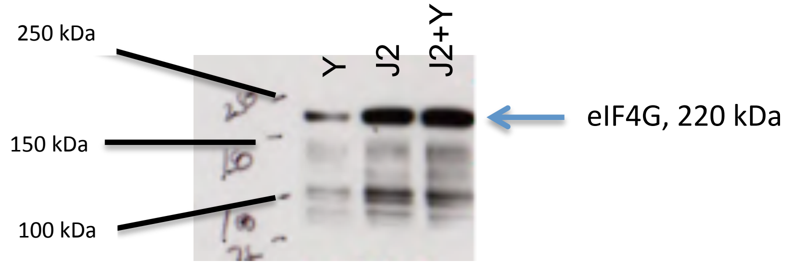

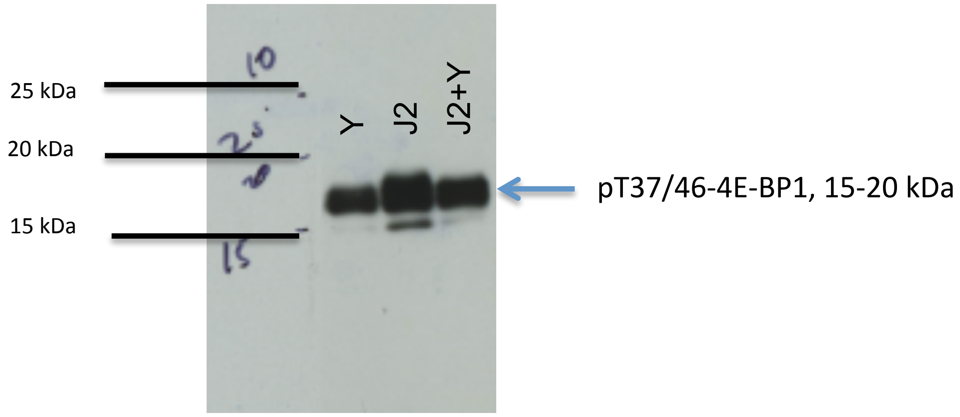

Fig. E: Uncropped and unadjusted blots presented in Figure 4C.

Table A. Antibodies used for immunofluorescence and western blotting.

Name	Company	Catalog number
F-Actin	Novus Biologicals	NB100-64792
E Cadherin	Cell Signaling	5296
pS463/465Smad1/5	Cell Signaling	9516
Smad5	Cell Signaling	12534
pS465/467Smad2	Cell Signaling	3108
Smad2	Cell Signaling	5339
TGFbRII	Santa Cruz	sc-400
free b-Catenin	Cell Signaling	4270
total b-Catenin	Cell Signaling	2698
c-Myc	Cell Signaling	5605p
pY416Src	Cell Signaling	2101s
Src	Cell Signaling	2108s
EGFR	BD Biosciences	610017
ERBB2	Cell Signaling	4290
LRP5	AbCam	ab38311
pY204ERK1/2	Santa Cruz	sc-7383
VEGFR2	Cell Signaling	2479
pS9/21GSK3a/b	Cell Signaling	8566
pS807/811Rb	Cell Signaling	9308
pS795Rb	Cell Signaling	9301
pY15cdc2	Cell Signaling	9111
Cyclin A	Santa Cruz	sc-751
Cyclin E	Santa Cruz	sc-247
Cyclin B	Santa Cruz	sc-245
MCM4	Santa Cruz	sc-28317
PCNA	Santa Cruz	sc-7907
Survivin	Santa Cruz	sc-10811
p63alpha	Santa Cruz	sc-8344
Involucrin	Santa Cruz	sc-28557
eIF4G	Cell Signaling	2498
pT37/46-4E-BP1	Cell Signaling	9459
BrdU	Invitrogen	B35128
Caspase 3	Cell Signaling	9662
Caspase 9	Cell Signaling	9502
LC3B	Cell Signaling	2775

Table B. siRNA library targeting factors secreted by J2 cells.

Description	Entrez Gene ID	Gene Symbol	Gene Description	mRNA Accessior	siRNA Target Sequence	Qiagen ID	Qiagen Product
receptors	13052	Cxadr	coxsackie virus and adenovirus receptor	NM_001025192 N	TGGAAATGACTTCAAGCTAAA	SI02736377	Mm_Cxadr_8
receptors	13052	Cxadr	coxsackie virus and adenovirus receptor	NM_001025192 N	TTGGAAATGACTTCAAGCTAA	SI02711954	Mm_Cxadr_7
secreted	73381	Cmtm2a	CKLF-like MARVEL transmembrane domain containing 2A	NM_027022	TATAAATGTTATGAAATATAA	SI00951944	Mm_Cklfsf2a_4
secreted	73381	Cmtm2a	CKLF-like MARVEL transmembrane domain containing 2A	NM_027022	CTGACCTTTCTCTTAACTATA	SI00951937	Mm_Cklfsf2a_3
secreted	64654	Fgf23	fibroblast growth factor 23	NM_022657	TTCGCCAATGATTCTGTGATA	SI02742964	Mm_Fgf23_6
secreted	64654	Fgf23	fibroblast growth factor 23	NM_022657	ATCCATAGGGATGGTCATGTA	SI02674469	Mm_Fgf23_5
secreted	170721	Papln	papilin, proteoglycan-like sulfated glycoprotein	NM_130887	CAGGAATGGCCTGCCAATACA	SI01369200	Mm_Papln_4
secreted	170721	Papln	papilin, proteoglycan-like sulfated glycoprotein	NM_130887	CACCGTGGAACAGGAGCAGAA	SI01369193	Mm_Papln_3
receptors	18596	Pdgfrb	platelet derived growth factor receptor, beta polypeptide	NM_001146268 N	AAGCCTGACGTTGCTGATGAA	SI01373904	Mm_Pdgfrb_4
receptors	18596	Pdgfrb	platelet derived growth factor receptor, beta polypeptide	NM_001146268 N	CACCATGAAAGTGGCTGTCAA	SI01373897	Mm_Pdgfrb_3
secreted	16854	Lgals3	lectin, galactose binding, soluble 3	NM_001145953 N	CTGCTCGTGACTGCTAGGCAA	SI01089480	Mm_Lgals3_4
secreted	16854	Lgals3	lectin, galactose binding, soluble 3	NM_001145953 N	CACAATCATGGGCACAGTGAA	SI01089473	Mm_Lgals3_3
secreted	15245	Hhip	Hedgehog-interacting protein	NM_020259	CAGATGGAAGACTACGAGAAA	SI01065008	Mm_Hhip_4
secreted	15245	Hhip	Hedgehog-interacting protein	NM_020259	TCGGCTCTTCATTCTAGAGAA	SI01065001	Mm_Hhip_3
secreted	20720	Serpine2	serine (or cysteine) peptidase inhibitor, clade E, member 2	NM_009255	CTCCACGGTGATGCGATATAA	SI02710701	Mm_Serpine2_7
secreted	20720	Serpine2	serine (or cysteine) peptidase inhibitor, clade E, member 2	NM_009255	CTGATCAGTTATCTAGGTTAA	SI02688735	Mm_Serpine2_6
receptors	52118	Pvr	poliovirus receptor	NM_027514	TAGACTGAACATGGAGCCAAA	SI02744399	Mm_D7Ertd458e_8
receptors	52118	Pvr	poliovirus receptor	NM_027514	ATGAAGTAAAGTATATATTTA	SI02719248	Mm_D7Ertd458e_7
secreted	18208	Ntn1	netrin 1	NM_008744	CGCGCCTATCACCAAACAGAA	SI00178983	Mm_Ntn1_4
secreted	18208	Ntn1	netrin 1	NM_008744	AACATGGAGCTCTATAAGCTA	SI02709875	Mm_Ntn1_5
secreted	14423	Galnt1	UDP-N-acetyl-alpha-D-galactosamine:polypeptide N-acetylgalactosaminyl	NM_001160404 N	CAGGAGGGACAGGGCAGATTA	SI01008812	Mm_Galnt1_4
secreted	14423	Galnt1	UDP-N-acetyl-alpha-D-galactosamine:polypeptide N-acetylgalactosaminyl	NM_001160404 N	AACCAGGTTCAGAATCATGAA	SI01008805	Mm_Galnt1_3
secreted	67305	Gpx7	glutathione peroxidase 7	NM_024198	TTGGATGATTTGAATCCAAGA	SI01057896	Mm_Gpx7_4
secreted	67305	Gpx7	glutathione peroxidase 7	NM_024198	CTCGGTTTCCCTGGTGGTGAA	SI01057889	Mm_Gpx7_3
secreted	237360	Adamts14	a disintegrin-like and metallopeptidase (reprolysin type) with thrombospc	NM_001081127	CACCTACGAATGGGCTCTCAA	SI04391982	Mm_Adamts14_1
secreted	237360	Adamts14	a disintegrin-like and metallopeptidase (reprolysin type) with thrombospc	NM_001081127	CCGAGGTAGCCTGGCCTACAA	SI01103368	Mm_LOC237360_4
secreted	21892	Tll1	tolloid-like	NM_009390	CAGAGTGTGGTGGTCGATTGA	SI04405296	Mm_Tll1_5
secreted	21892	Tll1	tolloid-like	NM_009390	ACGAAGCTCAATGGCACCATA	SI04449522	Mm_Tll1_6
receptors	14275	Folr1	folate receptor 1 (adult)	NM_008034	AACTATGACATCGGAATGCAA	SI02732569	Mm_Folr1_7
receptors	14275	Folr1	folate receptor 1 (adult)	NM_008034	CACAGGATTCAGGCCACTAAA	SI02686642	Mm_Folr1_6
secreted	18256	Oc90	otoconin 90	NM_010953	CCCACAGATACCAGTCAGATA	SI01334452	Mm_Oc90_4
secreted	18256	Oc90	otoconin 90	NM_010953	CACAATGATTATGCTGCTCAT	SI01334445	Mm_Oc90_3
secreted	242022	Frem2	Fras1 related extracellular matrix protein 2	NM_172862	AAGGGTGAGAGTGATATTTAT	SI02773176	Mm_Frem2_4
secreted	242022	Frem2	Fras1 related extracellular matrix protein 2	NM_172862	TGGGAGAAACTTCATTTATAA	SI02765126	Mm_Frem2_3
secreted	12331	Cap1	CAP, adenylate cyclase-associated protein 1 (yeast)	NM_007598	CAGCACTGTTTGCACAGATTA	SI02731785	Mm_Cap1_7
secreted	12331	Cap1	CAP, adenylate cyclase-associated protein 1 (yeast)	NM_007598	CAACACAACATTGCAAATCAA	SI02707761	Mm_Cap1_6
secreted	15191	Hdgf	hepatoma-derived growth factor	NM_008231	CCCACTAAGAATACAGGGAAA	SI01063944	Mm_Hdgf_4
secreted	15191	Hdgf	hepatoma-derived growth factor	NM_008231	AGGAGTGGAGGTGAAAGAATA	SI01063937	Mm_Hdgf_3
secreted	19063	Ppt1	palmitoyl-protein thioesterase 1	NM_008917	ATGGTGTTGGATTCAAGTTTA	SI02734410	Mm_Ppt1_7
secreted	19063	Ppt1	palmitoyl-protein thioesterase 1	NM_008917	CAGATCAGTAATTGTGCCTAA	SI02710106	Mm_Ppt1_6
receptors	14062	F2r	coagulation factor II (thrombin) receptor	NM_010169	TCCGGTGTTGGCTGATCTCAA	SI04918697	Mm_F2r_10
receptors	14062	F2r	coagulation factor II (thrombin) receptor	NM_010169	AGGGTAGGGCAGTCTACTTAA	SI04918690	Mm_F2r_9
secreted	65960	Twsg1	twisted gastrulation homolog 1 (Drosophila)	NM_023053	ATGAGTAAATATTACAGTTTA	SI01459136	Mm_Twsg1_4
secreted	65960	Twsg1	twisted gastrulation homolog 1 (Drosophila)	NM_023053	CTGTAGCTTATTGGTAATTAA	SI01459129	Mm_Twsg1_3
secreted	23876	Fbln5	fibulin 5	NM_011812	CAGCTTAGTGTCTTCTTTAAA	SI00999684	Mm_Fbln5_4
secreted	23876	Fbln5	fibulin 5	NM_011812	CAGACTTGCTACAATCTACAA	SI00999677	Mm_Fbln5_3
secreted	12317	Calr	calreticulin	NM_007591	CGGGACAATCTTTGACAATTT	SI04449004	Mm_Calr_10
secreted	12317	Calr	calreticulin	NM_007591	AAGAAGGTTCATGTCATCTTT	SI04433828	Mm_Calr_9
secreted	30052	Pcsk1n	proprotein convertase subtilisin/kexin type 1 inhibitor	NM_013892	TAGGGCGGATCCTCACCGGAA	SI01372952	Mm_Pcsk1n_4
secreted	30052	Pcsk1n	proprotein convertase subtilisin/kexin type 1 inhibitor	NM_013892	ACGCGTCAAACGCCTGGAGAA	SI01372945	Mm_Pcsk1n_3
receptors	17082	Il1rl1	interleukin 1 receptor-like 1	NM_001025602 N	CAGCACCTGTTCAGTGGTTTA	SI02713067	Mm_Il1rl1_7
receptors	17082	Il1rl1	interleukin 1 receptor-like 1	NM_001025602 N	AACGTGACTCATGATGATGAA	SI02737616	Mm_Il1rl1_8
secreted	72113	Adck1	aarF domain containing kinase 1	NM_028105	ATCCTGAAGACTAATGATCTA	SI00889980	Mm_Adck1_4
secreted	72113	Adck1	aarF domain containing kinase 1	NM_028105	TATATTGTTTATTGTAATAAA	SI00889973	Mm_Adck1_3
secreted	57890	Il17re	interleukin 17 receptor E	NM_001034029 N	TCCCTGAAGCAGGGTCACAAA	SI04944541	Mm_Il17re_7
secreted	57890	Il17re	interleukin 17 receptor E	NM_001034029 N	AGGAGTCAGAAGGATGGTATA	SI04944534	Mm_Il17re_6
secreted	12667	Chrd	chordin	NM_009893	CCGCAGGAGCGCAGCAATCTA	SI00950796	Mm_Chrd_4
secreted	12667	Chrd	chordin	NM_009893	CTGGCTTATTGGGATTCTTTA	SI00950789	Mm_Chrd_3
secreted	26970	Pla2g2e	phospholipase A2, group IIE	NM_012044	TACCCTCTTCTGAATAAGAGA	SI02715055	Mm_Pla2g2e_4
secreted	26970	Pla2g2e	phospholipase A2, group IIE	NM_012044	AGCGCTCACAAGATCCACTAA	SI02692445	Mm_Pla2g2e_3
receptors	16194	Il6ra	interleukin 6 receptor, alpha	NM_010559	CCCGGCAGGAATCCTCTGGAA	SI02737336	Mm_Il6ra_6
receptors	16194	Il6ra	interleukin 6 receptor, alpha	NM_010559	CAGTACGAAAGTTCTACAGAA	SI02690478	Mm_Il6ra_5
secreted	17180	Matn1	matrilin 1, cartilage matrix protein	NM_010769	GAGGGTGGGCTTGGTCAACTA	SI01301636	Mm_Matn1_4
secreted	17180	Matn1	matrilin 1, cartilage matrix protein	NM_010769	AACCAGATTGTGGACACGTTA	SI01301629	Mm_Matn1_3
secreted	65945	Clstn1	calsyntenin 1	NM_023051	CTGGACTGTGAACTACAGAAA	SI00953484	Mm_Clstn1_4
secreted	65945	Clstn1	calsyntenin 1	NM_023051	CCCGTCTGAGGAGAAGAAATA	SI00953477	Mm_Clstn1_3
secreted	18787	Serpine1	serine (or cysteine) peptidase inhibitor, clade E, member 1	NM_008871	ATGCTTGGCAACCCACGTTAA	SI04777759	Mm_Serpine1_6
secreted	18787	Serpine1	serine (or cysteine) peptidase inhibitor, clade E, member 1	NM_008871	ACGGAGATGGTTATAGACCGA	SI04777752	Mm_Serpine1_5
secreted	20256	Clec11a	C-type lectin domain family 11, member a	NM_009131	AATGAGGATAATCTTGCTGAA	SI02710470	Mm_Scgf_6
secreted	20256	Clec11a	C-type lectin domain family 11, member a	NM_009131	TACATGTAGGCCGGAGCATAA	SI02688560	Mm_Scgf_5
receptors	20350	Sema3f	sema domain, immunoglobulin domain (Ig), short basic domain, secreted,	NM_011349	TAGACCCGTGTTGGTCAGCAA	SI02714033	Mm_Sema3f_6
receptors	20350	Sema3f	sema domain, immunoglobulin domain (Ig), short basic domain, secreted,	NM_011349	AAGAGCTGTCAAGATCTACAA	SI02671326	Mm_Sema3f_5
secreted	20347	Sema3b	sema domain, immunoglobulin domain (Ig), short basic domain, secreted,	NM_001042779 N	CAGCATGCAAATCTCCTCTAA	SI02710547	Mm_Sema3b_7
secreted	20347	Sema3b	sema domain, immunoglobulin domain (Ig), short basic domain, secreted,	NM_001042779 N	AAAGGGTAGCTTATTTATTAA	SI02668757	Mm_Sema3b_5
secreted	14170	Fgf15	fibroblast growth factor 15	NM_008003	AAGGTATGAAGTCAAATGATA	SI02708440	Mm_Fgf15_5
secreted	14170	Fgf15	fibroblast growth factor 15	NM_008003	AAGGAGTTGGTTTGTATCAAA	SI00170982	Mm_Fgf15_4
secreted	321007	Serac1	serine active site containing 1	NM_001111017 N	CACAGTTGACATAGAAGATCA	SI01414560	Mm_Serac1_4
secreted	321007	Serac1	serine active site containing 1	NM_001111017 N	ATGAGTGTCTCCAGTACTTTA	SI01414553	Mm_Serac1_3
secreted	18606	Enpp2	ectonucleotide pyrophosphatase/phosphodiesterase 2	NM_001136077 N	CTGCAGTGCTTTATCGGACTA	SI04930149	Mm_Enpp2_11
secreted	18606	Enpp2	ectonucleotide pyrophosphatase/phosphodiesterase 2	NM_001136077 N	ATGGAATCGTTGGCAATTCAA	SI04930142	Mm_Enpp2_10
receptors	14174	Fgf3	fibroblast growth factor 3	NM_008007	CAAGCTCTACTGCGCTACCAA	SI01002484	Mm_Fgf3_4
receptors	14174	Fgf3	fibroblast growth factor 3	NM_008007	TCCAGTGGGCCAGGAGCTCAA	SI01002477	Mm_Fgf3_3
secreted	56708	Clcf1	cardiotrophin-like cytokine factor 1	NM_019952	CCGGCTTAAGAAGAAGATGCA	SI02694349	Mm_MGI:1930088_6
secreted	56708	Clcf1	cardiotrophin-like cytokine factor 1	NM_019952	CACATGGTTTCTGACCTCTGA	SI02673923	Mm_MGI:1930088_5
receptors	16160	Il12b	interleukin 12b	NM_008352	AGAAGTGATATTGATAAGAAA	SI02733157	Mm_Il12b_7
receptors	16160	Il12b	interleukin 12b	NM_008352	TTGAAATATTTAAGTAATTTA	SI02667483	Mm_Il12b_5
secreted	56066	Cxcl11	chemokine (C-X-C motif) ligand 11	NM_019494	CCCGGGATGAAAGCCGTCAAA	SI03975419	Mm_LOC630447_1
secreted	56066	Cxcl11	chemokine (C-X-C motif) ligand 11	NM_019494	AAGAATTGTGTTTCTCTAGTA	SI02741529	Mm_Cxcl11_8
receptors	12305	Ddr1	discoidin domain receptor family, member 1	NM_007584 NM_	CCGCCGCTGGATGGACTGGAA	SI00975828	Mm_Ddr1_4
receptors	12305	Ddr1	discoidin domain receptor family, member 1	NM_007584 NM_	AAGGAGGTAAAGATCATGTCA	SI00975821	Mm_Ddr1_3
secreted	80883	Ntng1	netrin G1	NM_001163348 N	AGGCGGCGATTTGGTTCCTAA	SI05182625	Mm_Ntng1_6
secreted	80883	Ntng1	netrin G1	NM_001163348 N	CTGGACCTCGGCTACGAAATA	SI05182618	Mm_Ntng1_5
secreted	13052	Cxadr	coxsackie virus and adenovirus receptor	NM_001025192 N	TGGAAATGACTTCAAGCTAAA	SI02736377	Mm_Cxadr_8
secreted	13052	Cxadr	coxsackie virus and adenovirus receptor	NM_001025192 N	TTGGAAATGACTTCAAGCTAA	SI02711954	Mm_Cxadr_7
secreted	12162	Bmp7	bone morphogenetic protein 7	NM_007557	AGCCGAATTCAGGATCTATAA	SI02707670	Mm_Bmp7_5
secreted	12162	Bmp7	bone morphogenetic protein 7	NM_007557	CTAGTGGAACATGACAAAGAA	SI00167216	Mm_Bmp7_4
receptors	21687	Tek	endothelial-specific receptor tyrosine kinase	NM_013690	CCGGTATAAGGTTCAGGGCAA	SI04418022	Mm_Tek_6
receptors	21687	Tek	endothelial-specific receptor tyrosine kinase	NM_013690	ACGGACCAGACTGTAAGCTCA	SI04418015	Mm_Tek_5
secreted	240913	Adamts4	a disintegrin-like and metallopeptidase (reprolysin type) with thrombospc	NM_172845	TTGTATTTATTTGGTATTTAT	SI02747668	Mm_Adamts4_7
secreted	240913	Adamts4	a disintegrin-like and metallopeptidase (reprolysin type) with thrombospc	NM_172845	AATGATGTTAATAAAGAACTA	SI02698619	Mm_Adamts4_6

secreted	330260	Pon2	paraoxonase 2	NM_183308	TGCAGGACTACTGATAGATAA	SI04929337	Mm_Pon2_9
secreted	330260	Pon2	paraoxonase 2	NM_183308	ACCATCAGTACTGACCTAGTA	SI04929344	Mm_Pon2_10
secreted	14171	Fgf17	fibroblast growth factor 17	NM_008004	CCCAGGGTGGGCACCATTCTA	SI01002316	Mm_Fgf17_4
secreted	14171	Fgf17	fibroblast growth factor 17	NM_008004	CTGAACCTCTCTGAATCTGAA	SI01002309	Mm_Fgf17_3
secreted	56221	Ccl24	chemokine (C-C motif) ligand 24	NM_019577	CTGGGATGAGGTCTAAGTTCA	SI02741620	Mm_Ccl24_4
secreted	56221	Ccl24	chemokine (C-C motif) ligand 24	NM_019577	TGGGTTCAGAGGCACATACAA	SI02716833	Mm_Ccl24_3
secreted	246228	Vwa1	von Willebrand factor A domain containing 1	NM_147776	TTGGACGTACAGGGTTTATTA	SI00849940	Mm_4932416A11Rik_4
secreted	246228	Vwa1	von Willebrand factor A domain containing 1	NM_147776	CAGGAGCTAAGTATTGAGAGA	SI00849933	Mm_4932416A11Rik_3
secreted	246316	Lgi2	leucine-rich repeat LGI family, member 2	NM_144945	CAGGAGAAGAAACTCAACGAA	SI01089676	Mm_Lgi2_4
secreted	246316	Lgi2	leucine-rich repeat LGI family, member 2	NM_144945	TACCAGTGGGATAAAGAGAAA	SI01089669	Mm_Lgi2_3
secreted	66116	Cml1	camello-like 1	NM_023160	AGCAGGTATTTGTACAATTCA	SI05159567	Mm_Cml1_5
secreted	66116	Cml1	camello-like 1	NM_023160	AAGGTGTGCAAGGCAGGTATA	SI00953820	Mm_Cml1_4
receptors	15980	Ifngr2	interferon gamma receptor 2	NM_008338	ACAGAGTATATTGTTTACAAA	SI02709000	Mm_Ifngr2_7
receptors	15980	Ifngr2	interferon gamma receptor 2	NM_008338	TACGGACATCACAGAGACAAA	SI02687125	Mm_Ifngr2_6
secreted	12159	Bmp4	bone morphogenetic protein 4	NM_007554	CACCAGACTAGTCCATCACAA	SI00929936	Mm_Bmp4_4
secreted	12159	Bmp4	bone morphogenetic protein 4	NM_007554	AACAGGGCTTCCACCGTATAA	SI00929929	Mm_Bmp4_3
secreted	16000	Igf1	insulin-like growth factor 1	NM_001111274 N	TCTGAGGAGACTGGAGATGTA	SI01073989	Mm_Igf1_3
secreted	16000	Igf1	insulin-like growth factor 1	NM_001111274 N	TCCACTCTCATTAAAGGGTAA	SI04953193	Mm_Igf1_7
secreted	16880	Lifr	leukemia inhibitory factor receptor	NM_001113386 N	TACCATGTTGCTGTAGACAAA	SI02715300	Mm_Lifr_7
secreted	16880	Lifr	leukemia inhibitory factor receptor	NM_001113386 N	AAGCCTTTACCTATTAATGAA	SI02692690	Mm_Lifr_6
secreted	235534	Acpl2	acid phosphatase-like 2	NM_153420	CAGCGTTGGGTCTGTATCTGA	SI04927944	Mm_Acpl2_6
secreted	235534	Acpl2	acid phosphatase-like 2	NM_153420	TTCGGTCCGAATTCTTTATAA	SI04927937	Mm_Acpl2_5
secreted	75019	Rnase10	ribonuclease, RNase A family, 10 (non-active)	NM_001162863 N	AAGAGTTTAATGCCTCCTTTA	SI01402772	Mm_Rnase10_4
secreted	75019	Rnase10	ribonuclease, RNase A family, 10 (non-active)	NM_001162863 N	CACCTTGGGTGTAGACTAGAA	SI01402765	Mm_Rnase10_3
secreted	18791	Plat	plasminogen activator, tissue	NM_008872	CTGGTGTGCATGATCAATAAA	SI02710022	Mm_Plat_5
secreted	18791	Plat	plasminogen activator, tissue	NM_008872	CAGGTACTTCACAACTTGTAA	SI00179907	Mm_Plat_4
secreted	69376	Zpbp2	zona pellucida binding protein 2	NM_001166494 N	AAGGGAATAGCCAGATAAATA	SI01483244	Mm_Zpbp2_4
secreted	69376	Zpbp2	zona pellucida binding protein 2	NM_001166494 N	TAGCAAAGTATAATATAATAA	SI01483237	Mm_Zpbp2_3
receptors	16195	Il6st	interleukin 6 signal transducer	NM_010560	AAGAAACTGCTTATTATTGAA	SI02712829	Mm_Il6st_7
receptors	16195	Il6st	interleukin 6 signal transducer	NM_010560	TCCCTTTGCTCTAAGAGAAA	SI00193816	Mm_Il6st_3
secreted	53324	Nptx2	neuronal pentraxin 2	NM_016789	CCGCTCCTTGCAAACCCTCAA	SI01330392	Mm_Nptx2_4
secreted	53324	Nptx2	neuronal pentraxin 2	NM_016789	CCGGGCGCAGGAGATCATCAA	SI01330385	Mm_Nptx2_3
secreted	193813	Mcfd2	multiple coagulation factor deficiency 2	NM_139295 NM_	CACCACTGGCCTGAATAATTA	SI01302336	Mm_Mcfd2_4
secreted	193813	Mcfd2	multiple coagulation factor deficiency 2	NM_139295 NM_	CTCTGGCTATCTGACAATAAA	SI01302329	Mm_Mcfd2_3
receptors	12986	Csf3r	colony stimulating factor 3 receptor (granulocyte)	NM_007782	CTGGACATTGGCCCTGATGTA	SI02732121	Mm_Csf3r_6
receptors	12986	Csf3r	colony stimulating factor 3 receptor (granulocyte)	NM_007782	CAGCTTCATCCTAAAGAGCTT	SI02708090	Mm_Csf3r_5
secreted	93721	Cpn1	carboxypeptidase N, polypeptide 1	NM_030703	CTGGGTAACCGGGAAGCCTTA	SI02745001	Mm_Cpn1_6
secreted	93721	Cpn1	carboxypeptidase N, polypeptide 1	NM_030703	CCGCAACTTCCCGGATCTCAA	SI02696484	Mm_Cpn1_5
secreted	68453	Gpihbp1	GPI-anchored HDL-binding protein 1	NM_026730	CCCGGAGCCAGAGAACTACAA	SI01307264	Mm_MGI:1915703_4
secreted	68453	Gpihbp1	GPI-anchored HDL-binding protein 1	NM_026730	CAGCGGAACCGACAAAGGTTA	SI01307257	Mm_MGI:1915703_3
receptors	235611	Plxnb1	plexin B1	NM_172775	ACCAAGCATATTCTTAGGGAA	SI02747591	Mm_Plxnb1_7
receptors	235611	Plxnb1	plexin B1	NM_172775	AAAGGAGAACTTAAAGCACAA	SI02722181	Mm_Plxnb1_6
secreted	209268	Igsf1	immunoglobulin superfamily, member 1	NM_177591 NM_	TTGACAGAGGAGATTGAAATA	SI04961516	Mm_Igsf1_5
secreted	209268	Igsf1	immunoglobulin superfamily, member 1	NM_177591 NM_	TAAGGAGTTTGTGTTACTGAA	SI01074752	Mm_Igsf1_4
secreted	56264	Cpxm1	carboxypeptidase X 1 (M14 family)	NM_019696	CTGCAATTTCCTACTCACCAA	SI02741781	Mm_Cpxm1_7
secreted	56264	Cpxm1	carboxypeptidase X 1 (M14 family)	NM_019696	CTGGACCTGTCCAGAACTGAA	SI02716966	Mm_Cpxm1_6
secreted	16323	Inhba	inhibin beta-A	NM_008380	ACCCATGTCCATGCTGTATTA	SI02687251	Mm_Inhba_5
secreted	16323	Inhba	inhibin beta-A	NM_008380	CAGGAGAGTGGTGCCAGTCTA	SI00175189	Mm_Inhba_4
secreted	14459	Gast	gastrin	NM_010257	AGGCCACGAATGGAGGAAGAA	SI04403924	Mm_Gast_5
secreted	14459	Gast	gastrin	NM_010257	CTGGTCTTAGTGCTGGCTCTA	SI02736853	Mm_Gast_4
secreted	12840	Col9a2	collagen, type IX, alpha 2	NM_007741	CCGGCCTGGCCAGGCAATCAA	SI05164474	Mm_Col9a2_6
secreted	12840	Col9a2	collagen, type IX, alpha 2	NM_007741	CAGCCAGGACAGAGCCTATTA	SI00168833	Mm_Col9a2_4
receptors	50498	Ebi3	Epstein-Barr virus induced gene 3	NM_015766	AACTTTGTGATTTAATTAGAA	SI02740584	Mm_Ebi3_4
receptors	50498	Ebi3	Epstein-Barr virus induced gene 3	NM_015766	CACGACTTTCACCCTCAGGAA	SI02693138	Mm_Ebi3_3
secreted	63859	Impg1	interphotoreceptor matrix proteoglycan 1	NM_022016	CAGAATTACCAGTATACAGAA	SI02742880	Mm_Impg1_8
secreted	63859	Impg1	interphotoreceptor matrix proteoglycan 1	NM_022016	CTCCAAGTTCAAGGAATCAAA	SI02717960	Mm_Impg1_7
secreted	215001	Wfikkn1	WAP, FS, Ig, KU, and NTR-containing protein 1	NM_001100454	GCCCTCCTTCCTGCTGAATAA	SI01472324	Mm_Wfikkn1_4
secreted	215001	Wfikkn1	WAP, FS, Ig, KU, and NTR-containing protein 1	NM_001100454	CTCGAGAAGAAGGCTTGTGAA	SI01472317	Mm_Wfikkn1_3
secreted	54635	Pdgfc	platelet-derived growth factor, C polypeptide	NM_019971	AGCTATGTTAATAGGAATTAA	SI01373820	Mm_Pdgfc_4
secreted	54635	Pdgfc	platelet-derived growth factor, C polypeptide	NM_019971	CACACACTTGATTGTGAACAA	SI01373813	Mm_Pdgfc_3
secreted	16774	Lama3	laminin, alpha 3	NM_010680	AAGCTTAATTACACCAAAGAA	SI01087604	Mm_Lama3_4
secreted	16774	Lama3	laminin, alpha 3	NM_010680	AAGATTGAAAGTATCAACCAA	SI01087597	Mm_Lama3_3
receptors	16880	Lifr	leukemia inhibitory factor receptor	NM_001113386 N	TACCATGTTGCTGTAGACAAA	SI02715300	Mm_Lifr_7
receptors	16880	Lifr	leukemia inhibitory factor receptor	NM_001113386 N	AAGCCTTTACCTATTAATGAA	SI02692690	Mm_Lifr_6
secreted	12310	Calca	calcitonin/calcitonin-related polypeptide, alpha	NM_001033954 N	ATGGCCACTCTCAGTGAAGAA	SI04946354	Mm_Calca_8
secreted	12310	Calca	calcitonin/calcitonin-related polypeptide, alpha	NM_001033954 N	CAGCATCTTGCTCCTGTACCA	SI04946347	Mm_Calca_7
secreted	100689	Spon2	spondin 2, extracellular matrix protein	NM_133903	CAGGTGGTCCTGGATCTTTAT	SI01431976	Mm_Spon2_4
secreted	100689	Spon2	spondin 2, extracellular matrix protein	NM_133903	CTCCATCAGCTTGAATGTGAA	SI01431969	Mm_Spon2_3
secreted	20562	Slit1	slit homolog 1 (Drosophila)	NM_015748	AACCTGTACATCAACAATGAA	SI01425781	Mm_Slit1_3
secreted	20562	Slit1	slit homolog 1 (Drosophila)	NM_015748	CAGGATAGACCTGAGCAACAA	SI01425788	Mm_Slit1_4
secreted	58859	Efemp2	epidermal growth factor-containing fibulin-like extracellular matrix protei	NM_001164352 N	CGGCCCATGCTCAACAACCAA	SI05184011	Mm_Efemp2_5
secreted	58859	Efemp2	epidermal growth factor-containing fibulin-like extracellular matrix protei	NM_001164352 N	CAGGGCTACGAGCCTGATGAA	SI00990332	Mm_Efemp2_4
receptors	22042	Tfrc	transferrin receptor	NM_011638	TTGAAGATCGTTAGTATCTAA	SI02739065	Mm_Tfrc_8
receptors	22042	Tfrc	transferrin receptor	NM_011638	ACCTTTCATCTTTGCATTTAA	SI02714432	Mm_Tfrc_7
secreted	20713	Serpini1	serine (or cysteine) peptidase inhibitor, clade I, member 1	NM_009250	AAGGAAGAATAAACATTATTA	SI02710694	Mm_Serpini1_7
secreted	20713	Serpini1	serine (or cysteine) peptidase inhibitor, clade I, member 1	NM_009250	CAGAATCAAACACCAAAGTAA	SI02734998	Mm_Serpini1_8
secreted	14588	Gfra4	glial cell line derived neurotrophic factor family receptor alpha 4	NM_001136063 N	TGCTCTGATTAGGAACATGAA	SI01011381	Mm_Gfra4_3
secreted	14588	Gfra4	glial cell line derived neurotrophic factor family receptor alpha 4	NM_001136063 N	TGCGAGCTTTACCGACGGGAA	SI01011388	Mm_Gfra4_4
secreted	12321	Calu	calumenin	NM_007594 NM_	GAGGAATATGACTACATGAAA	SI00940268	Mm_Calu_4
secreted	12321	Calu	calumenin	NM_007594 NM_	AAGGCATATTGAGATAGTGAA	SI00940261	Mm_Calu_3
secreted	22044	Trh	thyrotropin releasing hormone	NM_009426	GAGGAAGACGTTGAAGCCGAA	SI02735334	Mm_Trh_6
secreted	22044	Trh	thyrotropin releasing hormone	NM_009426	TCCAAAGAAGCTGGGAAGAAA	SI02711030	Mm_Trh_5
secreted	104079	Nxph3	neurexophilin 3	NM_130858	CAGCAAAGCTGTGGAGTTTCA	SI01333640	Mm_Nxph3_4
secreted	104079	Nxph3	neurexophilin 3	NM_130858	AGGGCTTTCCCTCCTATCCAA	SI01333633	Mm_Nxph3_3
receptors	16847	Lepr	leptin receptor	NM_001122899 N	CCTGATGATTATAGTCTGTTA	SI02737546	Mm_Lepr_5
receptors	16847	Lepr	leptin receptor	NM_001122899 N	TTGAAGCTAAATTTAATTCAA	SI01089200	Mm_Lepr_4
secreted	12628	Cfh	complement component factor h	NM_009888	AAGCATGGTTATGTGCCCAAA	SI02736167	Mm_Cfh_8
secreted	12628	Cfh	complement component factor h	NM_009888	TACCCAGATCTTCAGAAGAAA	SI02711800	Mm_Cfh_7
secreted	18781	Pla2g2c	phospholipase A2, group IIC	NM_008868	CAGGGACAAACTCCAGTGCTA	SI00179879	Mm_Pla2g2c_4
secreted	18781	Pla2g2c	phospholipase A2, group IIC	NM_008868	CAGCGCCTTCTTCTCCTATTA	SI00179872	Mm_Pla2g2c_3
secreted	22420	Wnt6	wingless-related MMTV integration site 6	NM_009526	CTCGCGCGTCATGGGCACCAA	SI04415362	Mm_Wnt6_6
secreted	22420	Wnt6	wingless-related MMTV integration site 6	NM_009526	AAAGCTCGTCATGAACTGTAA	SI04415355	Mm_Wnt6_5
receptors	209268	Igsf1	immunoglobulin superfamily, member 1	NM_177591 NM_	TTGACAGAGGAGATTGAAATA	SI04961516	Mm_Igsf1_5
receptors	209268	Igsf1	immunoglobulin superfamily, member 1	NM_177591 NM_	TAAGGAGTTTGTGTTACTGAA	SI01074752	Mm_Igsf1_4
secreted	66824	Pycard	PYD and CARD domain containing	NM_023258	TACCTTTGAGAACACATGTAA	SI00220885	Mm_Pycard_4
secreted	66824	Pycard	PYD and CARD domain containing	NM_023258	CAGCTGCAAACGACTAAAGAA	SI00220878	Mm_Pycard_3
secreted	17183	Matn4	matrilin 4	NM_013592	CTGCAGGTCCTAGGTGCTATA	SI01301692	Mm_Matn4_4
secreted	17183	Matn4	matrilin 4	NM_013592	CTGGACCAGGAGCCAGATTTA	SI01301685	Mm_Matn4_3
secreted	246779	Il27	interleukin 27	NM_145636	TGCCAGGAAGCTGCTCTCTGA	SI01075844	Mm_Il27_4
secreted	246779	Il27	interleukin 27	NM_145636	CTGGGATACCATCTTCCCAAT	SI01075837	Mm_Il27_3
secreted	54137	Acrbp	proacrosin binding protein	NM_001127340 N	CAGGCTGCAGTCAGACTCAGA	SI04955573	Mm_Acrbp_7
secreted	54137	Acrbp	proacrosin binding protein	NM_001127340 N	GAGGTGGAGTCTTCAGCAGAA	SI04955566	Mm_Acrbp_6
receptors	211323	Nrg1	neuregulin 1	NM_178591	AAAGTGGGTATTTGTGGACAA	SI04722865	Mm_Nrg1_8

receptors	211323	Nrg1	neuregulin 1	NM_178591	CAGGCCAGGCTGTCTAGTGTA	SI04722858	Mm_Nrg1_7
secreted	54615	Npff	neuropeptide FF-amide peptide precursor	NM_018787 NR_(	AGCTGTGATGCCTGCATGCAA	SI02741277	Mm_Npff_4
secreted	54615	Npff	neuropeptide FF-amide peptide precursor	NM_018787 NR_(	CAGGCCATGGACACACCTAGA	SI02693712	Mm_Npff_2
secreted	11516	Adcyap1	adenylate cyclase activating polypeptide 1	NM_009625	TTGATATATATTATAAGTATA	SI00890141	Mm_Adcyap1_3
secreted	11516	Adcyap1	adenylate cyclase activating polypeptide 1	NM_009625	CACAGAGAAATTATGCACTAA	SI00890148	Mm_Adcyap1_4
secreted	15530	Hspg2	perlecan (heparan sulfate proteoglycan 2)	NM_008305	ACGGAGGGTGCTAGCTGTGAA	SI04453491	Mm_Hspg2_5
secreted	15530	Hspg2	perlecan (heparan sulfate proteoglycan 2)	NM_008305	AACCCTCAACGCAACAGCCAA	SI02842994	Mm_Hspg2_4
secreted	22283	Ush2a	Usher syndrome 2A (autosomal recessive, mild) homolog (human)	NM_021408	TCCGGTGAGGACAAACGGCAA	SI04450733	Mm_Ush2a_7
secreted	22283	Ush2a	Usher syndrome 2A (autosomal recessive, mild) homolog (human)	NM_021408	CAGATACGTTTCATGCTGCAA	SI04450726	Mm_Ush2a_6
secreted	66848	Fuca2	fucosidase, alpha-L- 2, plasma	NM_025799	ATCATAAATCCTCATATATAA	SI01006712	Mm_Fuca2_4
secreted	66848	Fuca2	fucosidase, alpha-L- 2, plasma	NM_025799	CACCCTCATCTTGGACTTCTA	SI01006705	Mm_Fuca2_3
secreted	213350	Pddc1	Parkinson disease 7 domain containing 1	NM_172116	CTCCATCAGGACCTTGTGGAA	SI04414886	Mm_Pddc1_1
secreted	213350	Pddc1	Parkinson disease 7 domain containing 1	NM_172116	CTGTGCTGTGCTACCAATGAA	SI00920325	Mm_BC023835_3
receptors	22340	Vegfb	vascular endothelial growth factor B	NM_001185164 N	CACAGCCAATGTGAATGCAGA	SI01469132	Mm_Vegfb_4
receptors	22340	Vegfb	vascular endothelial growth factor B	NM_001185164 N	CTGCCATCTAACAATTGTCAA	SI01469125	Mm_Vegfb_3
secreted	80857	Fgf20	fibroblast growth factor 20	NM_030610	AAAGGAGAACTTTATGGATCA	SI01002400	Mm_Fgf20_4
secreted	80857	Fgf20	fibroblast growth factor 20	NM_030610	AAGGATGGAACTCCAAGAGAT	SI01002393	Mm_Fgf20_3
secreted	14221	Fjx1	four jointed box 1 (Drosophila)	NM_010218	AAGGCTATGAATTCAGGACTA	SI01003261	Mm_Fjx1_3
secreted	14221	Fjx1	four jointed box 1 (Drosophila)	NM_010218	CCGGCATGTGGGACAAGTATA	SI01003268	Mm_Fjx1_4
secreted	13363	Dhh	desert hedgehog	NM_007857	CAGGGCATGTAGGCAGAGTTA	SI02708223	Mm_Dhh_6
secreted	13363	Dhh	desert hedgehog	NM_007857	CAGGTGAGTTAATAAAGGGAA	SI02666699	Mm_Dhh_5
secreted	16156	Il11	interleukin 11	NM_008350	TGGCTTATTTATACTTATTTA	SI02667476	Mm_Il11_5
secreted	16156	Il11	interleukin 11	NM_008350	CACCATCGATACCGCCCTTTA	SI00174573	Mm_Il11_4
secreted	56401	Lepre1	leprecan 1	NM_001042411 N	CCGAGGAATACCGACGTCGAA	SI04946298	Mm_Lepre1_6
secreted	56401	Lepre1	leprecan 1	NM_001042411 N	CAGAACCTCGACTATTACCAA	SI04946291	Mm_Lepre1_5
secreted	14172	Fgf18	fibroblast growth factor 18	NM_008005	CGCGAGAACCAGCAAGATGTA	SI01002344	Mm_Fgf18_4
secreted	14172	Fgf18	fibroblast growth factor 18	NM_008005	CTCTAGGAGGTGACAATTCAA	SI01002337	Mm_Fgf18_3
receptors	21937	Tnfrsf1a	tumor necrosis factor receptor superfamily, member 1a	NM_011609	AAGGAAAGTATGTCCATTCTA	SI01452416	Mm_Tnfrsf1a_4
receptors	21937	Tnfrsf1a	tumor necrosis factor receptor superfamily, member 1a	NM_011609	TCGACTCTTGTAAATACACTA	SI01452409	Mm_Tnfrsf1a_3
secreted	16949	Loxl1	lysyl oxidase-like 1	NM_010729	GGGAGTGAACATGGACCCAAA	SI01294636	Mm_Loxl1_4
secreted	16949	Loxl1	lysyl oxidase-like 1	NM_010729	CACGGGCAAGAAGGTAGCCGA	SI01294629	Mm_Loxl1_3
secreted	66350	Pla2g12a	phospholipase A2, group XIIA	NM_023196 NM_	TGCTGGTGTCGTTATGAAGAA	SI01380288	Mm_Pla2g12a_4
secreted	66350	Pla2g12a	phospholipase A2, group XIIA	NM_023196 NM_	CTGTTCCACGCTATGGATATA	SI01380281	Mm_Pla2g12a_3
secreted	16772	Lama1	laminin, alpha 1	NM_008480	AAAGGTTATGCTGGAGATAAA	SI01087576	Mm_Lama1_4
secreted	16772	Lama1	laminin, alpha 1	NM_008480	CAGAGGTATTTAATAAAGGAA	SI01087569	Mm_Lama1_3
receptors	18793	Plaur	plasminogen activator, urokinase receptor	NM_011113	AGGCTTAGATGTGCTGGGAAA	SI01380792	Mm_Plaur_4
receptors	18793	Plaur	plasminogen activator, urokinase receptor	NM_011113	TTCGGGAATGGCAAGATGATA	SI01380785	Mm_Plaur_3
secreted	78369	Icam4	intercellular adhesion molecule 4, Landsteiner-Wiener blood group	NM_023892	CGCCAGGATCACTGCTTACAA	SI01072456	Mm_Icam4_4
secreted	78369	Icam4	intercellular adhesion molecule 4, Landsteiner-Wiener blood group	NM_023892	CCCAGCAAGAGTGGATGCAAA	SI01072449	Mm_Icam4_3
secreted	77613	Prss36	protease, serine, 36	NM_001081374	CTGCATCAGCTTTCAGATAAT	SI04418071	Mm_Prss36_1
secreted	77613	Prss36	protease, serine, 36	NM_001081374	CACCATCCTGATACCGGACAA	SI02726199	Mm_LOC546003_3
secreted	11674	Aldoa	aldolase A, fructose-bisphosphate	NM_001177307 N	CCCACTGCCAATAAACAACTA	SI05428808	Mm_Aldoa_5
secreted	11674	Aldoa	aldolase A, fructose-bisphosphate	NM_001177307 N	AGGCACATTGCTGAAGCCCAA	SI00896245	Mm_Aldoa_3
secreted	13614	Edn1	endothelin 1	NM_010104	CAGCTGGTGGAAGGAAGGAAA	SI00989940	Mm_Edn1_4
secreted	13614	Edn1	endothelin 1	NM_010104	TAGATATTTATATTCAAACAA	SI00989933	Mm_Edn1_3
secreted	230868	Igsf21	immunoglobin superfamily, member 21	NM_198610	CGCAGTGACCCTGAAGTGTAA	SI00926317	Mm_BC055811_3
secreted	230868	Igsf21	immunoglobin superfamily, member 21	NM_198610	CAGCTTCTGGACACCATGCAA	SI00926324	Mm_BC055811_4
receptors	58235	Pvrl1	poliovirus receptor-related 1	NM_021424	CCCGAAGCAAAGCAACATTTA	SI02742635	Mm_Pvrl1_8
receptors	58235	Pvrl1	poliovirus receptor-related 1	NM_021424	CAGGGAGAGCCTTACGCTCAA	SI02717722	Mm_Pvrl1_7
secreted	333315	Frem3	Fras1 related extracellular matrix protein 3	NM_001167898	CAGGGTAACGATCCTGGCTGA	SI04414550	Mm_Frem3_6
secreted	333315	Frem3	Fras1 related extracellular matrix protein 3	NM_001167898	CCCTGGCATTCTGATCGCCAA	SI04454212	Mm_Frem3_7
secreted	21950	Tnfsf9	tumor necrosis factor (ligand) superfamily, member 9	NM_009404	ACCGCGGATGCCAGACATCCA	SI02735285	Mm_Tnfsf9_7
secreted	21950	Tnfsf9	tumor necrosis factor (ligand) superfamily, member 9	NM_009404	CACAATCACCACCTCGCCCAA	SI00184632	Mm_Tnfsf9_4
secreted	77701	Lcn12	lipocalin 12	NM_029958	CCGGGCCTTGCTGAATTTCTT	SI01088332	Mm_Lcn12_4
secreted	77701	Lcn12	lipocalin 12	NM_029958	CAGAGAGAATATACAGGTCAT	SI01088325	Mm_Lcn12_3
receptors	21812	Tgfbr1	transforming growth factor, beta receptor I	NM_009370	TAGAGAAATATTTCTATGTAA	SI01447040	Mm_Tgfbr1_4
receptors	21812	Tgfbr1	transforming growth factor, beta receptor I	NM_009370	CACAGACAAAGTTATACACAA	SI02735194	Mm_Tgfbr1_6
secreted	114661	Prss28	protease, serine, 28	NM_053259	CAGGATCATCATCCACCCTGA	SI02696876	Mm_MGI:2149951_5
secreted	114661	Prss28	protease, serine, 28	NM_053259	CGCAACAATCTGCCATCAAT	SI00231301	Mm_MGI:2149951_3
secreted	11491	Adam17	a disintegrin and metallopeptidase domain 17	NM_009615	TGGGAAGTTTCTGGCAGATAA	SI02711289	Mm_Adam17_7
secreted	11491	Adam17	a disintegrin and metallopeptidase domain 17	NM_009615	CAGAGCCGAGTTGACAGCAAA	SI02735642	Mm_Adam17_8
secreted	18074	Nid2	nidogen 2	NM_008695	GAGAGGGTTCGTATCACTCAA	SI04449284	Mm_Nid2_7
secreted	18074	Nid2	nidogen 2	NM_008695	ACCGATGTCATGAGGCAGCTA	SI04401453	Mm_Nid2_6
secreted	78405	Ntf5	neurotrophin 5	NM_198190	CTGTATGAGAATCTATCTTAA	SI02748816	Mm_Ntf5_7
secreted	78405	Ntf5	neurotrophin 5	NM_198190	AAGAATCAAATCTCTCTCAAA	SI02723315	Mm_Ntf5_6
secreted	68792	Srpx2	sushi-repeat-containing protein, X-linked 2	NM_001083895 N	CACTGTGATGGTGGTTATGAA	SI02744196	Mm_Srpx2_7
secreted	68792	Srpx2	sushi-repeat-containing protein, X-linked 2	NM_001083895 N	CAACATGGTGTTAATTGACAA	SI02719080	Mm_Srpx2_6
secreted	20655	Sod1	superoxide dismutase 1, soluble	NM_011434	AACCATCCACTTCGAGCAGAA	SI04716005	Mm_Sod1_7
secreted	20655	Sod1	superoxide dismutase 1, soluble	NM_011434	TCAGGACAAATTACAGGATTA	SI04715998	Mm_Sod1_6
secreted	110611	Hdlbp	high density lipoprotein (HDL) binding protein	NM_133808	CCCAGTGTTAACCGTACAGAA	SI01064168	Mm_Hdlbp_4
secreted	110611	Hdlbp	high density lipoprotein (HDL) binding protein	NM_133808	AAGGATCTAATCATCGAGCAA	SI01064161	Mm_Hdlbp_3
secreted	16776	Lama5	laminin, alpha 5	NM_001081171	AACCAAGAGGTGGAAACTCAA	SI01087632	Mm_Lama5_4
secreted	16776	Lama5	laminin, alpha 5	NM_001081171	CCCTGTGGAGGTACTCATTAA	SI01087625	Mm_Lama5_3
secreted	56348	Hsd17b12	hydroxysteroid (17-beta) dehydrogenase 12	NM_019657	CACATTATATTTGGTCTGGAA	SI02741697	Mm_Hsd17b12_6
secreted	56348	Hsd17b12	hydroxysteroid (17-beta) dehydrogenase 12	NM_019657	CACCATCAAGAAACTGATAAA	SI02716924	Mm_Hsd17b12_5
secreted	22070	Tpt1	tumor protein, translationally-controlled 1	NM_009429	CACATCCTTGCTAATTTCAAT	SI05392653	Mm_Tpt1_8
secreted	22070	Tpt1	tumor protein, translationally-controlled 1	NM_009429	CGAGCTGTTCTCCGACATCTA	SI02652636	Mm_Tpt1_5
secreted	56693	Crtap	cartilage associated protein	NM_019922	CAGAGTGACAGGGTCATGCAA	SI04941300	Mm_Crtap_6
secreted	56693	Crtap	cartilage associated protein	NM_019922	AACGTCCATTTCCGACATGGA	SI04941293	Mm_Crtap_5
secreted	320202	Lefty2	left-right determination factor 2	NM_177099	CTGGTGAATTCTATGAAACTA	SI02722804	Mm_Lefty2_6
secreted	320202	Lefty2	left-right determination factor 2	NM_177099	AAGCAAGTATAGAATACATAA	SI02678186	Mm_Lefty2_5
secreted	72043	Sulf2	sulfatase 2	NM_028072	CAGCGCCAACAACAACGTA	SI04451293	Mm_Sulf2_5
secreted	72043	Sulf2	sulfatase 2	NM_028072	TGCAACCGGCTTCATAGAATA	SI01437492	Mm_Sulf2_4
secreted	21826	Thbs2	thrombospondin 2	NM_011581	ATGCACCTGCAAGAAATTTAA	SI01447488	Mm_Thbs2_4
secreted	21826	Thbs2	thrombospondin 2	NM_011581	CTGGAGGATGCTAGGACAGAA	SI01447481	Mm_Thbs2_3
secreted	56373	Cpb2	carboxypeptidase B2 (plasma)	NM_019775	AAGAAATATCGACCACATTAA	SI02741914	Mm_Cpb2_7
secreted	56373	Cpb2	carboxypeptidase B2 (plasma)	NM_019775	CAGAATCTTACTACAACGTAT	SI02717064	Mm_Cpb2_6
secreted	22419	Wnt5b	wingless-related MMTV integration site 5B	NM_009525	AAGCTCTTGTGCCAAGAGAAA	SI01472828	Mm_Wnt5b_4
secreted	22419	Wnt5b	wingless-related MMTV integration site 5B	NM_009525	ATGGCTGATGTCGCCTGCAAA	SI01472821	Mm_Wnt5b_3
secreted	12827	Col4a2	collagen, type IV, alpha 2	NM_009932	CAGCTTGGTGCTTACTCTTAA	SI00956004	Mm_Col4a2_4
secreted	12827	Col4a2	collagen, type IV, alpha 2	NM_009932	AACGCCATTTATCGAGTGTAA	SI00955997	Mm_Col4a2_3
secreted	11875	Art5	ADP-ribosyltransferase 5	NM_007491	CGGAGTAGCGATTATGGTGTA	SI04715263	Mm_Art5_6
secreted	11875	Art5	ADP-ribosyltransferase 5	NM_007491	CTGTCCGATTAGGACAGTTTA	SI04446533	Mm_Art5_5
receptors	13649	Egfr	epidermal growth factor receptor	NM_007912 NM_	TTCGAGAACCTAGAAATAATA	SI02732394	Mm_Egfr_5
receptors	13649	Egfr	epidermal growth factor receptor	NM_007912 NM_	CAGATGGATGTCAACCCTGAA	SI00990836	Mm_Egfr_4
secreted	19215	Ptgds	prostaglandin D2 synthase (brain)	NM_008963	CAACCGGATAAGTGCATTCAA	SI01392272	Mm_Ptgds_4
secreted	19215	Ptgds	prostaglandin D2 synthase (brain)	NM_008963	AAGCTGGTTCCGGGAGAAGAA	SI01392265	Mm_Ptgds_3
secreted	238328	Vash1	vasohibin 1	NM_177354	CAGAGAGATGAGGAACCCGAA	SI01007748	Mm_G630009D10Rik_4
secreted	238328	Vash1	vasohibin 1	NM_177354	GACACAATTCTTTGAAATTAA	SI01007741	Mm_G630009D10Rik_3
secreted	18590	Pdgfa	platelet derived growth factor, alpha	NM_008808	CCAGGTGAGGTTAGAGGAACA	SI00179606	Mm_Pdgfa_4
secreted	18590	Pdgfa	platelet derived growth factor, alpha	NM_008808	CCGCAGTGTCAAGGTGGCCAA	SI00179599	Mm_Pdgfa_3
secreted	15235	Mst1	macrophage stimulating 1 (hepatocyte growth factor-like)	NM_008243	CCGCTTTGTGCCAGAGAAATA	SI02686999	Mm_Mst1_6
secreted	15235	Mst1	macrophage stimulating 1 (hepatocyte growth factor-like)	NM_008243	CTCGATGAATGTCATCTCCAA	SI02667315	Mm_Mst1_5

receptors	14972	H2-K1	histocompatibility 2, K1, K region	NM_001001892	TAGATGAATAAACTTCCAGAA	SI01061704	Mm_H2-K1_4
receptors	14972	H2-K1	histocompatibility 2, K1, K region	NM_001001892	CACAGCAGACCTGAAGATAAA	SI01061697	Mm_H2-K1_3
secreted	13590	Lefty1	left right determination factor 1	NM_010094	CTCAGGGACACACACATCCAA	SI02736573	Mm_Lefty1_4
secreted	13590	Lefty1	left right determination factor 1	NM_010094	TTCCCTTAATTTGTCGCTGAA	SI02689932	Mm_Lefty1_3
secreted	23794	Adamts5	a disintegrin-like and metallopeptidase (reprolysin type) with thrombospc	NM_011782	CACAGGGAAGAGGGCCATATA	SI02739233	Mm_Adamts5_7
secreted	23794	Adamts5	a disintegrin-like and metallopeptidase (reprolysin type) with thrombospc	NM_011782	CAGCACCAACATAACCAGCTA	SI02714572	Mm_Adamts5_6
secreted	239759	Liph	lipase, member H	NM_001083894 N	CTCCGTGAGACTGATGCTCTA	SI01090516	Mm_Liph_4
secreted	239759	Liph	lipase, member H	NM_001083894 N	TAGGGTTTGGCTCTTACGTAA	SI01090509	Mm_Liph_3
secreted	16819	Lcn2	lipocalin 2	NM_008491	ACCAATGCATTGACAACTGAA	SI01088381	Mm_Lcn2_3
secreted	16819	Lcn2	lipocalin 2	NM_008491	AGGACTCAACTCAGAACTTGA	SI01088388	Mm_Lcn2_4
secreted	67866	Wfdc1	WAP four-disulfide core domain 1	NM_023395	CATGGACAGTGTGTTAAGCAA	SI01472184	Mm_Wfdc1_4
secreted	67866	Wfdc1	WAP four-disulfide core domain 1	NM_023395	CAGCCAAAGAATGACCCTAGA	SI01472177	Mm_Wfdc1_3
receptors	14254	Flt1	FMS-like tyrosine kinase 1	NM_010228	TCGGCTGTCCATGAAAGTGAA	SI01003660	Mm_Flt1_4
receptors	14254	Flt1	FMS-like tyrosine kinase 1	NM_010228	CCAGCTATAGTTACTAAACTA	SI01003653	Mm_Flt1_3
secreted	11604	Agrp	agouti related protein	NM_007427	AAGTCTGAATGGCCTCAAGAA	SI00891688	Mm_Agrp_4
secreted	11604	Agrp	agouti related protein	NM_007427	CAAGCTGGGTACGGCCACGAA	SI00891681	Mm_Agrp_3
secreted	16326	Inhbe	inhibin beta E	NM_008382	CTGGAATCGAGAATCAGTAAA	SI04715669	Mm_Inhbe_5
secreted	16326	Inhbe	inhibin beta E	NM_008382	AAGGGTGGGCAGATTAGGAAA	SI01076432	Mm_Inhbe_4
secreted	22141	Tub	tubby candidate gene	NM_021885	CAGGTTGGACACTCGACGAAA	SI04405135	Mm_Tub_8
secreted	22141	Tub	tubby candidate gene	NM_021885	CAGCGGTGTGCTATGAGACAA	SI04405128	Mm_Tub_7
secreted	18643	Pfn1	profilin 1	NM_011072	CCGGGACTCACTGCTGCAAGA	SI05164488	Mm_Pfn1_5
secreted	18643	Pfn1	profilin 1	NM_011072	CACTGTCACCATGACTGCCAA	SI01375864	Mm_Pfn1_4
receptors	81897	Tlr9	toll-like receptor 9	NM_031178	CTGGCACACAATGACATTCAT	SI01449616	Mm_Tlr9_4
receptors	81897	Tlr9	toll-like receptor 9	NM_031178	AGCGAGAACTTTCTCTATGAA	SI01449609	Mm_Tlr9_3
secreted	20316	Sdf2	stromal cell derived factor 2	NM_009143	AGCTTTCTTCATGCTGGTAAA	SI01412460	Mm_Sdf2_4
secreted	20316	Sdf2	stromal cell derived factor 2	NM_009143	TTCCATCTTGGCGTAGTTCTA	SI01412453	Mm_Sdf2_3
receptors	21808	Tgfb2	transforming growth factor, beta 2	NM_009367	CCCGGAGGTGATTTCCATCTA	SI02735180	Mm_Tgfb2_7
receptors	21808	Tgfb2	transforming growth factor, beta 2	NM_009367	AAGGAGTTTGAACTCAAATAA	SI02710855	Mm_Tgfb2_6
secreted	21667	Tdgf1	teratocarcinoma-derived growth factor 1	NM_011562	CACAGTGAATCCCTAATGTTA	SI01445136	Mm_Tdgf1_4
secreted	21667	Tdgf1	teratocarcinoma-derived growth factor 1	NM_011562	CACGGAGATCTTGGCTGCTAA	SI01445129	Mm_Tdgf1_3
secreted	19701	Ren1	renin 1 structural	NM_031192	CGGGTCCGACTTCACCATCCA	SI02745127	Mm_Ren1_5
secreted	19701	Ren1	renin 1 structural	NM_031192	CAAGGAGAAGAGAATAGAAGA	SI02696575	Mm_Ren1_4
secreted	71951	Gpc2	glypican 2 (cerebroglycan)	NM_172412	CTGCCCGGCATAGAAAGTTTA	SI01055404	Mm_Gpc2_4
secreted	71951	Gpc2	glypican 2 (cerebroglycan)	NM_172412	ACCCTGCTTGTACTTCGATAA	SI01055397	Mm_Gpc2_3
secreted	16002	Igf2	insulin-like growth factor 2	NM_001122736 N	AACCATCAGTGAATCAAATTA	SI02737245	Mm_Igf2_8
secreted	16002	Igf2	insulin-like growth factor 2	NM_001122736 N	AAGGTGCTCGGAGGCCACAAA	SI04774679	Mm_Igf2_9
secreted	21926	Tnf	tumor necrosis factor	NM_013693	ACCCTGGTATGAGCCCATATA	SI00207690	Mm_Tnf_3
secreted	21926	Tnf	tumor necrosis factor	NM_013693	CACGCTCTTCTGTCTACTGAA	SI00207697	Mm_Tnf_4
secreted	242939	Cpz	carboxypeptidase Z	NM_153107	TGGGCGCAGCTTTGAAGGCAA	SI02747171	Mm_Cpz_7
secreted	242939	Cpz	carboxypeptidase Z	NM_153107	CTCGGAGTACCTGCTGGGCAA	SI02721803	Mm_Cpz_6
secreted	20856	Stc2	stanniocalcin 2	NM_011491	ATCTATGTGTCTGTCATCTAA	SI02738890	Mm_Stc2_7
secreted	20856	Stc2	stanniocalcin 2	NM_011491	TTGCATGACGTTTCTGCACAA	SI02714250	Mm_Stc2_6
secreted	67963	Npc2	Niemann Pick type C2	NM_023409	TGGCTGTTTGCTGTTGGATTA	SI02718205	Mm_Npc2_5
secreted	67963	Npc2	Niemann Pick type C2	NM_023409	TTGCTTAGGGATGTCAGATAA	SI00221032	Mm_Npc2_3
secreted	54723	Tfip11	tuftelin interacting protein 11	NM_018783	CTCCGTGCCCATGAACTTTAA	SI01446872	Mm_Tfip11_4
secreted	54723	Tfip11	tuftelin interacting protein 11	NM_018783	CACCGTCCTTCTGAGAAATAA	SI01446865	Mm_Tfip11_3
secreted	23948	Mmp17	matrix metallopeptidase 17	NM_011846	CACAGATGTCCTATACAGCAA	SI02714698	Mm_Mmp17_5
secreted	23948	Mmp17	matrix metallopeptidase 17	NM_011846	CAGGACTTATTTCTTTAAGGA	SI02739366	Mm_Mmp17_6
secreted	11538	Adnp	activity-dependent neuroprotective protein	NM_009628	TACGAGAAAGACACCAAGTTA	SI02849931	Mm_Adnp_4
secreted	11538	Adnp	activity-dependent neuroprotective protein	NM_009628	CAGGCTGACAATGTAGAGCAA	SI02849924	Mm_Adnp_3
secreted	66549	Aggf1	angiogenic factor with G patch and FHA domains 1	NM_025630	TGCAGTAATAATAGTGAAGAA	SI00891380	Mm_Aggf1_4
secreted	66549	Aggf1	angiogenic factor with G patch and FHA domains 1	NM_025630	TTGGGCGATTTCAGATTATTA	SI00891373	Mm_Aggf1_3
secreted	19143	St14	suppression of tumorigenicity 14 (colon carcinoma)	NM_011176	CAAGGACTATGTGGAGATCAA	SI01434636	Mm_St14_4
secreted	19143	St14	suppression of tumorigenicity 14 (colon carcinoma)	NM_011176	CAGGCCCTGATTGCAACAGAA	SI01434629	Mm_St14_3
secreted	13857	Epor	erythropoietin receptor	NM_010149	CTGTCTGTATATAGAAATATA	SI02669982	Mm_Epor_5
secreted	13857	Epor	erythropoietin receptor	NM_010149	CACCTACTTGGTATTGGATAA	SI00190533	Mm_Epor_4
secreted	14734	Gpc3	glypican 3	NM_016697	CGGGATGGTGAAAGTGAAGAA	SI01055432	Mm_Gpc3_4
secreted	14734	Gpc3	glypican 3	NM_016697	CGGGATGAAGAATCAGTTTAA	SI01055425	Mm_Gpc3_3
secreted	22408	Wnt1	wingless-related MMTV integration site 1	NM_021279	CCGGATCAGTATTTCCTTCCA	SI01472541	Mm_Wnt1_3
secreted	22408	Wnt1	wingless-related MMTV integration site 1	NM_021279	TAGGTCCCTTGAGTTCTCTAA	SI01472548	Mm_Wnt1_4
secreted	140703	Emid1	EMI domain containing 1	NM_080595	CACGTCAGCTGCCTAAAGTTA	SI00993384	Mm_Emid1_4
secreted	140703	Emid1	EMI domain containing 1	NM_080595	AGGCTGCTTAATCTAACTGAA	SI00993377	Mm_Emid1_3
receptors	15200	Hbegf	heparin-binding EGF-like growth factor	NM_010415	TTGGTCATTGATACCTATCAA	SI02737147	Mm_Dtr_6
receptors	15200	Hbegf	heparin-binding EGF-like growth factor	NM_010415	TGGGTCCTATTTGCTCTGTAA	SI02712633	Mm_Dtr_5
secreted	83554	Fstl3	follistatin-like 3	NM_031380	CACCGCATAAACAGACATGTT	SI01006264	Mm_Fstl3_4
secreted	83554	Fstl3	follistatin-like 3	NM_031380	CCCAGACTTGCGCGTCATGTA	SI01006257	Mm_Fstl3_3
secreted	243914	Lgi4	leucine-rich repeat LGI family, member 4	NM_144556	TGGTGTGATGACCCTATTATA	SI01089732	Mm_Lgi4_4
secreted	243914	Lgi4	leucine-rich repeat LGI family, member 4	NM_144556	ATGAGATAATGTCCGAATAAA	SI01089725	Mm_Lgi4_3
secreted	14828	Hspa5	heat shock protein 5	NM_001163434 N	CCGCGTGGAGATCATAGCCAA	SI02717981	Mm_Hspa5_7
secreted	14828	Hspa5	heat shock protein 5	NM_001163434 N	AAGAACCAGATTGGAGATAAA	SI02694916	Mm_Hspa5_6
secreted	74511	Lrrc17	leucine rich repeat containing 17	NM_028977	AACTTTGACTATGGCGTGTTA	SI02850701	Mm_Lrrc17_4
secreted	74511	Lrrc17	leucine rich repeat containing 17	NM_028977	TTGAAGATGTTCACGAACTAA	SI02850694	Mm_Lrrc17_3
secreted	67573	Loxl4	lysyl oxidase-like 4	NM_001164311 N	CAGGCCAGTCTCTTAGAATCA	SI05183941	Mm_Loxl4_5
secreted	67573	Loxl4	lysyl oxidase-like 4	NM_001164311 N	CCGTGTGGAAGTGCTCATGAA	SI01294692	Mm_Loxl4_4
secreted	23886	Gdf15	growth differentiation factor 15	NM_011819	CTGGGTGGATGGATTGTGTAT	SI04777815	Mm_Gdf15_7
secreted	23886	Gdf15	growth differentiation factor 15	NM_011819	CCGAGAGGACTCGAACTCAGA	SI04774826	Mm_Gdf15_6
secreted	18119	Nodal	nodal	NM_013611	CCAGAGCCTGCTGAAACGATA	SI01328936	Mm_Nodal_4
secreted	18119	Nodal	nodal	NM_013611	CAGGTGGACTTCAACCTGATT	SI01328929	Mm_Nodal_3
receptors	22359	Vldlr	very low density lipoprotein receptor	NM_001161420 N	CAGCCTGTGATACATACCAAA	SI02715482	Mm_Vldlr_7
receptors	22359	Vldlr	very low density lipoprotein receptor	NM_001161420 N	CAGGAATGAATGGATTTGATA	SI02692844	Mm_Vldlr_6
secreted	50498	Ebi3	Epstein-Barr virus induced gene 3	NM_015766	AACTTTGTGATTTAATTAGAA	SI02740584	Mm_Ebi3_4
secreted	50498	Ebi3	Epstein-Barr virus induced gene 3	NM_015766	CACGACTTTCACCCTCAGGAA	SI02693138	Mm_Ebi3_3
secreted	14595	B4galt1	UDP-Gal:betaGlcNAc beta 1,4- galactosyltransferase, polypeptide 1	NM_022305	CTGCTCAATATTGGCTTTCAA	SI02717974	Mm_B4galt1_6
secreted	14595	B4galt1	UDP-Gal:betaGlcNAc beta 1,4- galactosyltransferase, polypeptide 1	NM_022305	TTCGTAGTATTTGAAAGAATA	SI02674420	Mm_B4galt1_5
receptors	14725	Lrp2	low density lipoprotein receptor-related protein 2	NM_001081088	CTGGTATCTGGCCAACGATAA	SI04415747	Mm_Lrp2_8
receptors	14725	Lrp2	low density lipoprotein receptor-related protein 2	NM_001081088	CAAGTGGACGATACTGTGGTA	SI04415740	Mm_Lrp2_7
secreted	12824	Col2a1	collagen, type II, alpha 1	NM_001113515 N	CTGAAGGATGGCTGCACGAAA	SI04952927	Mm_Col2a1_8
secreted	12824	Col2a1	collagen, type II, alpha 1	NM_001113515 N	GACGCTGGTCCTCAAGGCAAA	SI04746161	Mm_Col2a1_7
secreted	75426	Igfbpl1	insulin-like growth factor binding protein-like 1	NM_018741	CACTGTCTGTTTGTAATTCTA	SI01074185	Mm_Igfbpl1_3
secreted	75426	Igfbpl1	insulin-like growth factor binding protein-like 1	NM_018741	ATGAGTGTCATGGGTAAACAA	SI01074192	Mm_Igfbpl1_4
secreted	12931	Crlf1	cytokine receptor-like factor 1	NM_018827	ACGCATACTGCTCGAACCTTA	SI02716553	Mm_Crlf1_5
secreted	12931	Crlf1	cytokine receptor-like factor 1	NM_018827	TACCATCTGGGCAACAAAGAA	SI02693768	Mm_Crlf1_4
secreted	30806	Adamts8	a disintegrin-like and metallopeptidase (reprolysin type) with thrombospc	NM_013906	CTGGACCTTGCTTATGACCAA	SI00889840	Mm_Adamts8_4
secreted	30806	Adamts8	a disintegrin-like and metallopeptidase (reprolysin type) with thrombospc	NM_013906	ACCGTGAATGTGATAATCCAA	SI00889833	Mm_Adamts8_3
receptors	16177	Il1r1	interleukin 1 receptor, type I	NM_001123382 N	CACCTACATAGAAGTAAACAA	SI02733199	Mm_Il1r1_7
receptors	16177	Il1r1	interleukin 1 receptor, type I	NM_001123382 N	CAGATTCTATTCAGTTCATTA	SI02709098	Mm_Il1r1_6
secreted	100952	Emilin1	elastin microfibril interfacer 1	NM_133918	CCGTACTAAACTATTCAGGAA	SI00993440	Mm_Emilin1_4
secreted	100952	Emilin1	elastin microfibril interfacer 1	NM_133918	CAGGTGAAGAGTCTGACCAAA	SI00993433	Mm_Emilin1_3
secreted	94214	Spock2	sparc/osteonectin, cwcv and kazal-like domains proteoglycan 2	NM_052994	CACAAGGTGTGTGTTGCTCAA	SI01431892	Mm_Spock2_4
secreted	94214	Spock2	sparc/osteonectin, cwcv and kazal-like domains proteoglycan 2	NM_052994	ATGGACATCCCTGATAGATTA	SI01431885	Mm_Spock2_3
receptors	20737	Spn	sialophorin	NM_001037810 N	GCCCTGTGCCTTAACCATTAA	SI04939186	Mm_Spn_6
receptors	20737	Spn	sialophorin	NM_001037810 N	CCAGGGCTCTTTAGTACTAGA	SI04939179	Mm_Spn_5
secreted	16950	Loxl3	lysyl oxidase-like 3	NM_013586	CAGGGAGAAGGCAGAGTGGAA	SI01294664	Mm_Loxl3_4

secreted	16950	Loxl3	lysyl oxidase-like 3	NM_013586	AAGGAGAGAATAGACAGAGAA	SI01294657	Mm_Loxl3_3
secreted	30060	Mfi2	antigen p97 (melanoma associated) identified by monoclonal antibodies 1	NM_013900	CAGGAGGAATTCCAATGTTAC	SI01304380	Mm_Mfi2_4
secreted	30060	Mfi2	antigen p97 (melanoma associated) identified by monoclonal antibodies 1	NM_013900	TTCAAAGATGCTACAGTCCGA	SI01304373	Mm_Mfi2_3
secreted	12870	Cp	ceruloplasmin	NM_001042611 N	AAGGTTCTCTATATAAGGAAA	SI02686222	Mm_Cp_6
secreted	12870	Cp	ceruloplasmin	NM_001042611 N	TCCCATGGGCTTATTACTCAA	SI02707978	Mm_Cp_7
receptors	14182	Fgfr1	fibroblast growth factor receptor 1	NM_001079908 N	ATCGGGCTGGATAAGGACAAA	SI01002652	Mm_Fgfr1_4
receptors	14182	Fgfr1	fibroblast growth factor receptor 1	NM_001079908 N	AGCCTTGTTACCAACCTCTAA	SI01002645	Mm_Fgfr1_3
secreted	266614	Ly6g5b	lymphocyte antigen 6 complex, locus G5B	NM_148939	ATGGTGATCACCATCTATAAA	SI01297408	Mm_Ly6g5b_4
secreted	266614	Ly6g5b	lymphocyte antigen 6 complex, locus G5B	NM_148939	ACCAATCAGACTACTTGTATA	SI01297401	Mm_Ly6g5b_3
secreted	224116	Muc20	mucin 20	NM_001145874 N	CTGCACCATCATCCCTAGATA	SI05143488	Mm_Muc20_6
secreted	224116	Muc20	mucin 20	NM_001145874 N	CTGATTCAGTATGTACCTCTA	SI05143481	Mm_Muc20_5
secreted	20737	Spn	sialophorin	NM_001037810 N	CCAGGGCTCTTTAGTACTAGA	SI04939179	Mm_Spn_5
secreted	20737	Spn	sialophorin	NM_001037810 N	GCCCTGTGCCTTAACCATTAA	SI04939186	Mm_Spn_6
secreted	101401	Adamts9	a disintegrin-like and metallopeptidase (reprolysin type) with thrombospo	NM_175314	CAGCTAACCGTTTCTGATCAA	SI04721542	Mm_Adamts9_8
secreted	101401	Adamts9	a disintegrin-like and metallopeptidase (reprolysin type) with thrombospo	NM_175314	ACCAGGGAGTCTGATCAGCTA	SI04721535	Mm_Adamts9_7
secreted	67610	Rspry1	ring finger and SPRY domain containing 1	NM_026274	AAGGGTCTCCTGAATACTGAA	SI00842296	Mm_4930470D19Rik_4
secreted	67610	Rspry1	ring finger and SPRY domain containing 1	NM_026274	CTGCAAGACTTGAATCTATAA	SI00842289	Mm_4930470D19Rik_3
secreted	75458	Cklf	chemokine-like factor	NM_001037841 N	CTGCTGTACTCTGAAATGCTT	SI02744735	Mm_Cklf_9
secreted	75458	Cklf	chemokine-like factor	NM_001037841 N	CCCTTCTGCTGTACTCTGAAA	SI02719584	Mm_Cklf_8
secreted	208990	Npb	neuropeptide B	NM_153288	AGCCTTGCCCTGTGTGTCAAA	SI01310428	Mm_MGI:2387153_4
secreted	208990	Npb	neuropeptide B	NM_153288	CAGCGGCAACTCAACAGCCGA	SI01310421	Mm_MGI:2387153_3
secreted	17319	Mif	macrophage migration inhibitory factor	NM_010798	CAGCCTGCACAGCATCGGCAA	SI01312668	Mm_Mif_4
secreted	17319	Mif	macrophage migration inhibitory factor	NM_010798	CACCGGCAAGCCCGCACAGTA	SI01312661	Mm_Mif_3
secreted	23892	Grem1	gremlin 1	NM_011824	ATCGACTTGGATTAAGTCAAA	SI01058169	Mm_Grem1_3
secreted	23892	Grem1	gremlin 1	NM_011824	TAAGAACCAAGTGAAATTAAA	SI01058176	Mm_Grem1_4
receptors	13639	Efna4	ephrin A4	NM_007910	CTGGAATTGCTCGATGCCTTT	SI00990528	Mm_Efna4_4
receptors	13639	Efna4	ephrin A4	NM_007910	AACCAGGCAGACAGGATGAAA	SI00990521	Mm_Efna4_3
secreted	319146	Ifnz	interferon zeta	NM_197889	ACCCAGCCTAGCCAAACCAAA	SI01310848	Mm_MGI:2448469_4
secreted	319146	Ifnz	interferon zeta	NM_197889	GCGAAAGATGTTTGTAAGAAA	SI01310841	Mm_MGI:2448469_3
secreted	13607	Eda	ectodysplasin-A	NM_001177937 N	AAGGCATATTCTGAAGAGGAA	SI02736587	Mm_Eda_8
secreted	13607	Eda	ectodysplasin-A	NM_001177937 N	TGCCATTAGGTTGCAACTTAA	SI02712143	Mm_Eda_7
secreted	319804	Glt1d1	glycosyltransferase 1 domain containing 1	NM_177005	AGGAATTGCAACAATACCAAA	SI04407186	Mm_Glt1d1_3
secreted	319804	Glt1d1	glycosyltransferase 1 domain containing 1	NM_177005	AGAGACGAACTTGGAGGATTA	SI04407193	Mm_Glt1d1_4
secreted	55938	Apom	apolipoprotein M	NM_018816	ACCCGTGTCCTAGACAGACAA	SI00901117	Mm_Apom_3
secreted	55938	Apom	apolipoprotein M	NM_018816	TACCGATTGACTGAAGGGAAA	SI00901124	Mm_Apom_4
receptors	13857	Epor	erythropoietin receptor	NM_010149	CACCTACTTGGTATTGGATAA	SI00190533	Mm_Epor_4
receptors	13857	Epor	erythropoietin receptor	NM_010149	CTGTCTGTATATAGAAATATA	SI02669982	Mm_Epor_5
secreted	14735	Gpc4	glypican 4	NM_008150	CAAAGTAAAGATGATTTCAAA	SI02732863	Mm_Gpc4_7
secreted	14735	Gpc4	glypican 4	NM_008150	TACGTATTTCTGAAATATTAA	SI02708727	Mm_Gpc4_6
secreted	78935	Saal1	serum amyloid A-like 1	NM_030233	ATCCTTGTGTATTTCATACAA	SI02775871	Mm_Saal1_4
secreted	78935	Saal1	serum amyloid A-like 1	NM_030233	AGGGAAGGTGTAACTAACTTA	SI02772238	Mm_Saal1_3
secreted	18610	Pdyn	prodynorphin	NM_018863	CCTCCTCGTGATGCCCTCTAA	SI04417966	Mm_Pdyn_8
secreted	18610	Pdyn	prodynorphin	NM_018863	CAGCAGCTTTGGCAACGGAAA	SI04417959	Mm_Pdyn_7
receptors	14588	Gfra4	glial cell line derived neurotrophic factor family receptor alpha 4	NM_001136063 N	TGCGAGCTTTACCGACGGGAA	SI01011388	Mm_Gfra4_4
receptors	14588	Gfra4	glial cell line derived neurotrophic factor family receptor alpha 4	NM_001136063 N	TGCTCTGATTAGGAACATGAA	SI01011381	Mm_Gfra4_3
secreted	68797	Pdgfrl	platelet-derived growth factor receptor-like	NM_026840	CACAGGGAGTTTCCCGCCAAA	SI04777297	Mm_Pdgfrl_7
secreted	68797	Pdgfrl	platelet-derived growth factor receptor-like	NM_026840	CAGAGTGTCATTATCGTGGAA	SI02744203	Mm_Pdgfrl_6
secreted	16183	Il2	interleukin 2	NM_008366	CAGCACAAAGTAAGCGCTAAA	SI00174930	Mm_Il2_3
secreted	16183	Il2	interleukin 2	NM_008366	CAGGATGGAGAATTACAGGAA	SI00174937	Mm_Il2_4
secreted	14165	Fgf10	fibroblast growth factor 10	NM_008002	CAGAGGGACCCTTACCTTAAA	SI02708433	Mm_Fgf10_7
secreted	14165	Fgf10	fibroblast growth factor 10	NM_008002	AACTTCTGAGATAGAACTTAA	SI02732520	Mm_Fgf10_8
secreted	14130	Fcgr2b	Fc receptor, IgG, low affinity IIb	NM_001077189 N	ATGATGCTTCTTTATAAGGAA	SI01001273	Mm_Fcgr2b_3
secreted	14130	Fcgr2b	Fc receptor, IgG, low affinity IIb	NM_001077189 N	CACGGTGACACTGACATGCGA	SI01001280	Mm_Fcgr2b_4
secreted	17242	Mdk	midkine	NM_001012335 N	CACCTCCAAGACCAAGTCAAA	SI01303029	Mm_Mdk_3
secreted	17242	Mdk	midkine	NM_001012335 N	CAAGACCAAGTCAAAGACCAA	SI01303036	Mm_Mdk_4
secreted	108153	Adamts7	a disintegrin-like and metallopeptidase (reprolysin type) with thrombospo	NM_001003911	AACGCTATTGATGAGGAGGAA	SI00889812	Mm_Adamts7_4
secreted	108153	Adamts7	a disintegrin-like and metallopeptidase (reprolysin type) with thrombospo	NM_001003911	CAGCGGGTAGCCAGACGGTAA	SI00889805	Mm_Adamts7_3
receptors	14183	Fgfr2	fibroblast growth factor receptor 2	NM_010207 NM_	CAGAAACGTGTTGGTAACAGA	SI01002680	Mm_Fgfr2_4
receptors	14183	Fgfr2	fibroblast growth factor receptor 2	NM_010207 NM_	TTGGGAAACATAAGAACATTA	SI01002673	Mm_Fgfr2_3
receptors	12931	Crlf1	cytokine receptor-like factor 1	NM_018827	TACCATCTGGGCAACAAAGAA	SI02693768	Mm_Crlf1_4
receptors	12931	Crlf1	cytokine receptor-like factor 1	NM_018827	ACGCATACTGCTCGAACCTTA	SI02716553	Mm_Crlf1_5
secreted	16169	Il15ra	interleukin 15 receptor, alpha chain	NM_008358 NM_	TGGCCTGGTACATCAAATCAA	SI02733178	Mm_Il15ra_11
secreted	16169	Il15ra	interleukin 15 receptor, alpha chain	NM_008358 NM_	AAGGAGGATGAAGACACAGGA	SI02709077	Mm_Il15ra_10
secreted	16005	Igfals	insulin-like growth factor binding protein, acid labile subunit	NM_008340	CACCCTGCTGGCAACAATTAA	SI02709014	Mm_Igfals_7
secreted	16005	Igfals	insulin-like growth factor binding protein, acid labile subunit	NM_008340	CTGGTGCTTGCTGGCAACAAA	SI02687132	Mm_Igfals_6
secreted	16997	Ltbp2	latent transforming growth factor beta binding protein 2	NM_013589	CCCAGGATGGACAACATCAAA	SI02740038	Mm_Ltbp2_8
secreted	16997	Ltbp2	latent transforming growth factor beta binding protein 2	NM_013589	CTGAGTGGTAAGAAATACAAA	SI02715307	Mm_Ltbp2_7
receptors	54393	Gabbr1	gamma-aminobutyric acid (GABA) B receptor, 1	NM_019439	CCGAATCTGCTCCAAGTCTTA	SI02741452	Mm_Gabbr1_8
receptors	54393	Gabbr1	gamma-aminobutyric acid (GABA) B receptor, 1	NM_019439	AAGGATCTCCCTGAATCTCAA	SI02716665	Mm_Gabbr1_7
secreted	56788	Scube2	signal peptide, CUB domain, EGF-like 2	NM_020052	ATGGACCTCGATATGGCTAAA	SI05349778	Mm_Scube2_5
secreted	56788	Scube2	signal peptide, CUB domain, EGF-like 2	NM_020052	CACCAACATAACACAGTGTAA	SI01412096	Mm_Scube2_4
secreted	242316	Gdf6	growth differentiation factor 6	NM_013526	AAGGGTCTTACCCTTAGAGAA	SI01010940	Mm_Gdf6_4
secreted	242316	Gdf6	growth differentiation factor 6	NM_013526	AAGGAATAAGTGAGTCAGGAA	SI01010933	Mm_Gdf6_3
secreted	68119	Cmtm3	CKLF-like MARVEL transmembrane domain containing 3	NM_024217	CTCCCGAGGCCTCACATTCAA	SI00952000	Mm_Cklfsf3_4
secreted	68119	Cmtm3	CKLF-like MARVEL transmembrane domain containing 3	NM_024217	CCCACCGAAGCACACATCAAA	SI00951993	Mm_Cklfsf3_3
secreted	226519	Lamc1	laminin, gamma 1	NM_010683	CAGGACTTAATGCAGAAGAAA	SI02737511	Mm_Lamc1_8
secreted	226519	Lamc1	laminin, gamma 1	NM_010683	CAGGCCTTGTGTACTGATGAA	SI02712983	Mm_Lamc1_7
secreted	13019	Ctf1	cardiotrophin 1	NM_007795	CAGAGGGAGGGAAGTCTGGAA	SI00961940	Mm_Ctf1_4
secreted	13019	Ctf1	cardiotrophin 1	NM_007795	CTGGAGGAATACGTGCAGCAA	SI00961933	Mm_Ctf1_3
receptors	58217	Trem1	triggering receptor expressed on myeloid cells 1	NM_021406	CAGTTTGGACTTTGTTGTGAA	SI01455076	Mm_Trem1_4
receptors	58217	Trem1	triggering receptor expressed on myeloid cells 1	NM_021406	CACACACACGTTGTATGATAA	SI01455069	Mm_Trem1_3
secreted	57266	Cxcl14	chemokine (C-X-C motif) ligand 14	NM_019568	CAGGTTTCATGTTCTTTCTAA	SI00213983	Mm_Cxcl14_4
secreted	57266	Cxcl14	chemokine (C-X-C motif) ligand 14	NM_019568	GTGGACGATCATGGAAAGAAA	SI00213976	Mm_Cxcl14_3
secreted	268935	Scube3	signal peptide, CUB domain, EGF-like 3	NM_001004366	CGCCTGCATCTGCGAAACAAA	SI01412117	Mm_Scube3_3
secreted	268935	Scube3	signal peptide, CUB domain, EGF-like 3	NM_001004366	TACAAGCTTCTCATCAACGAA	SI01412124	Mm_Scube3_4
secreted	15289	Hmgb1	high mobility group box 1	NM_010439	TGCGTTGTTAATGTAATTTGA	SI01067724	Mm_Hmgb1_4
secreted	15289	Hmgb1	high mobility group box 1	NM_010439	TGGATAAGAGATATTATATAA	SI01067717	Mm_Hmgb1_3
secreted	83961	Nrg4	neuregulin 4	NM_032002	AAGGGCATCATTACCTACAAA	SI01331092	Mm_Nrg4_4
secreted	83961	Nrg4	neuregulin 4	NM_032002	CAGCCTGGTAGAGACAAACAA	SI01331085	Mm_Nrg4_3
secreted	100102	Pcsk9	proprotein convertase subtilisin/kexin type 9	NM_153565	AGGCCGAAACCTGATCCTTTA	SI01373036	Mm_Pcsk9_4
secreted	100102	Pcsk9	proprotein convertase subtilisin/kexin type 9	NM_153565	CTGCGTGTGCTCAACTGTCAA	SI01373029	Mm_Pcsk9_3
secreted	67666	Hapln3	hyaluronan and proteoglycan link protein 3	NM_178255	ACGGAAGTGCCCGCTACCCTA	SI01062992	Mm_Hapln3_4
secreted	67666	Hapln3	hyaluronan and proteoglycan link protein 3	NM_178255	GTGGTGGAAGTTGTCAGAGAA	SI01062985	Mm_Hapln3_3
secreted	14114	Fbln1	fibulin 1	NM_010180	CACCGGAACGTCGTCAATGTA	SI00999628	Mm_Fbln1_4
secreted	14114	Fbln1	fibulin 1	NM_010180	CAGGAGTGTGCTAATGTCTAT	SI00999621	Mm_Fbln1_3
receptors	13805	Eng	endoglin	NM_001146348 N	CCGGACGGTGACCGTAATGAT	SI00993832	Mm_Eng_4
receptors	13805	Eng	endoglin	NM_001146348 N	CCCATGCTGCTTAGAAGCCTA	SI00993825	Mm_Eng_3
secreted	15974	Ifnab	interferon alpha B	NM_008336	CAGCAGCATTGGCAATATTCA	SI01073856	Mm_Ifnab_4
secreted	15974	Ifnab	interferon alpha B	NM_008336	TGGGAAGTGGTCAGAGCAGAA	SI01073849	Mm_Ifnab_3
receptors	15975	Ifnar1	interferon (alpha and beta) receptor 1	NM_010508	CTGAATGTCAACATACTACAA	SI02737224	Mm_Ifnar1_7
receptors	15975	Ifnar1	interferon (alpha and beta) receptor 1	NM_010508	CAGGCACATGTTCTTAAGGAA	SI02690408	Mm_Ifnar1_6
secreted	18121	Nog	noggin	NM_008711	AAGGCAAGAAACAGCGCCTGA	SI01328964	Mm_Nog_4
secreted	18121	Nog	noggin	NM_008711	GCCCGAGGGCATGGTGTGTAA	SI01328957	Mm_Nog_3

secreted	19131	Prh1	proline rich protein HaeIII subfamily 1	NM_011174	CAGGCCCACAACCAAGACCTA	SI01388072	Mm_Prh1_4
secreted	19131	Prh1	proline rich protein HaeIII subfamily 1	NM_011174	CAAATCCAGATCCCAAATCAA	SI01388065	Mm_Prh1_3
secreted	72709	C1qtnf6	C1q and tumor necrosis factor related protein 6	NM_028331	CTGGAACTACAAGGAGACCTA	SI04408999	Mm_C1qtnf6_6
secreted	72709	C1qtnf6	C1q and tumor necrosis factor related protein 6	NM_028331	CTCCATGACATTGACGCTACA	SI04408992	Mm_C1qtnf6_5
secreted	14183	Fgfr2	fibroblast growth factor receptor 2	NM_010207 NM_	CAGAAACGTGTTGGTAACAGA	SI01002680	Mm_Fgfr2_4
secreted	14183	Fgfr2	fibroblast growth factor receptor 2	NM_010207 NM_	TTGGGAAACATAAGAACATTA	SI01002673	Mm_Fgfr2_3
secreted	12153	Bmp1	bone morphogenetic protein 1	NM_009755 NR_(	CCGCATGTTCTTGCGCTTCTA	SI02735880	Mm_Bmp1_8
secreted	12153	Bmp1	bone morphogenetic protein 1	NM_009755 NR_(	CCGGAGCTCCTCAACTACAAA	SI02711534	Mm_Bmp1_7
secreted	12064	Bdnf	brain derived neurotrophic factor	NM_001048139 N	CACAGCAATAATGATATTAAT	SI02707628	Mm_Bdnf_7
secreted	12064	Bdnf	brain derived neurotrophic factor	NM_001048139 N	CAGGGTAAATTATTCAGTTAA	SI02685928	Mm_Bdnf_6
receptors	16169	Il15ra	interleukin 15 receptor, alpha chain	NM_008358 NM_	TGGCCTGGTACATCAAATCAA	SI02733178	Mm_Il15ra_11
receptors	16169	Il15ra	interleukin 15 receptor, alpha chain	NM_008358 NM_	AAGGAGGATGAAGACACAGGA	SI02709077	Mm_Il15ra_10
secreted	22409	Wnt10a	wingless related MMTV integration site 10a	NM_009518	CCGGATGTGGGCTTCGGAGAA	SI01472576	Mm_Wnt10a_4
secreted	22409	Wnt10a	wingless related MMTV integration site 10a	NM_009518	CACGCTCATCCGGCCGCACAA	SI01472569	Mm_Wnt10a_3
secreted	215615	Rnpep	arginyl aminopeptidase (aminopeptidase B)	NM_001159624 N	CAGGATCAGTTTGACAAGTTT	SI02677017	Mm_Rnpep_5
secreted	215615	Rnpep	arginyl aminopeptidase (aminopeptidase B)	NM_001159624 N	AAGAATGACTACCAAGAGGAA	SI00236775	Mm_Rnpep_4
secreted	217194	Klhl11	kelch-like 11 (Drosophila)	NM_172565	CGGAAGGAGGAGACTACTTAA	SI01083880	Mm_Klhl11_4
secreted	217194	Klhl11	kelch-like 11 (Drosophila)	NM_172565	CTGGTAAATAAGATCTTATTA	SI01083873	Mm_Klhl11_3
secreted	30933	Tor2a	torsin family 2, member A	NM_152800	CCGCACTGAGCAGTACAAGAA	SI01453816	Mm_Tor2a_4
secreted	30933	Tor2a	torsin family 2, member A	NM_152800	CTGGTCAGAGCAAACACGAAA	SI01453809	Mm_Tor2a_3
secreted	15200	Hbegf	heparin-binding EGF-like growth factor	NM_010415	TTGGTCATTGATACCTATCAA	SI02737147	Mm_Dtr_6
secreted	15200	Hbegf	heparin-binding EGF-like growth factor	NM_010415	TGGGTCCTATTTGCTCTGTAA	SI02712633	Mm_Dtr_5
secreted	260301	Otos	otospiralin	NM_153114	CTGGAACTACGTGCAATATTT	SI01367324	Mm_Otos_4
secreted	260301	Otos	otospiralin	NM_153114	GTGGAGCACATGAGCAATAAA	SI01367317	Mm_Otos_3
secreted	12653	Chgb	chromogranin B	NM_007694	TTGTTGAAACACAAACAATAA	SI00950376	Mm_Chgb_4
secreted	12653	Chgb	chromogranin B	NM_007694	CACCAGGAGGCAAACGATGAA	SI00950369	Mm_Chgb_3
secreted	17388	Mmp15	matrix metallopeptidase 15	NM_008609	CAGGAAAGGCATGGAACAATT	SI02733829	Mm_Mmp15_5
secreted	17388	Mmp15	matrix metallopeptidase 15	NM_008609	AAGAACTGTGTTTGCCTTGAA	SI00177835	Mm_Mmp15_3
secreted	14751	Gpi1	glucose phosphate isomerase 1	NM_008155	AAGGGTACACTGGCAAATCCA	SI02732870	Mm_Gpi1_6
secreted	14751	Gpi1	glucose phosphate isomerase 1	NM_008155	CCGTGTCTGGTTTGTCTCTAA	SI02667154	Mm_Gpi1_5
secreted	64817	Svep1	sushi, von Willebrand factor type A, EGF and pentraxin domain containing	NM_022814	CCGAGTGAAGGGAGAAGAATA	SI01308349	Mm_MGI:1928849_3
secreted	64817	Svep1	sushi, von Willebrand factor type A, EGF and pentraxin domain containing	NM_022814	CAGATCTGATTTGTACGGAAA	SI01308356	Mm_MGI:1928849_4
secreted	20210	Saa3	serum amyloid A 3	NM_011315	CTCTGACATGAAGAAAGCTAA	SI04419555	Mm_Saa3_6
secreted	20210	Saa3	serum amyloid A 3	NM_011315	AAGAAGCTGGTCAAGGGTCTA	SI04419548	Mm_Saa3_5
secreted	11603	Agrn	agrin	NM_021604	CCCGGAGACCTGCCAGTTTAA	SI00891660	Mm_Agrn_4
secreted	11603	Agrn	agrin	NM_021604	CTGGAGGACGCTGTCACCAAA	SI00891653	Mm_Agrn_3
receptors	85030	Tnfrsf25	tumor necrosis factor receptor superfamily, member 25	NM_033042	AGCCTTAAGTATTGTTACTTA	SI01452528	Mm_Tnfrsf25_4
receptors	85030	Tnfrsf25	tumor necrosis factor receptor superfamily, member 25	NM_033042	CACCTTCTTGACCAGAGACAA	SI01452521	Mm_Tnfrsf25_3
secreted	69675	Pxdn	peroxidasin homolog (Drosophila)	NM_181395	CAGCAGAGCTGTTGAAGCTAA	SI00815444	Mm_2310075M15Rik_4
secreted	69675	Pxdn	peroxidasin homolog (Drosophila)	NM_181395	CCCTGTGTTTGTAGAAGGAAA	SI00815437	Mm_2310075M15Rik_3
secreted	19773	Rln1	relaxin 1	NM_011272	CCAGCTCTGCTTGCCAGGCAA	SI04953536	Mm_Rln1_6
secreted	19773	Rln1	relaxin 1	NM_011272	CAGAAGCAGCCCAGACCGGAA	SI04953529	Mm_Rln1_5
secreted	12834	Col6a2	collagen, type VI, alpha 2	NM_146007	CCCGATGGATACCCTGGAGAA	SI00956284	Mm_Col6a2_4
secreted	12834	Col6a2	collagen, type VI, alpha 2	NM_146007	CCGCATGGCTCTGTTGCAATA	SI00956277	Mm_Col6a2_3
secreted	268977	Ltbp1	latent transforming growth factor beta binding protein 1	NM_019919 NM_	CACTGTGAGGATATCAATGAA	SI02742047	Mm_Ltbp1_8
secreted	268977	Ltbp1	latent transforming growth factor beta binding protein 1	NM_019919 NM_	CTGGGTATGAATGCTACTGTA	SI02717190	Mm_Ltbp1_7
secreted	23837	Cfdp1	craniofacial development protein 1	NM_011801	TCCGGTGGAGAGTACAGTGAA	SI00949312	Mm_Cfdp1_4
secreted	23837	Cfdp1	craniofacial development protein 1	NM_011801	AAGGGTGACTAAGGAAGTAGA	SI00949305	Mm_Cfdp1_3
secreted	14562	Gdf3	growth differentiation factor 3	NM_008108	ACCATATTATATCCAATTGTA	SI02708643	Mm_Gdf3_6
secreted	14562	Gdf3	growth differentiation factor 3	NM_008108	CTCAATGACCACGTATTTAAA	SI02686796	Mm_Gdf3_5
secreted	71768	Vwce	von Willebrand factor C and EGF domains	NM_027913	AAGGAGAACTCTACTATCTGA	SI00790132	Mm_1300015B04Rik_4
secreted	71768	Vwce	von Willebrand factor C and EGF domains	NM_027913	TAGAGTAAGATGGTTCATCTA	SI00790125	Mm_1300015B04Rik_3
receptors	15976	Ifnar2	interferon (alpha and beta) receptor 2	NM_001110498 N	TACCACGAAGTCATCATGTGA	SI04947866	Mm_Ifnar2_10
receptors	15976	Ifnar2	interferon (alpha and beta) receptor 2	NM_001110498 N	CACAGTCATGAGCAAAGACGA	SI04947859	Mm_Ifnar2_9
secreted	70726	Angptl6	angiopoietin-like 6	NM_145154	CAGAGACTCATATTCTGGTAA	SI00897932	Mm_Angptl6_4
secreted	70726	Angptl6	angiopoietin-like 6	NM_145154	CTCTCTTGGCACAATGACAAA	SI00897925	Mm_Angptl6_3
secreted	13380	Dkk1	dickkopf homolog 1 (Xenopus laevis)	NM_010051	AAAGTAGTTAACAACCAATTA	SI02712045	Mm_Dkk1_5
secreted	13380	Dkk1	dickkopf homolog 1 (Xenopus laevis)	NM_010051	ATCATAAATTCTAGAATGTAA	SI00979608	Mm_Dkk1_4
secreted	15968	Ifna5	interferon alpha 5	NM_010505	CTCAGGAACAAGAGAGCCTTA	SI01073744	Mm_Ifna5_4
secreted	15968	Ifna5	interferon alpha 5	NM_010505	CTCCTGCCTGAAGGACAGAAA	SI01073737	Mm_Ifna5_3
secreted	69583	Tnfsf13	tumor necrosis factor (ligand) superfamily, member 13	NM_001159505 N	TCCAGTTAACATTACCTCCAA	SI02856364	Mm_Tnfsf13_4
secreted	69583	Tnfsf13	tumor necrosis factor (ligand) superfamily, member 13	NM_001159505 N	CACCAAATTCTCCTGAGGCTA	SI02856357	Mm_Tnfsf13_3
secreted	12826	Col4a1	collagen, type IV, alpha 1	NM_009931	AACGTGCAATTACTACGCAAA	SI04715382	Mm_Col4a1_6
secreted	12826	Col4a1	collagen, type IV, alpha 1	NM_009931	CGCCCTGGATTTAACGGCTTA	SI04458223	Mm_Col4a1_5
receptors	12978	Csf1r	colony stimulating factor 1 receptor	NM_001037859	AAGGCTGACCACACCAACAAA	SI02708076	Mm_Csf1r_7
receptors	12978	Csf1r	colony stimulating factor 1 receptor	NM_001037859	TTCCAAGATAACTATTATAAA	SI02732107	Mm_Csf1r_8
secreted	17087	Ly96	lymphocyte antigen 96	NM_001159711 N	TCCCATATTGACTGAATCTGA	SI01297604	Mm_Ly96_4
secreted	17087	Ly96	lymphocyte antigen 96	NM_001159711 N	CGAGGGAATACTATTTCCTAA	SI01297597	Mm_Ly96_3
secreted	77041	Arsk	arylsulfatase K	NM_029847	CTCATTCATATACAATTTAAA	SI00827232	Mm_2810429K17Rik_4
secreted	77041	Arsk	arylsulfatase K	NM_029847	ATCAGCCTTAACAATTATTAA	SI00827225	Mm_2810429K17Rik_3
secreted	50722	Dkkl1	dickkopf-like 1	NM_015789	CTGGATATTAACTGTCCGTAT	SI00979720	Mm_Dkkl1_4
secreted	50722	Dkkl1	dickkopf-like 1	NM_015789	CTGGAAGGTTCCCAAAGTAGA	SI00979713	Mm_Dkkl1_3
secreted	20442	St3gal1	ST3 beta-galactoside alpha-2,3-sialyltransferase 1	NM_009177	CTCCTTACAGATGTTCAATAA	SI01417997	Mm_Siat4a_3
secreted	20442	St3gal1	ST3 beta-galactoside alpha-2,3-sialyltransferase 1	NM_009177	AACGGTGGGCTCTTAACTGAA	SI01418004	Mm_Siat4a_4
secreted	234199	Fgl1	fibrinogen-like protein 1	NM_145594	CTGGAGATTCCCTGTCAGGAA	SI02697779	Mm_Fgl1_6
secreted	234199	Fgl1	fibrinogen-like protein 1	NM_145594	CAGCTCTTGCATGAGAAGGAA	SI02746681	Mm_Fgl1_7
secreted	170458	Gpha2	glycoprotein hormone alpha 2	NM_130453	CAAGGGCCTGCCAGTGTGATA	SI01055544	Mm_Gpha2_4
secreted	170458	Gpha2	glycoprotein hormone alpha 2	NM_130453	ACCCTCTTTATCGTCTGTGAA	SI01055537	Mm_Gpha2_3
secreted	79202	Tnfrsf22	tumor necrosis factor receptor superfamily, member 22	NM_023680	ACCTGTGATAAAGATCAGGAA	SI01452472	Mm_Tnfrsf22_4
secreted	79202	Tnfrsf22	tumor necrosis factor receptor superfamily, member 22	NM_023680	CAGACTAAGACAACTCTAATA	SI01452465	Mm_Tnfrsf22_3
secreted	14061	F2	coagulation factor II	NM_010168	CTGGAACAGCTTACCAGCCAA	SI02690030	Mm_F2_6
secreted	14061	F2	coagulation factor II	NM_010168	CAGAAAGTCATTGATCAATTT	SI02712227	Mm_F2_7
secreted	15945	Cxcl10	chemokine (C-X-C motif) ligand 10	NM_021274	AAGACAATGTACTGTATTGAA	SI00962808	Mm_Cxcl10_4
secreted	15945	Cxcl10	chemokine (C-X-C motif) ligand 10	NM_021274	CTGGGTCTGAGTGGGACTCAA	SI00962801	Mm_Cxcl10_3
secreted	22418	Wnt5a	wingless-related MMTV integration site 5A	NM_009524	CAGGTTGTTATAGAAGCTAAT	SI02735516	Mm_Wnt5a_5
secreted	22418	Wnt5a	wingless-related MMTV integration site 5A	NM_009524	CAGCTGATTCTTAATACCAGA	SI01472793	Mm_Wnt5a_3
secreted	23863	Dand5	DAN domain family, member 5	NM_201227 NR_(	AAGCAGGAGACGCAATAGAAA	SI00974316	Mm_Dand5_4
secreted	23863	Dand5	DAN domain family, member 5	NM_201227 NR_(	CTGGGTTCTATTCGTATGTGA	SI00974309	Mm_Dand5_3
secreted	67703	Kirrel3	kin of IRRE like 3 (Drosophila)	NM_001190911 N	CTGCTTCGAGACGGCAAACGA	SI01083236	Mm_Kirrel3_4
secreted	67703	Kirrel3	kin of IRRE like 3 (Drosophila)	NM_001190911 N	AAAGGTGTTGTATCAGCCAAA	SI01083229	Mm_Kirrel3_3
secreted	109267	Srcrb4d	scavenger receptor cysteine rich domain containing, group B (4 domains)	NM_001160366	CTGGACAATGTTCACTGCGAA	SI01432984	Mm_Srcrb4d_4
secreted	109267	Srcrb4d	scavenger receptor cysteine rich domain containing, group B (4 domains)	NM_001160366	CCAGGATGCCTTCCTATGATA	SI04393536	Mm_Srcrb4d_6
secreted	22157	Tulp1	tubby like protein 1	NM_021478	CGCAGTGATGAAGAACAGCAA	SI02694790	Mm_Tulp1_6
secreted	22157	Tulp1	tubby like protein 1	NM_021478	AAGGGCAGAGCTAAAGGGAAA	SI02674294	Mm_Tulp1_5
secreted	64075	Smoc1	SPARC related modular calcium binding 1	NM_001146217 N	TTGGCAGTCTACAGTGAATAA	SI01426880	Mm_Smoc1_4
secreted	64075	Smoc1	SPARC related modular calcium binding 1	NM_001146217 N	AACGTAAGAAATTCAGAGAAA	SI01426873	Mm_Smoc1_3
secreted	76486	Ly6k	lymphocyte antigen 6 complex, locus K	NM_029627	ACGTTTCTTCTATGTGTCGAA	SI00817040	Mm_2410015A16Rik_4
secreted	76486	Ly6k	lymphocyte antigen 6 complex, locus K	NM_029627	CAGTGGGAGAAGACATAGATA	SI00817033	Mm_2410015A16Rik_3
secreted	16160	Il12b	interleukin 12b	NM_008352	AGAAGTGATATTGATAAGAAA	SI02733157	Mm_Il12b_7
secreted	16160	Il12b	interleukin 12b	NM_008352	TTGAAATATTTAAGTAATTTA	SI02667483	Mm_Il12b_5
receptors	11990	Atrn	attractin	NM_009730	CTGGCTCTTCTGGATTTGTAA	SI00908152	Mm_Atrn_4
receptors	11990	Atrn	attractin	NM_009730	AACCAACATCAAGGAATACAA	SI00908145	Mm_Atrn_3
secreted	21944	Tnfsf12	tumor necrosis factor (ligand) superfamily, member 12	NM_011614	AAATGTTAAATGGATATTAAA	SI02671620	Mm_Tnfsf12_5

secreted	21944	Tnfsf12	tumor necrosis factor (ligand) superfamily, member 12	NM_011614	CAGGTGTGGATGGGACAGTGA	SI00201950	Mm_Tnfsf12_4
secreted	18591	Pdgfb	platelet derived growth factor, B polypeptide	NM_011057	AAGGCAAGCACCGAAAGTTTA	SI01373785	Mm_Pdgfb_3
secreted	18591	Pdgfb	platelet derived growth factor, B polypeptide	NM_011057	CACACCTTCTCTGATGGATTA	SI01373792	Mm_Pdgfb_4
secreted	54612	Sfrp5	secreted frizzled-related sequence protein 5	NM_018780	AAGGACTACGGATACAGGATA	SI00212282	Mm_Sfrp5_4
secreted	54612	Sfrp5	secreted frizzled-related sequence protein 5	NM_018780	CTCCAGTGACTTTGTGGTCAA	SI02693698	Mm_Sfrp5_5
secreted	12854	Cort	cortistatin	NM_007745	CCCTGTGAGATGCCAACGAGA	SI02732016	Mm_Cort_4
secreted	12854	Cort	cortistatin	NM_007745	CCGAGGATGATGGGTGGCCGA	SI02707964	Mm_Cort_3
secreted	94352	Loxl2	lysyl oxidase-like 2	NM_033325	CGCGTGGAGGTCTACTACGAA	SI05170095	Mm_Loxl2_7
secreted	94352	Loxl2	lysyl oxidase-like 2	NM_033325	TAGCCACGACTTAAAGTACAA	SI04568375	Mm_LOC100047339_4
receptors	17087	Ly96	lymphocyte antigen 96	NM_001159711 N	CGAGGGAATACTATTTCCTAA	SI01297597	Mm_Ly96_3
receptors	17087	Ly96	lymphocyte antigen 96	NM_001159711 N	TCCCATATTGACTGAATCTGA	SI01297604	Mm_Ly96_4
secreted	26565	Pla2g10	phospholipase A2, group X	NM_011987	CAGCCCTAAGTTAGACCGCTA	SI02714943	Mm_Pla2g10_6
secreted	26565	Pla2g10	phospholipase A2, group X	NM_011987	CAGGAGGTCACATGTATACAA	SI02692382	Mm_Pla2g10_5
secreted	12163	Bmp8a	bone morphogenetic protein 8a	NM_007558	AAACCCTTCTATGTTATCATA	SI02731694	Mm_Bmp8a_7
secreted	12163	Bmp8a	bone morphogenetic protein 8a	NM_007558	CACCTCTGTGCTGTACTATGA	SI02685956	Mm_Bmp8a_6
receptors	18053	Ngfr	nerve growth factor receptor (TNFR superfamily, member 16)	NM_033217	AACATACGTGATGACCATTAA	SI02676023	Mm_Ngfr_3
receptors	18053	Ngfr	nerve growth factor receptor (TNFR superfamily, member 16)	NM_033217	ATGGAGTGACTTTCAGGGAAA	SI02745309	Mm_Ngfr_6
secreted	24111	Uts2	urotensin 2	NM_011910	CCCGTTGGTCTCTCAGAACCA	SI05170970	Mm_Uts2_5
secreted	24111	Uts2	urotensin 2	NM_011910	CCCAGAGTGCTTCTGGAAATA	SI01464260	Mm_Uts2_4
receptors	17480	Mpl	myeloproliferative leukemia virus oncogene	NM_001122949 N	CTCCAGATGGCTGCTCACAAA	SI02713214	Mm_Mpl_6
receptors	17480	Mpl	myeloproliferative leukemia virus oncogene	NM_001122949 N	TAGACACTGTTTATTATATAA	SI02670703	Mm_Mpl_5
secreted	12847	Copa	coatomer protein complex subunit alpha	NM_009938	TTGGGCATTTAATGTCATAAA	SI00956844	Mm_Copa_4
secreted	12847	Copa	coatomer protein complex subunit alpha	NM_009938	TGGTGGGATGATTGTATTTAA	SI00956837	Mm_Copa_3
secreted	17082	Il1rl1	interleukin 1 receptor-like 1	NM_001025602 N	CAGCACCTGTTCAGTGGTTTA	SI02713067	Mm_Il1rl1_7
secreted	17082	Il1rl1	interleukin 1 receptor-like 1	NM_001025602 N	AACGTGACTCATGATGATGAA	SI02737616	Mm_Il1rl1_8
secreted	81701	Egfl8	EGF-like domain 8	NM_152922	TGCCACGTGGATGTCGATGAA	SI00990780	Mm_Egfl8_4
secreted	81701	Egfl8	EGF-like domain 8	NM_152922	GAGGAGGATCTTTCAAAGAGA	SI00990773	Mm_Egfl8_3
secreted	20302	Ccl3	chemokine (C-C motif) ligand 3	NM_011337	CACACTGTTTGGTGACAGCTA	SI00943376	Mm_Ccl3_4
secreted	20302	Ccl3	chemokine (C-C motif) ligand 3	NM_011337	TCGAGGGACTCTTCACTTGAA	SI00943369	Mm_Ccl3_3
receptors	12982	Csf2ra	colony stimulating factor 2 receptor, alpha, low-affinity (granulocyte-mac	NM_009970	TCAGTTCTTAACAAACCCTAA	SI00960540	Mm_Csf2ra_4
receptors	12982	Csf2ra	colony stimulating factor 2 receptor, alpha, low-affinity (granulocyte-mac	NM_009970	ACCCTGGAAGGGCATGTTTAA	SI00960533	Mm_Csf2ra_3
secreted	380780	Serpina11	serine (or cysteine) peptidase inhibitor, clade A (alpha-1 antiproteinase, a	NM_001166350 N	TTGCGTCTATACAAGCAACTA	SI05348749	Mm_Serpina11_5
secreted	380780	Serpina11	serine (or cysteine) peptidase inhibitor, clade A (alpha-1 antiproteinase, a	NM_001166350 N	AAGCTGACTTATCAGGAATTA	SI01414672	Mm_Serpina11_4
secreted	12631	Cfl1	cofilin 1, non-muscle	NM_007687	TTAGTTCTGTGTGTAAATGAA	SI04412135	Mm_Cfl1_8
secreted	12631	Cfl1	cofilin 1, non-muscle	NM_007687	CTCATGGAAGCAGGACCAGTA	SI04412128	Mm_Cfl1_7
secreted	18413	Osm	oncostatin M	NM_001013365	CAGGGCCTAGGTTCCCTGGTA	SI05170361	Mm_Osm_5
secreted	18413	Osm	oncostatin M	NM_001013365	CTGACATAGGGTGGACATCAA	SI01366904	Mm_Osm_4
receptors	18595	Pdgfra	platelet derived growth factor receptor, alpha polypeptide	NM_001083316 N	AACACGTTTCTCAATAAAGTA	SI01373876	Mm_Pdgfra_4
receptors	18595	Pdgfra	platelet derived growth factor receptor, alpha polypeptide	NM_001083316 N	CTCCAGCATTGTAATTATGTA	SI01373869	Mm_Pdgfra_3
secreted	228413	Prrg4	proline rich Gla (G-carboxyglutamic acid) 4 (transmembrane)	NM_178695	CGCTATCATCTTGGTAAGAAA	SI01390172	Mm_Prrg4_4
secreted	228413	Prrg4	proline rich Gla (G-carboxyglutamic acid) 4 (transmembrane)	NM_178695	CAGATACTTGTCATAATCATA	SI01390165	Mm_Prrg4_3
secreted	102545	Cmtm7	CKLF-like MARVEL transmembrane domain containing 7	NM_133978	GAGGCCGAAGACCCTAATTTA	SI00952112	Mm_Cklfsf7_4
secreted	102545	Cmtm7	CKLF-like MARVEL transmembrane domain containing 7	NM_133978	CTGGTCTTGAAGCGTCTCTAA	SI00952105	Mm_Cklfsf7_3
secreted	66659	Acp6	acid phosphatase 6, lysophosphatidic	NM_019800	CTCCACCAACATGTTTCGGAA	SI04923072	Mm_Acp6_10
secreted	66659	Acp6	acid phosphatase 6, lysophosphatidic	NM_019800	CACGGTTTGAAGTGCAAGTTT	SI04923065	Mm_Acp6_9
secreted	13636	Efna1	ephrin A1	NM_001162425 N	CAGTCTGTGGAGTTTAGCTTA	SI05181610	Mm_Efna1_6
secreted	13636	Efna1	ephrin A1	NM_001162425 N	AAGGTGACTGTCAATGGCAAA	SI02712150	Mm_Efna1_5
secreted	20377	Sfrp1	secreted frizzled-related protein 1	NM_013834	CAGGAAGGATTCATAACCAAT	SI01415876	Mm_Sfrp1_4
secreted	20377	Sfrp1	secreted frizzled-related protein 1	NM_013834	CACCTTTGATAGGAAACTGTA	SI01415869	Mm_Sfrp1_3

Table C. Gene profile associated with the effect of Y-27632 and J2 cells on HFKs.
Total RNA was extracted from HFKs grown in medium (F), with J2 cells (J2), Y-27632 (Y) or both J2 cells+ Y-27632 (J2+Y). Shown are the genes whose expression changed >3.0-fold based on the ratio of Y/F, J2/F and J2+Y/F.

Symbol	F	Y	J2	J2+Y	Y/F	J2/F	J2+Y/F
ECM2	5.4	141.9	22.8	657.7	26.0	4.2	120.7
CTH	12.0	294.9	52.0	827.7	24.7	4.4	69.3
UST	10.1	194.4	53.1	463.6	19.2	5.2	45.7
DOCK11	15.5	178.1	336.2	663.2	11.5	21.7	42.7
STC2	30.7	1004.3	69.1	1034.8	32.8	2.3	33.7
PTGS1	21.9	423.5	212.7	731.0	19.3	9.7	33.4
SCN4B	14.5	100.1	97.0	463.2	6.9	6.7	32.0
H19	76.8	1579.6	354.1	1916.6	20.6	4.6	25.0
SLC7A11	473.8	8254.4	2063.1	11269.1	17.4	4.4	23.8
CHAC1	45.0	677.8	149.7	1061.3	15.1	3.3	23.6
TRIB2	17.4	42.7	67.4	410.0	2.5	3.9	23.6
TYMS	21.6	152.3	672.0	486.5	7.0	31.1	22.5
BCHE	34.7	256.3	126.7	739.9	7.4	3.7	21.3
CADM1	19.4	323.1	45.8	378.5	16.7	2.4	19.5
AKR1B10	60.1	1275.7	399.0	1062.0	21.2	6.6	17.7
CEP68	22.5	86.9	86.9	390.9	3.9	3.9	17.4
MPHOSPH9	20.8	101.8	87.7	342.3	4.9	4.2	16.4
SESN2	23.4	370.0	106.5	381.9	15.8	4.5	16.3
NASP	31.8	73.0	278.0	475.3	2.3	8.7	14.9
KIRREL	36.5	214.8	135.1	524.0	5.9	3.7	14.4
FRMD4A	21.6	111.7	357.6	302.2	5.2	16.5	14.0
SLC1A4	54.9	646.7	181.3	754.8	11.8	3.3	13.8
THBS2	958.8	4107.4	5198.3	12852.0	4.3	5.4	13.4
VEGFA	37.0	741.7	223.3	475.1	20.1	6.0	12.8
DLL1	61.6	261.9	252.5	774.2	4.2	4.1	12.6
FBLN1	28.5	97.2	127.2	342.0	3.4	4.5	12.0
ASS1	1061.8	10886.7	1225.8	12699.7	10.3	1.2	12.0
RFC5	32.1	90.2	314.9	344.2	2.8	9.8	10.7
TGIF2	31.2	63.0	106.0	331.6	2.0	3.4	10.6
HIP1	39.8	109.9	115.0	379.2	2.8	2.9	9.5
PGF	67.4	191.8	125.9	630.6	2.8	1.9	9.4
NUPR1	759.4	6270.0	1375.7	7089.4	8.3	1.8	9.3
ALDH1L2	152.5	960.5	153.3	1407.4	6.3	1.0	9.2
FUT1	39.5	148.4	115.7	363.9	3.8	2.9	9.2
ABCC3	61.1	290.0	149.4	561.2	4.7	2.4	9.2
COL18A1	102.1	550.4	191.2	936.1	5.4	1.9	9.2
FAM167A	63.4	337.1	233.1	575.9	5.3	3.7	9.1
DAPL1	269.9	2084.4	571.2	2386.7	7.7	2.1	8.8
TP53AIP1	101.3	256.0	211.2	891.7	2.5	2.1	8.8

OLFML2A	56.0	344.3	79.9	488.3	6.1	1.4	8.7
SDK2	170.4	835.7	293.0	1477.1	4.9	1.7	8.7
INPP5D	35.2	109.8	69.7	300.8	3.1	2.0	8.6
MOCOS	257.8	2166.4	877.3	2201.2	8.4	3.4	8.5
CDC25B	393.1	2404.8	1342.8	3247.0	6.1	3.4	8.3
IRS1	49.3	125.9	171.8	397.1	2.6	3.5	8.1
KIAA0146	37.8	103.6	73.3	302.2	2.7	1.9	8.0
SYBU	143.1	356.7	328.7	1135.9	2.5	2.3	7.9
IL33	102.8	1128.6	111.5	811.2	11.0	1.1	7.9
THNSL1	55.3	161.4	216.5	432.5	2.9	3.9	7.8
NEFL	70.5	299.4	220.0	544.1	4.2	3.1	7.7
CNOT6L	46.5	148.4	123.9	346.8	3.2	2.7	7.5
STMN3	49.7	102.1	97.8	359.6	2.1	2.0	7.2
FAM107B	97.2	250.0	227.8	669.0	2.6	2.3	6.9
C18orf56	59.2	96.4	122.1	406.3	1.6	2.1	6.9
TRIB3	1307.9	6737.9	2473.0	8963.2	5.2	1.9	6.9
RAD51C	128.6	438.4	631.4	879.3	3.4	4.9	6.8
JDP2	242.4	1511.0	583.5	1613.5	6.2	2.4	6.7
NAA38	50.1	152.4	69.3	331.1	3.0	1.4	6.6
CMBL	245.7	851.9	684.7	1620.4	3.5	2.8	6.6
C9orf91	51.5	135.6	77.2	339.3	2.6	1.5	6.6
MND1	197.8	572.9	714.1	1300.8	2.9	3.6	6.6
ALDH2	447.8	3824.2	641.3	2931.4	8.5	1.4	6.5
RNASEH2B	53.3	44.8	142.3	348.0	-1.2	2.7	6.5
C12orf76	49.8	82.5	108.8	323.6	1.7	2.2	6.5
CDC25C	53.0	222.6	149.1	338.1	4.2	2.8	6.4
ARHGAP19	148.1	447.8	440.7	913.6	3.0	3.0	6.2
CLUAP1	62.1	120.6	156.9	375.6	1.9	2.5	6.0
ETV5	251.8	953.3	717.8	1499.8	3.8	2.9	6.0
PSIP1	163.2	479.6	583.4	970.0	2.9	3.6	5.9
WDR76	107.6	97.1	274.2	637.0	-1.1	2.5	5.9
CPS1	241.5	587.1	464.1	1408.9	2.4	1.9	5.8
TAF5	72.7	171.4	312.4	421.4	2.4	4.3	5.8
TUBE1	429.2	2331.8	777.9	2478.9	5.4	1.8	5.8
NUDT6	99.4	254.0	319.7	560.4	2.6	3.2	5.6
TSC22D3	389.2	1885.2	793.7	2152.5	4.8	2.0	5.5
FBXO31	202.5	433.7	445.8	1114.8	2.1	2.2	5.5
VEZF1	106.2	290.5	444.3	583.0	2.7	4.2	5.5
SKP2	261.4	498.3	861.7	1431.8	1.9	3.3	5.5
PXK	73.3	348.6	73.3	398.9	4.8	1.0	5.4
RBMS3	58.6	124.6	126.0	319.1	2.1	2.1	5.4
EIF2S2	101.8	140.9	270.4	551.7	1.4	2.7	5.4
COL5A2	338.9	819.1	1089.0	1835.4	2.4	3.2	5.4
CALML3	128.1	612.2	304.9	685.6	4.8	2.4	5.4
PDGFA	115.5	210.0	367.3	615.4	1.8	3.2	5.3

NRG1	441.8	1115.3	1468.2	2351.7	2.5	3.3	5.3
PDK3	98.9	261.4	261.0	525.7	2.6	2.6	5.3
ARG2	543.2	1990.2	1000.8	2885.2	3.7	1.8	5.3
CLEC2B	97.0	256.8	212.7	512.6	2.6	2.2	5.3
SCMH1	79.2	128.2	191.9	415.0	1.6	2.4	5.2
DKK3	89.9	246.5	164.5	469.5	2.7	1.8	5.2
GINS4	60.1	121.2	322.0	313.7	2.0	5.4	5.2
FHL1	70.8	228.7	147.1	369.1	3.2	2.1	5.2
DUT	278.6	672.9	940.2	1430.6	2.4	3.4	5.1
ADORA2B	413.9	832.2	1554.9	2093.2	2.0	3.8	5.1
KIF15	61.4	177.3	138.8	309.0	2.9	2.3	5.0
CBS	94.0	201.8	93.7	472.6	2.1	1.0	5.0
LTB4R	112.3	943.3	144.4	564.0	8.4	1.3	5.0
PCK2	1090.7	4824.3	1139.2	5467.3	4.4	1.0	5.0
ZNF496	74.3	193.9	157.3	369.8	2.6	2.1	5.0
SORL1	240.4	1060.6	569.2	1195.6	4.4	2.4	5.0
SLC3A2	1269.4	4952.7	2254.1	6312.0	3.9	1.8	5.0
PATZ1	88.0	202.4	250.5	437.1	2.3	2.8	5.0
MXRA5	295.1	674.7	496.9	1458.2	2.3	1.7	4.9
BCL11A	334.0	1047.8	501.6	1634.4	3.1	1.5	4.9
CHEK1	73.3	113.8	397.9	357.4	1.6	5.4	4.9
KCTD15	925.8	5565.7	1473.3	4467.5	6.0	1.6	4.8
ACTA2	116.2	177.6	304.3	551.4	1.5	2.6	4.7
TSHZ1	75.7	162.0	215.1	356.4	2.1	2.8	4.7
GPX8	449.5	1754.9	1023.2	2091.7	3.9	2.3	4.7
FAM129A	277.3	1056.6	454.0	1268.1	3.8	1.6	4.6
F2R	218.7	285.6	466.0	986.3	1.3	2.1	4.5
RPL37	316.8	685.7	569.6	1417.2	2.2	1.8	4.5
PIGL	151.8	332.2	262.5	678.7	2.2	1.7	4.5
CDC7	207.6	390.2	552.7	924.7	1.9	2.7	4.5
CA12	648.1	2465.0	1132.5	2875.7	3.8	1.7	4.4
IMPA2	1059.1	3601.7	2227.0	4692.1	3.4	2.1	4.4
WDHD1	87.6	133.3	317.6	388.2	1.5	3.6	4.4
ODZ2	3688.2	15256.7	7252.8	16320.2	4.1	2.0	4.4
TXNIP	1858.0	8163.1	3913.4	8187.5	4.4	2.1	4.4
TNS3	99.0	158.0	237.7	435.7	1.6	2.4	4.4
KRTCAP3	274.3	835.4	601.6	1205.2	3.0	2.2	4.4
HS6ST2	104.0	219.8	240.7	456.9	2.1	2.3	4.4
PPARGC1B	141.9	230.4	289.4	618.2	1.6	2.0	4.4
RRM1	109.3	224.3	524.3	476.1	2.1	4.8	4.4
SYNJ2	80.3	93.3	264.3	347.7	1.2	3.3	4.3
CCDC8	305.0	579.8	843.1	1318.5	1.9	2.8	4.3
ANP32E	106.4	92.0	539.7	458.7	-1.2	5.1	4.3
NEIL3	94.3	114.7	223.4	405.1	1.2	2.4	4.3
MTHFD2	5078.2	14566.9	9831.2	21730.3	2.9	1.9	4.3

CHEK2	130.6	228.5	425.1	557.0	1.8	3.3	4.3
NFIB	94.7	126.5	335.1	403.4	1.3	3.5	4.3
CEP120	289.9	790.9	487.2	1220.4	2.7	1.7	4.2
APBB2	139.8	325.5	224.3	586.6	2.3	1.6	4.2
PSPH	1585.7	5290.9	2519.9	6651.0	3.3	1.6	4.2
PPP1R14A	90.6	128.2	244.4	377.3	1.4	2.7	4.2
SHMT2	1132.4	2538.9	2120.5	4699.0	2.2	1.9	4.1
SCARA3	121.1	269.3	213.0	496.1	2.2	1.8	4.1
NSMAF	112.2	95.0	245.2	458.6	-1.2	2.2	4.1
DNMT3B	99.2	398.1	162.1	405.3	4.0	1.6	4.1
DLK2	127.8	128.9	366.0	520.9	1.0	2.9	4.1
C1orf135	82.8	81.2	256.9	334.6	1.0	3.1	4.0
CDCA7	1075.1	1804.6	3327.3	4341.1	1.7	3.1	4.0
GPNMB	221.3	551.3	255.3	888.6	2.5	1.2	4.0
OIP5	265.5	459.5	902.6	1052.2	1.7	3.4	4.0
CDCA7L	1110.3	1697.0	3014.7	4394.9	1.5	2.7	4.0
SIX4	130.2	230.5	321.9	510.4	1.8	2.5	3.9
VLDLR	129.4	490.9	163.6	502.8	3.8	1.3	3.9
LOC642587	468.7	2070.9	834.9	1820.3	4.4	1.8	3.9
BIRC5	125.6	238.3	389.9	487.0	1.9	3.1	3.9
WLS	327.0	580.1	841.7	1265.8	1.8	2.6	3.9
TIMELESS	325.2	375.8	1000.3	1256.7	1.2	3.1	3.9
DLX1	221.3	960.9	420.3	853.6	4.3	1.9	3.9
C16orf5	256.6	355.6	607.8	987.3	1.4	2.4	3.8
SAP30	298.9	364.0	1137.0	1148.9	1.2	3.8	3.8
AKR1C2	1717.2	12556.6	1836.5	6586.1	7.3	1.1	3.8
JAG2	141.5	131.5	325.9	542.0	-1.1	2.3	3.8
LOC100132[illegible]	230.0	493.7	420.1	880.7	2.1	1.8	3.8
CALML4	103.5	422.6	189.8	396.2	4.1	1.8	3.8
ABI2	86.3	100.7	186.3	330.1	1.2	2.2	3.8
NR2C2AP	79.8	81.2	202.0	304.5	1.0	2.5	3.8
IQGAP3	218.3	572.5	381.5	825.2	2.6	1.7	3.8
CRTAP	345.5	635.0	1101.1	1300.3	1.8	3.2	3.8
PRPS2	199.3	273.0	489.1	747.5	1.4	2.5	3.8
PLCD3	125.6	272.4	184.4	469.8	2.2	1.5	3.7
RAD18	99.9	101.3	238.7	371.0	1.0	2.4	3.7
MCM2	621.2	713.1	1653.6	2300.6	1.1	2.7	3.7
NPM3	1086.2	1768.5	2866.6	3993.3	1.6	2.6	3.7
POLQ	153.0	270.5	554.2	562.6	1.8	3.6	3.7
PHGDH	917.6	2382.5	1013.6	3372.6	2.6	1.1	3.7
AKR1C1	1488.3	11283.6	1689.0	5460.8	7.6	1.1	3.7
ASNS	7255.9	19773.9	5732.4	26300.3	2.7	-1.3	3.6
DYNC2H1	231.3	391.5	546.1	825.6	1.7	2.4	3.6
CELF2	166.0	129.6	354.4	592.0	-1.3	2.1	3.6
FAM40B	126.2	471.6	217.4	446.9	3.7	1.7	3.5

PRKCA	121.7	373.3	186.1	430.8	3.1	1.5	3.5
H2AFV	131.9	123.4	345.1	466.4	-1.1	2.6	3.5
BAG2	80.0	30.5	338.7	281.8	-2.6	4.2	3.5
FAM92A1	177.7	237.9	548.9	623.6	1.3	3.1	3.5
C11orf82	116.8	72.5	508.3	409.2	-1.6	4.4	3.5
C1orf77	208.0	198.9	973.9	728.4	1.0	4.7	3.5
C14orf167	195.3	482.1	324.3	677.9	2.5	1.7	3.5
POLE2	293.3	413.4	1059.3	1014.7	1.4	3.6	3.5
ABHD10	114.8	190.2	363.6	396.4	1.7	3.2	3.5
TEF	256.0	553.1	283.4	880.9	2.2	1.1	3.4
ME2	169.8	173.3	450.7	583.0	1.0	2.7	3.4
SLC7A5	9285.7	25772.8	14448.4	31761.3	2.8	1.6	3.4
GRK6	97.8	163.2	227.4	333.4	1.7	2.3	3.4
TSPAN4	245.5	340.8	1013.3	836.2	1.4	4.1	3.4
RAB7B	205.9	330.6	458.0	698.0	1.6	2.2	3.4
HIRIP3	122.4	152.3	353.8	414.4	1.2	2.9	3.4
C14orf159	185.8	499.9	309.6	623.4	2.7	1.7	3.4
MDC1	181.2	273.6	511.9	606.7	1.5	2.8	3.3
MAD2L1	189.8	293.1	1384.7	634.2	1.5	7.3	3.3
SPARC	489.9	540.2	1464.6	1602.8	1.1	3.0	3.3
CCDC113	618.2	1341.4	929.4	2018.3	2.2	1.5	3.3
ADSSL1	84.4	372.9	89.9	274.0	4.4	1.1	3.2
NEK6	275.8	439.0	641.8	889.7	1.6	2.3	3.2
C21orf91	1768.1	4000.4	2475.3	5687.3	2.3	1.4	3.2
C19orf54	280.9	636.6	402.5	902.8	2.3	1.4	3.2
RHBDD1	128.8	329.7	205.2	413.2	2.6	1.6	3.2
SLC38A5	87.9	105.9	316.2	281.4	1.2	3.6	3.2
SUV39H2	247.7	270.8	591.5	783.2	1.1	2.4	3.2
TM7SF3	395.3	589.1	892.8	1248.8	1.5	2.3	3.2
PTK7	656.3	1429.4	949.4	2068.5	2.2	1.4	3.2
HADH	296.3	451.9	704.3	930.6	1.5	2.4	3.1
C9orf140	236.4	325.0	483.9	737.1	1.4	2.0	3.1
HSPA13	1151.0	3220.4	1592.4	3581.6	2.8	1.4	3.1
CNTN1	368.4	839.6	416.1	1145.4	2.3	1.1	3.1
DTL	196.1	98.7	504.4	608.0	-2.0	2.6	3.1
JUB	717.0	1646.4	1041.5	2204.6	2.3	1.5	3.1
EXO1	130.1	172.3	392.9	395.9	1.3	3.0	3.0
GINS2	841.9	974.4	2435.3	2545.5	1.2	2.9	3.0
RFC3	96.1	54.8	365.4	289.9	-1.8	3.8	3.0
KBTBD6	135.8	89.1	311.6	408.6	-1.5	2.3	3.0
HAUS6	106.1	53.6	244.1	318.0	-2.0	2.3	3.0
NLRP1	107.1	214.5	132.2	320.3	2.0	1.2	3.0
C4orf46	162.9	182.8	365.2	487.0	1.1	2.2	3.0
COX6A1	454.8	368.8	6431.8	1297.8	-1.2	14.1	2.9
UNC5B	758.4	2296.4	465.5	2114.5	3.0	-1.6	2.8

NANP	202.4	144.5	614.0	553.7	-1.4	3.0	2.7
AP1S1	371.4	453.1	1344.7	1007.9	1.2	3.6	2.7
CDC45	259.3	287.8	789.7	698.0	1.1	3.0	2.7
NRP1	105.6	111.2	377.0	279.3	1.1	3.6	2.6
MAF	214.2	1220.4	145.9	558.5	5.7	-1.5	2.6
ARL4C	1401.0	1392.1	4535.5	3579.7	1.0	3.2	2.6
FH	221.9	214.8	853.0	558.0	1.0	3.8	2.5
DLX2	265.7	1303.7	252.1	652.3	4.9	-1.1	2.5
MCM10	304.5	243.4	1010.8	744.9	-1.3	3.3	2.4
MTPAP	122.7	95.7	392.8	291.8	-1.3	3.2	2.4
PNKD	5.1	4.7	355.9	11.6	-1.1	70.3	2.3
C10orf99	372.2	2589.6	373.1	840.5	7.0	1.0	2.3
LOC203274	169.1	582.9	140.0	343.0	3.4	-1.2	2.0
ME1	12.5	8.3	587.5	23.6	-1.5	47.1	1.9
CDV3	110.8	84.8	362.1	205.2	-1.3	3.3	1.9
RAC2	49.6	37.3	347.5	89.7	-1.3	7.0	1.8
PHLDA1	96.2	81.5	322.0	169.4	-1.2	3.3	1.8
PPIF	67.8	42.6	320.9	101.3	-1.6	4.7	1.5
GSTA4	154.0	687.7	97.8	213.9	4.5	-1.6	1.4
RDH12	556.4	1737.4	328.4	738.8	3.1	-1.7	1.3
PRR9	732.1	3863.6	300.7	970.3	5.3	-2.4	1.3
C6orf141	155.4	516.4	63.1	193.4	3.3	-2.5	1.2
BPIL2	200.2	690.6	83.4	200.2	3.5	-2.4	1.0
LOC1001315(	396.1	1167.5	114.4	335.1	2.9	-3.5	-1.2
KIAA0754	60.9	335.2	7.7	49.6	5.5	-7.9	-1.2
CBFA2T2	341.1	620.4	110.3	255.7	1.8	-3.1	-1.3
FAM111B	190.7	48.8	561.7	142.5	-3.9	2.9	-1.3
CDC73	305.5	52.6	521.7	207.9	-5.8	1.7	-1.5
MGEA5	636.5	915.1	135.3	424.0	1.4	-4.7	-1.5
WDR11	231.0	364.0	69.0	153.6	1.6	-3.4	-1.5
IL23A	253.5	879.0	359.9	166.7	3.5	1.4	-1.5
PRKAG2	319.8	102.7	390.4	194.2	-3.1	1.2	-1.6
DSG2	1528.4	462.4	1894.1	924.5	-3.3	1.2	-1.7
NCRNA0018	1076.1	2619.4	312.8	650.5	2.4	-3.4	-1.7
CSNK1A1	944.2	1212.6	279.3	559.9	1.3	-3.4	-1.7
SAMD4A	518.1	157.6	760.0	299.3	-3.3	1.5	-1.7
CTGF	1733.3	291.7	2413.8	916.2	-5.9	1.4	-1.9
PALM2-AKAP	1477.3	406.6	1805.7	764.2	-3.6	1.2	-1.9
RUFY3	1563.0	2402.4	493.2	752.1	1.5	-3.2	-2.1
LOC653602	992.9	1519.0	291.7	446.3	1.5	-3.4	-2.2
JMJD1C	326.2	364.5	71.6	146.2	1.1	-4.6	-2.2
AFF4	354.1	415.5	69.3	154.4	1.2	-5.1	-2.3
ZNF207	581.5	510.5	169.7	252.4	-1.1	-3.4	-2.3
LOC100288	3163.1	9502.0	1288.1	1337.0	3.0	-2.5	-2.4
UBASH3B	545.2	183.8	490.5	227.9	-3.0	-1.1	-2.4

C15orf29	773.6	947.9	207.6	308.7	1.2	-3.7	-2.5
TGFBR2	9516.6	2067.0	9656.7	3773.2	-4.6	1.0	-2.5
FLJ40330	184.3	408.0	28.9	71.7	2.2	-6.4	-2.6
NAA25	304.4	252.4	22.6	118.0	-1.2	-13.5	-2.6
PGM2L1	419.7	60.2	359.9	157.7	-7.0	-1.2	-2.7
NAMPT	291.7	426.6	71.5	108.7	1.5	-4.1	-2.7
GJA5	198.5	34.6	305.2	70.8	-5.7	1.5	-2.8
SVIL	454.7	146.2	367.3	161.9	-3.1	-1.2	-2.8
AP1S3	910.6	191.4	973.0	322.1	-4.8	1.1	-2.8
ZDHHC21	207.4	382.4	43.9	73.2	1.8	-4.7	-2.8
TSPAN1	2103.0	367.0	2873.0	735.6	-5.7	1.4	-2.9
CYP3A5	295.7	1056.9	97.7	103.0	3.6	-3.0	-2.9
STAT1	193.9	33.2	345.2	67.3	-5.8	1.8	-2.9
PELI1	320.0	678.7	21.4	109.2	2.1	-15.0	-2.9
WFDC5	1016.1	1093.8	344.0	343.9	1.1	-3.0	-3.0
MAST4	479.9	601.5	108.5	162.3	1.3	-4.4	-3.0
TDRD7	1704.1	338.7	1206.7	574.8	-5.0	-1.4	-3.0
PCYT1A	397.7	158.2	299.9	134.0	-2.5	-1.3	-3.0
DKK1	9753.5	2886.3	12759.1	3227.4	-3.4	1.3	-3.0
TCHH	397.7	398.2	116.3	127.3	1.0	-3.4	-3.1
INTU	466.5	395.0	136.6	148.8	-1.2	-3.4	-3.1
CPEB2	7408.2	5940.7	3046.4	2362.2	-1.2	-2.4	-3.1
PCSK5	656.5	43.3	269.9	209.2	-15.2	-2.4	-3.1
TMEM63A	667.6	431.8	213.8	212.2	-1.5	-3.1	-3.1
PCMTD1	1357.4	1290.4	505.9	422.0	1.0	-2.7	-3.2
FAM114A1	1780.6	663.3	1126.4	550.1	-2.7	-1.6	-3.2
IL1F5	2403.9	1867.9	937.7	737.9	-1.3	-2.6	-3.3
CLCA4	2330.7	537.3	1777.6	709.1	-4.3	-1.3	-3.3
APOBEC3B	477.2	75.6	310.6	144.7	-6.3	-1.5	-3.3
CEP350	370.3	285.0	114.4	111.9	-1.3	-3.2	-3.3
HERC6	1768.6	385.2	1158.4	532.4	-4.6	-1.5	-3.3
DLG1	1259.1	1262.3	291.9	378.7	1.0	-4.3	-3.3
PYGB	7566.9	1677.0	4562.4	2270.5	-4.5	-1.7	-3.3
CDKN2B	4485.5	3119.5	1469.3	1339.6	-1.4	-3.1	-3.3
CWH43	8017.1	5253.1	3642.4	2379.0	-1.5	-2.2	-3.4
CLIP1	663.7	422.7	201.1	195.0	-1.6	-3.3	-3.4
DNER	519.0	46.7	371.8	152.4	-11.1	-1.4	-3.4
SHF	733.3	483.6	260.1	212.2	-1.5	-2.8	-3.5
CDA	2708.7	1079.6	1829.5	783.5	-2.5	-1.5	-3.5
EHF	874.5	1169.9	235.3	248.8	1.3	-3.7	-3.5
TYMP	515.4	431.8	214.6	146.4	-1.2	-2.4	-3.5
FLG	4872.3	2877.4	1811.9	1378.4	-1.7	-2.7	-3.5
ABAT	3163.6	765.9	1960.9	892.0	-4.1	-1.6	-3.5
VGLL1	3137.5	741.7	2119.3	880.3	-4.2	-1.5	-3.6
DUOX1	4653.9	3747.8	1718.2	1303.8	-1.2	-2.7	-3.6

IFI6	17312.3	7351.6	13033.9	4848.2	-2.4	-1.3	-3.6
TFRC	5870.7	935.6	10640.8	1615.4	-6.3	1.8	-3.6
THBS1	1180.8	296.6	1206.7	323.4	-4.0	1.0	-3.7
ELOVL4	3079.6	1732.7	1237.2	842.7	-1.8	-2.5	-3.7
IFI44L	1993.8	775.2	1119.7	542.9	-2.6	-1.8	-3.7
KLF6	1996.9	618.9	1771.0	541.3	-3.2	-1.1	-3.7
ARHGAP29	12798.4	4003.6	10138.2	3447.6	-3.2	-1.3	-3.7
ZDHHC11	552.4	381.5	148.5	148.5	-1.4	-3.7	-3.7
DDX58	395.1	32.8	404.9	106.2	-12.1	1.0	-3.7
SERPINB2	34895.1	12429.9	23468.3	9286.9	-2.8	-1.5	-3.8
ACSL1	2398.8	1091.0	1303.3	627.8	-2.2	-1.8	-3.8
S100A4	7832.0	2486.1	5138.4	2045.7	-3.2	-1.5	-3.8
TMTC2	479.0	238.5	267.7	124.7	-2.0	-1.8	-3.8
DSC2	11671.4	8607.2	5147.3	3022.1	-1.4	-2.3	-3.9
VSIG10L	499.5	554.5	160.3	128.4	1.1	-3.1	-3.9
RANBP2	977.1	595.3	344.4	250.4	-1.6	-2.8	-3.9
FXYD3	7282.7	3799.9	2634.9	1863.3	-1.9	-2.8	-3.9
TMEM184A	518.2	851.6	191.1	132.4	1.6	-2.7	-3.9
CYP51A1	4600.0	1980.8	2556.4	1166.6	-2.3	-1.8	-3.9
PVRL4	2131.3	1267.3	739.1	539.4	-1.7	-2.9	-4.0
GABBR1	277.1	410.3	123.4	69.8	1.5	-2.2	-4.0
SULT2B1	3548.4	2210.9	1447.3	885.9	-1.6	-2.5	-4.0
GPR109B	1942.8	523.6	1254.8	482.9	-3.7	-1.5	-4.0
RBP7	549.7	294.4	206.0	136.6	-1.9	-2.7	-4.0
EME2	2040.6	1343.5	862.4	507.0	-1.5	-2.4	-4.0
MACC1	471.2	68.5	256.2	116.8	-6.9	-1.8	-4.0
HYAL1	1932.7	1181.8	794.4	479.1	-1.6	-2.4	-4.0
IDS	1027.3	249.9	613.9	254.1	-4.1	-1.7	-4.0
TET2	632.5	367.5	190.5	155.6	-1.7	-3.3	-4.1
PNPLA3	511.5	602.7	231.1	125.7	1.2	-2.2	-4.1
OAS1	2556.8	594.1	1786.9	627.8	-4.3	-1.4	-4.1
NEAT1	3938.1	15486.3	831.0	958.0	3.9	-4.7	-4.1
ATP13A2	770.8	279.9	376.4	185.9	-2.8	-2.0	-4.1
PKIB	544.6	194.7	503.8	131.2	-2.8	-1.1	-4.2
CCND1	5133.5	1281.0	2701.5	1233.7	-4.0	-1.9	-4.2
SP110	1026.9	236.5	770.7	246.1	-4.3	-1.3	-4.2
DDX17	825.4	1104.6	199.0	196.4	1.3	-4.1	-4.2
STEAP4	882.1	458.9	313.8	209.3	-1.9	-2.8	-4.2
UPK1B	5381.3	1388.3	3320.6	1267.1	-3.9	-1.6	-4.2
MEG3	112.2	324.2	45.6	26.3	2.9	-2.5	-4.3
B4GALNT3	1065.0	740.8	385.4	249.5	-1.4	-2.8	-4.3
FLG2	1197.1	900.1	507.3	278.9	-1.3	-2.4	-4.3
IL1F9	412.0	445.6	132.5	96.0	1.1	-3.1	-4.3
CLIC3	12689.8	4505.0	5981.5	2940.8	-2.8	-2.1	-4.3
RHCG	7028.3	2529.3	3343.8	1619.5	-2.8	-2.1	-4.3

SQLE	2963.8	1162.8	1769.7	673.3	-2.5	-1.7	-4.4
PTPRO	9963.8	17219.9	2310.8	2229.3	1.7	-4.3	-4.5
KIFC2	219.1	303.1	51.1	49.0	1.4	-4.3	-4.5
CLDN1	3562.6	1783.6	1654.0	795.8	-2.0	-2.2	-4.5
TP53I3	6723.3	2951.1	4114.5	1495.4	-2.3	-1.6	-4.5
DAPP1	2813.7	838.3	1255.3	616.4	-3.4	-2.2	-4.6
CYB5R2	6105.7	1925.0	2764.8	1337.2	-3.2	-2.2	-4.6
C10orf116	16622.4	4671.2	8482.6	3629.6	-3.6	-2.0	-4.6
MALAT1	3234.2	3007.6	861.7	706.0	-1.1	-3.8	-4.6
RASEF	4578.8	5007.0	1438.5	995.2	1.1	-3.2	-4.6
ANGPTL4	628.3	86.7	477.3	136.1	-7.2	-1.3	-4.6
USP18	551.3	21.0	338.4	118.0	-26.3	-1.6	-4.7
PARP9	2120.2	482.6	1359.1	451.6	-4.4	-1.6	-4.7
ROR1	436.0	16.3	328.2	92.6	-26.8	-1.3	-4.7
ISG15	21826.5	5526.4	12355.0	4584.7	-3.9	-1.8	-4.8
CD24	22777.1	8621.2	13361.7	4701.2	-2.6	-1.7	-4.8
RASA1	7016.0	1689.8	3037.6	1435.7	-4.2	-2.3	-4.9
MFI2	1102.3	177.3	544.4	225.4	-6.2	-2.0	-4.9
DCBLD2	3104.8	973.1	2617.7	633.3	-3.2	-1.2	-4.9
IVL	47673.6	17240.1	22937.5	9694.1	-2.8	-2.1	-4.9
RAPGEF2	339.9	154.9	98.4	68.0	-2.2	-3.5	-5.0
TGFA	358.4	78.8	161.2	70.9	-4.5	-2.2	-5.1
STS	498.4	75.7	198.5	97.3	-6.6	-2.5	-5.1
SEC14L2	349.4	63.1	199.2	68.0	-5.5	-1.8	-5.1
IGFL2	11833.8	3529.1	8172.0	2291.1	-3.4	-1.4	-5.2
EPB41L4B	880.1	151.1	377.5	169.0	-5.8	-2.3	-5.2
PEA15	810.5	194.0	454.7	155.3	-4.2	-1.8	-5.2
HSPC159	3893.1	2271.4	1198.4	745.5	-1.7	-3.2	-5.2
ATL1	860.0	178.8	431.0	164.3	-4.8	-2.0	-5.2
IRF7	1119.4	361.0	600.2	213.1	-3.1	-1.9	-5.3
ECM1	2687.0	731.3	1722.8	509.8	-3.7	-1.6	-5.3
CTSL2	31051.7	7405.2	16220.5	5868.8	-4.2	-1.9	-5.3
CBX6	819.7	148.7	375.1	154.6	-5.5	-2.2	-5.3
LPHN2	5097.8	2011.3	1840.3	956.2	-2.5	-2.8	-5.3
PLD1	923.8	505.9	123.1	172.8	-1.8	-7.5	-5.3
ALDH1A1	1133.1	162.5	484.5	211.3	-7.0	-2.3	-5.4
TRAF3IP3	3386.7	978.8	1349.9	627.9	-3.5	-2.5	-5.4
S100P	39361.8	17227.7	19040.4	7215.6	-2.3	-2.1	-5.5
DUOXA1	3560.9	2546.1	1266.4	652.0	-1.4	-2.8	-5.5
PLBD1	8336.9	2558.3	3365.0	1520.8	-3.3	-2.5	-5.5
C10orf118	319.0	133.8	77.1	57.3	-2.4	-4.1	-5.6
KLK11	7714.7	3481.1	3875.7	1386.7	-2.2	-2.0	-5.6
KRT13	86958.1	20586.2	49415.4	15615.4	-4.2	-1.8	-5.6
RGS2	20383.6	5273.0	11156.7	3656.2	-3.9	-1.8	-5.6
KLK10	31489.9	8707.5	17585.1	5647.4	-3.6	-1.8	-5.6

SLC44A1	325.8	117.6	115.1	58.4	-2.8	-2.8	-5.6
RRAD	315.3	46.6	268.8	56.4	-6.8	-1.2	-5.6
IL1RN	3505.0	741.2	3281.7	627.3	-4.7	-1.1	-5.6
SDCBP2	3233.5	735.9	1299.6	575.5	-4.4	-2.5	-5.6
CD55	8722.3	3660.9	3268.4	1545.7	-2.4	-2.7	-5.6
SYTL2	1010.0	215.8	405.1	178.8	-4.7	-2.5	-5.6
TM4SF1	14023.4	1895.5	5780.6	2463.7	-7.4	-2.4	-5.7
IFIT1	10135.5	1520.4	5577.9	1779.6	-6.7	-1.8	-5.7
C1orf104	2732.9	1778.7	774.0	477.2	-1.5	-3.5	-5.7
A2ML1	30212.1	6519.4	14636.4	5230.9	-4.6	-2.1	-5.8
C1orf74	4813.2	1230.6	2140.9	832.3	-3.9	-2.2	-5.8
CPEB4	2165.9	646.2	805.7	365.1	-3.4	-2.7	-5.9
AKAP13	1188.1	221.7	412.1	198.7	-5.4	-2.9	-6.0
TAF13	608.6	337.2	236.9	101.6	-1.8	-2.6	-6.0
KLK5	17365.4	3807.2	11733.7	2895.7	-4.6	-1.5	-6.0
XAF1	823.3	157.5	354.2	135.9	-5.2	-2.3	-6.1
SOX9	9426.6	1890.7	3836.0	1544.3	-5.0	-2.5	-6.1
IFI27	6650.3	1775.9	4862.1	1080.1	-3.7	-1.4	-6.2
SYTL4	686.0	118.0	353.2	111.3	-5.8	-1.9	-6.2
HES4	1545.4	214.1	679.5	247.7	-7.2	-2.3	-6.2
CNFN	2403.3	1135.9	822.2	384.0	-2.1	-2.9	-6.3
PRICKLE1	415.9	36.6	160.3	66.2	-11.4	-2.6	-6.3
CCDC80	622.2	109.2	287.4	98.9	-5.7	-2.2	-6.3
CD59	1355.5	229.7	629.4	213.8	-5.9	-2.2	-6.3
GDPD3	469.0	215.4	169.8	73.7	-2.2	-2.8	-6.4
EDN1	4705.3	543.8	2899.0	737.7	-8.7	-1.6	-6.4
SPRR3	30139.5	8286.2	17036.9	4629.4	-3.6	-1.8	-6.5
SLC2A6	840.7	317.8	408.1	126.9	-2.6	-2.1	-6.6
REEP3	384.8	47.0	189.4	56.5	-8.2	-2.0	-6.8
TUBA1A	11636.6	1865.2	7502.8	1683.4	-6.2	-1.6	-6.9
AQP9	348.1	102.3	160.9	50.2	-3.4	-2.2	-6.9
C14orf34	7244.1	2828.2	2560.6	1038.4	-2.6	-2.8	-7.0
MARCKSL1	6730.4	1509.9	2699.4	944.3	-4.5	-2.5	-7.1
PPP1R3B	1959.5	221.7	762.1	274.3	-8.8	-2.6	-7.1
HMGCS1	1820.9	650.3	1035.9	253.4	-2.8	-1.8	-7.2
CMPK2	3166.7	442.0	1072.8	438.8	-7.2	-3.0	-7.2
MX1	9057.9	2227.8	5390.7	1232.7	-4.1	-1.7	-7.3
CCDC64B	551.3	393.0	194.7	74.7	-1.4	-2.8	-7.4
EPS8L1	2373.9	1227.5	613.5	316.3	-1.9	-3.9	-7.5
PLEKHA7	703.8	231.2	259.5	93.1	-3.0	-2.7	-7.6
KRT4	44519.5	16179.9	24244.2	5878.5	-2.8	-1.8	-7.6
CARD18	3998.9	749.0	2078.0	523.2	-5.3	-1.9	-7.6
C5orf46	1216.9	207.0	368.1	156.0	-5.9	-3.3	-7.8
KRT7	5154.6	953.5	3183.2	660.9	-5.4	-1.6	-7.8
DAPK1	1916.0	380.3	690.7	245.4	-5.0	-2.8	-7.8

LRAT	483.8	60.3	196.0	61.9	-8.0	-2.5	-7.8
LOC1001336	794.3	287.8	151.7	101.4	-2.8	-5.2	-7.8
HOPX	286.6	538.4	40.6	36.1	1.9	-7.1	-7.9
CFLAR	619.7	574.1	59.6	77.9	-1.1	-10.4	-8.0
SLC6A14	17300.2	5673.5	7293.6	2162.4	-3.0	-2.4	-8.0
NID2	1810.7	476.0	570.7	220.7	-3.8	-3.2	-8.2
ISG20	609.2	22.9	210.5	73.9	-26.6	-2.9	-8.2
ANK3	235.4	518.1	14.0	28.3	2.2	-16.8	-8.3
CERCAM	355.1	83.0	202.2	42.2	-4.3	-1.8	-8.4
CLDN8	395.6	68.4	124.2	46.7	-5.8	-3.2	-8.5
EGR3	1725.6	847.9	526.3	199.7	-2.0	-3.3	-8.6
CXCL6	970.2	88.1	446.0	111.6	-11.0	-2.2	-8.7
HSPB8	9484.0	2299.3	2990.8	1075.7	-4.1	-3.2	-8.8
HPGD	437.0	58.4	136.9	48.5	-7.5	-3.2	-9.0
CDKN1C	546.3	222.6	119.7	60.5	-2.5	-4.6	-9.0
C15orf52	1658.1	687.1	636.8	183.2	-2.4	-2.6	-9.1
CGN	456.6	91.6	159.5	49.7	-5.0	-2.9	-9.2
ZNF365	1019.9	250.3	527.9	110.7	-4.1	-1.9	-9.2
POF1B	833.2	400.0	163.7	88.9	-2.1	-5.1	-9.4
ID2	9338.9	4863.4	1767.8	992.1	-1.9	-5.3	-9.4
KLK7	55449.7	13708.6	28952.4	5862.9	-4.0	-1.9	-9.5
RAB11FIP1	14108.6	1828.0	4678.5	1467.4	-7.7	-3.0	-9.6
SCEL	3479.5	994.5	1434.1	360.3	-3.5	-2.4	-9.7
NDRG2	2057.0	385.2	779.9	212.8	-5.3	-2.6	-9.7
LOR	698.4	58.4	300.1	72.2	-12.0	-2.3	-9.7
ALDH1A3	37260.5	8163.2	14854.6	3834.7	-4.6	-2.5	-9.7
CLDN4	4570.4	493.6	1492.7	469.2	-9.3	-3.1	-9.7
EMP1	3876.8	242.0	856.5	394.4	-16.0	-4.5	-9.8
PLA2G7	1064.4	186.2	420.5	106.8	-5.7	-2.5	-10.0
PLD5	1729.9	224.2	1179.6	172.7	-7.7	-1.5	-10.0
KLK12	312.6	60.6	89.4	29.5	-5.2	-3.5	-10.6
CAMK2N1	5951.0	657.1	2166.0	561.3	-9.1	-2.7	-10.6
KRT77	2266.5	984.7	660.5	213.6	-2.3	-3.4	-10.6
WFDC12	819.1	110.1	356.2	76.1	-7.4	-2.3	-10.8
KRT78	500.8	159.1	90.8	45.7	-3.1	-5.5	-11.0
MYLK	3185.4	191.8	920.3	289.8	-16.6	-3.5	-11.0
SLC39A8	563.2	35.4	374.1	49.8	-15.9	-1.5	-11.3
MYO5B	872.1	249.0	229.4	75.3	-3.5	-3.8	-11.6
ENC1	1453.1	124.2	525.4	124.2	-11.7	-2.8	-11.7
EGF	519.5	167.5	113.3	43.4	-3.1	-4.6	-12.0
RSAD2	1191.4	34.7	292.2	99.3	-34.4	-4.1	-12.0
GALNT5	1116.1	216.2	135.8	92.3	-5.2	-8.2	-12.1
ADAMTS1	580.6	77.5	316.4	47.5	-7.5	-1.8	-12.2
KRT23	2206.7	452.2	740.6	180.2	-4.9	-3.0	-12.2
HS3ST1	8556.6	787.4	1866.5	691.6	-10.9	-4.6	-12.4

TNFAIP3	1068.4	223.7	412.5	85.2	-4.8	-2.6	-12.5
LCN2	13610.2	3173.3	6277.9	1078.1	-4.3	-2.2	-12.6
VILL	347.2	62.8	80.8	27.5	-5.5	-4.3	-12.6
IFI44	539.5	290.6	101.4	42.5	-1.9	-5.3	-12.7
CXCL1	1966.0	148.8	1226.9	149.5	-13.2	-1.6	-13.2
VGLL3	7448.1	1067.4	2410.7	553.8	-7.0	-3.1	-13.4
CAPN14	530.9	49.0	222.7	38.9	-10.8	-2.4	-13.7
ABLIM3	476.5	93.0	141.4	34.8	-5.1	-3.4	-13.7
QPRT	2231.9	210.9	689.4	161.4	-10.6	-3.2	-13.8
MAP2	2684.3	660.7	678.4	194.0	-4.1	-4.0	-13.8
KRT19	18679.3	978.5	8672.6	1343.7	-19.1	-2.2	-13.9
LIPH	936.8	99.4	185.6	67.3	-9.4	-5.0	-13.9
ASPRV1	1907.1	250.6	733.6	137.0	-7.6	-2.6	-13.9
KRT80	3822.7	728.3	1115.7	268.5	-5.2	-3.4	-14.2
LOC643008	322.9	92.4	32.2	22.3	-3.5	-10.0	-14.5
MMP9	1647.8	311.1	991.3	113.2	-5.3	-1.7	-14.6
LMO7	5635.9	1062.5	1322.8	385.4	-5.3	-4.3	-14.6
GCNT2	1173.4	33.5	269.0	79.6	-35.0	-4.4	-14.7
SERPINB13	382.1	130.0	41.2	25.9	-2.9	-9.3	-14.8
KLK6	461.7	33.7	183.1	30.8	-13.7	-2.5	-15.0
TMPRSS11E	1524.8	178.7	612.7	98.8	-8.5	-2.5	-15.4
PSG4	804.9	230.9	306.0	48.8	-3.5	-2.6	-16.5
SLC7A2	497.1	30.4	142.1	29.5	-16.4	-3.5	-16.8
SAMD9	14841.9	1981.6	3651.2	867.5	-7.5	-4.1	-17.1
SPRR2G	3852.6	302.7	1166.6	201.9	-12.7	-3.3	-19.1
STXBP6	360.2	6.9	105.3	18.7	-52.1	-3.4	-19.3
TIMP2	945.8	42.5	301.4	49.0	-22.3	-3.1	-19.3
AGPAT9	1585.5	82.9	328.0	78.7	-19.1	-4.8	-20.2
ANXA9	2120.8	358.1	441.2	105.1	-5.9	-4.8	-20.2
NEBL	807.6	522.9	89.1	39.9	-1.5	-9.1	-20.2
SHANK2	461.0	34.6	82.0	22.7	-13.3	-5.6	-20.3
MAP3K8	405.1	80.1	18.7	19.4	-5.1	-21.7	-20.9
C2orf54	584.0	37.4	59.4	27.3	-15.6	-9.8	-21.4
LCE3D	4306.0	689.3	978.8	180.8	-6.2	-4.4	-23.8
CEACAM6	8097.2	964.5	2227.5	333.2	-8.4	-3.6	-24.3
MAL	2273.8	459.5	707.3	90.8	-4.9	-3.2	-25.0
FN1	4504.3	368.2	2538.9	177.8	-12.2	-1.8	-25.3
IL1R2	1549.8	90.8	549.8	57.7	-17.1	-2.8	-26.8
CRCT1	3429.9	498.6	935.3	122.0	-6.9	-3.7	-28.1
CDSN	323.8	21.2	89.4	11.2	-15.2	-3.6	-28.8
GPRC5A	12965.7	730.0	3440.2	447.2	-17.8	-3.8	-29.0
CST6	7132.2	676.6	2374.3	235.5	-10.5	-3.0	-30.3
IL8	2817.6	189.3	532.6	90.9	-14.9	-5.3	-31.0
COX7A1	520.7	80.7	92.0	16.7	-6.5	-5.7	-31.1
CEACAM1	647.9	10.5	202.0	20.2	-61.4	-3.2	-32.1

KLK13	3063.6	94.6	672.1	95.2	-32.4	-4.6	-32.2
ELF3	1827.7	270.9	279.9	55.9	-6.7	-6.5	-32.7
KRT31	584.7	21.0	114.0	16.0	-27.9	-5.1	-36.6
MUC1	785.0	24.7	380.7	20.7	-31.8	-2.1	-37.9
INHBA	886.0	91.9	257.8	21.2	-9.6	-3.4	-41.8
ID4	1849.5	372.5	208.8	39.9	-5.0	-8.9	-46.3
MUCL1	1888.7	74.1	407.1	40.5	-25.5	-4.6	-46.6
LOC1001311	3635.1	100.3	914.2	77.0	-36.2	-4.0	-47.2
PHACTR3	895.4	10.6	509.4	18.9	-84.5	-1.8	-47.5
PRSS27	373.5	27.0	55.7	7.5	-13.8	-6.7	-49.6
SAMD9L	368.9	9.1	48.7	7.4	-40.6	-7.6	-49.9
NLRP10	551.2	35.8	46.8	10.4	-15.4	-11.8	-53.2
GUCY1A3	404.6	6.2	67.8	6.2	-65.0	-6.0	-65.0
DSG4	1115.4	22.0	101.9	13.7	-50.7	-10.9	-81.3
MYCN	788.2	8.7	123.6	8.7	-90.8	-6.4	-90.8
MMP10	1039.2	12.1	84.8	10.3	-85.8	-12.3	-100.6
IGFBP3	816.0	14.5	88.9	7.4	-56.4	-9.2	-111.0
BMP6	731.6	9.9	132.2	6.0	-74.1	-5.5	-122.2
C15orf48	9597.2	181.2	1837.4	62.5	-53.0	-5.2	-153.7
APOBEC3A	2143.0	21.8	71.9	12.9	-98.5	-29.8	-166.0
RARRES1	1105.6	6.4	314.1	6.4	-172.7	-3.5	-173.3
LOC730755	1402.0	16.5	91.5	4.5	-84.9	-15.3	-312.4
GABRP	9834.8	50.9	1211.3	11.1	-193.2	-8.1	-885.9

Table D. Reverse-Phase Protein Array (RPPA) analysis. HFKs were grown in F-medium (F) in the presence of Y-27632 (Y), J2 cells (J2) or J2 cells + Y-27632 (J2+Y). Shown are the normalized signal intensities for the phosphoproteins, which resulted in a change of ³50% vs. F with a standard deviation of ±15%.

	Normalized Raw Score				Ratio		
**Sample ID**	**F**	**Y**	**J2**	**J2+Y**	**Y vs. F**	**J2 vs. F**	**J2+Y vs. F**
p38 MAPK T180/Y182	22452	18337	78901	84553	0.82	3.51	3.77
Ki67	6741	9771	20423	19318	1.45	3.03	2.87
p90RSK S380	3317	5966	13867	8971	1.80	4.18	2.70
AKT T308	3893	4516	3961	9590	1.16	1.02	2.46
Cyclin B1	6069	10333	34168	14241	1.70	5.63	2.35
Heregulin	4459	10435	7325	9848	2.34	1.64	2.21
Src Family Y416	17142	12890	72138	36221	0.75	4.21	2.11
Paxillin Y118	5147	12021	5666	10235	2.34	1.10	1.99
FRS2alpha Y436	3655	2662	6056	6974	0.73	1.66	1.91
Notch1	33420	42264	43301	62289	1.26	1.30	1.86
PKCa S657	1206	1711	2712	2197	1.42	2.25	1.82
GSK3aB S21_9	5496	5292	22637	8710	0.96	4.12	1.58
4EBP1 S65	2782	4284	6681	4188	1.54	2.40	1.51
CDK2	8586	10355	17172	11962	1.21	2.00	1.39
EGFR Y1173	15666	18433	23360	21541	1.18	1.49	1.38
MARCKS S152/156	1351	3195	77613	1789	2.36	57.45	1.32
Acetyl CoA Carboxylase S79	19781	15151	39756	25005	0.77	2.01	1.26
cMyc	7628	7447	14683	9628	0.98	1.92	1.26
eNOS/NOS3 S116	7182	7867	15680	8894	1.10	2.18	1.24
Ron Y1353	10275	18343	14147	12436	1.79	1.38	1.21
Ras GRF1 S916	10234	11865	20081	12312	1.16	1.96	1.20
AMPKbeta 1 S108	2904	4071	12056	3469	1.40	4.15	1.19
PAK1 T423, PAK2 T402	6134	7280	7010	7283	1.19	1.14	1.19
HER3 Y1289	6681	6477	9907	7862	0.97	1.48	1.18
Musashi	1108	1286	1748	1258	1.16	1.58	1.14
cKit Y719	29053	30806	29568	32965	1.06	1.02	1.13
HER2 Y1248	16267	15455	24858	18186	0.95	1.53	1.12
Cu-Zn SOD	14621	11495	19750	16163	0.79	1.35	1.11
p53 total	15058	18440	15477	16494	1.22	1.03	1.10
p27 T187	5236	8168	6310	5663	1.56	1.21	1.08
Adducin S662	10337	4588	1E+05	10829	0.44	12.36	1.05
Survivin	20401	28320	28816	20665	1.39	1.41	1.01
PAK2 S20	35980	31223	44917	36086	0.87	1.25	1.00
Caspase3, cleaved	13882	16078	15593	13869	1.16	1.12	1.00
PTEN S380	13429	14133	24596	13253	1.05	1.83	0.99
BCL2 S70	5471	6533	5511	5374	1.19	1.01	0.98
Stat 1 Y701	24711	32126	22628	24253	1.30	0.92	0.98
BAD S155	29517	25368	44488	28709	0.86	1.51	0.97
IRS1 S612	3316	4599	6383	3225	1.39	1.92	0.97
CateninB T41/S45	3429	1391	7699	3258	0.41	2.25	0.95
AuroraA T288,AuroraB T232,AuroraC T198,	5736	6858	6258	5125	1.20	1.09	0.89
IKBa S32/36	17121	19103	21905	15263	1.12	1.28	0.89
PARP, cleaved	4156	6320	4285	3662	1.52	1.03	0.88
PYK2 Y402	7915	5789	17059	6883	0.73	2.16	0.87
Histone H3 S10	1571	1673	1460	1365	1.06	0.93	0.87
ATG5 19R	30624	30040	35646	26186	0.98	1.16	0.86
PRK1 T774, PRK2 T816	27625	17677	29954	23565	0.64	1.08	0.85
Biliverdin Reductase B	39898	31977	52747	33724	0.80	1.32	0.85
NUMB	35919	22062	36612	30081	0.61	1.02	0.84
14-3-3zeta-gamma-eta	1417	1403	1276	1184	0.99	0.90	0.84
PLK1 T210	20016	20527	19214	16503	1.03	0.96	0.82
PTEN total	21567	18433	29767	17700	0.85	1.38	0.82
ErbB4_HER4 total	13497	15630	13209	11002	1.16	0.98	0.82
MEK 1/2 S217/221	10154	9993	13543	8039	0.98	1.33	0.79
NFKBp65 S536	8925	11943	7067	6903	1.34	0.79	0.77
IGF1R Y1135/1136, IR Y1150/1151	6199	7554	8833	4780	1.22	1.42	0.77
Shc Y317	15939	16728	17929	12242	1.05	1.12	0.77
BAD S112	33494	27952	36982	25635	0.83	1.10	0.77
Chk1 S345	12118	16190	9286	9147	1.34	0.77	0.75
p70S6K S371	52070	56025	52443	39212	1.08	1.01	0.75
FADD S194	1631	1862	3452	1227	1.14	2.12	0.75
PKCzeta_lamb T410_403	27897	25365	28449	20521	0.91	1.02	0.74
LKB1 S334	12814	14897	10694	9377	1.16	0.83	0.73

Met Y1234/1235	4723	4227	6905	3449	0.89	1.46	0.73
Jak1 Y1022/1023	9800	10261	6861	7110	1.05	0.70	0.73
Tubulin a_b	17703	14905	21447	12460	0.84	1.21	0.70
B-Raf S445	9486	6243	12321	6421	0.66	1.30	0.68
cKit Y703	13525	12997	14302	9116	0.96	1.06	0.67
Raf S259	25855	16733	31873	17354	0.65	1.23	0.67
PKCdelta T505	4452	4378	6480	2950	0.98	1.46	0.66
PKCtheta T538	9863	11406	7941	6499	1.16	0.81	0.66
PDGFRalpha Y754	7297	12213	7975	4637	1.67	1.09	0.64
Bcl2 T56	22206	21819	22072	13991	0.98	0.99	0.63
VEGFR2 Y1175	11848	14852	9131	7095	1.25	0.77	0.60
cRaf S338	11446	8657	7882	6815	0.76	0.69	0.60
A-Raf S299	65395	60864	48243	38760	0.93	0.74	0.59
S6RP S240/244	8380	9667	15734	4949	1.15	1.88	0.59
MMP-11	6157	6230	5359	3537	1.01	0.87	0.57
SGK S78	10082	10577	8200	5565	1.05	0.81	0.55
PDK1 S241	30564	19677	31746	16836	0.64	1.04	0.55
Histone H3 S28	30159	17567	47009	16247	0.58	1.56	0.54
Stat3 S727	17761	17123	20426	9081	0.96	1.15	0.51
LC3B	24805	23591	23171	12426	0.95	0.93	0.50
LIMK1 T508, LIMK2 T505	3044	3381	3265	1509	1.11	1.07	0.50
SAPK/JNK T183/Y185	4159	4326	3231	1981	1.04	0.78	0.48
VEGFR2 Y996	10803	6744	11171	5102	0.62	1.03	0.47
Heme-Oxygenase 1	14727	26102	10872	6931	1.77	0.74	0.47
PLCgamma1 Y783	12068	10155	10849	5425	0.84	0.90	0.45
GFAP	10653	5690	11475	4708	0.53	1.08	0.44
VEGFR2 Y951	6502	7963	5162	2781	1.22	0.79	0.43
ERK 1/2 T202/Y204	7345	10151	4256	3024	1.38	0.58	0.41
Elk1 S383	14121	14734	10346	5426	1.04	0.73	0.38
ASK1 S83	17449	47767	23463	6642	2.74	1.34	0.38
Cofilin total	26772	17000	25593	9972	0.63	0.96	0.37
Smad2 S245/250/255	9456	5429	6505	3143	0.57	0.69	0.33
eIF4G S1108	10399	7978	12707	3407	0.77	1.22	0.33
Ret Y905	12739	8427	9951	4173	0.66	0.78	0.33
Crk Y221	4103	4506	6312	1278	1.10	1.54	0.31
RSK3 T356/S360	8521	11928	6327	1507	1.40	0.74	0.18
Smad1 S463/465,Smad5 S463/465,Smad8 S426/428	24131	9437	11419	3830	0.39	0.47	0.16
ErbB3_HER3 total	41324	16850	19307	6086	0.41	0.47	0.15
